# Supplementary material for: The Gastrodia elata genome provides insights into plant adaptation to heterotrophy
Source: Nat Commun. 2018 Apr 24;9:1615. doi: 10.1038/s41467-018-03423-5 (PMC5915607; doi:10.1038/s41467-018-03423-5)
Supplement: Supplementary file 1 — Supplementary Information [file 41467_2018_3423_MOESM1_ESM.pdf]

Yuan et al. The *Gastrodia elata* genome provides insights into plant adaptation to heterotrophy

SI GUIDE

File Name: Supplementary Information

Description: Supplementary Figures, Supplementary Tables, Supplementary Methods, Supplementary References, and Supplementary Note.

Supplementary Figures

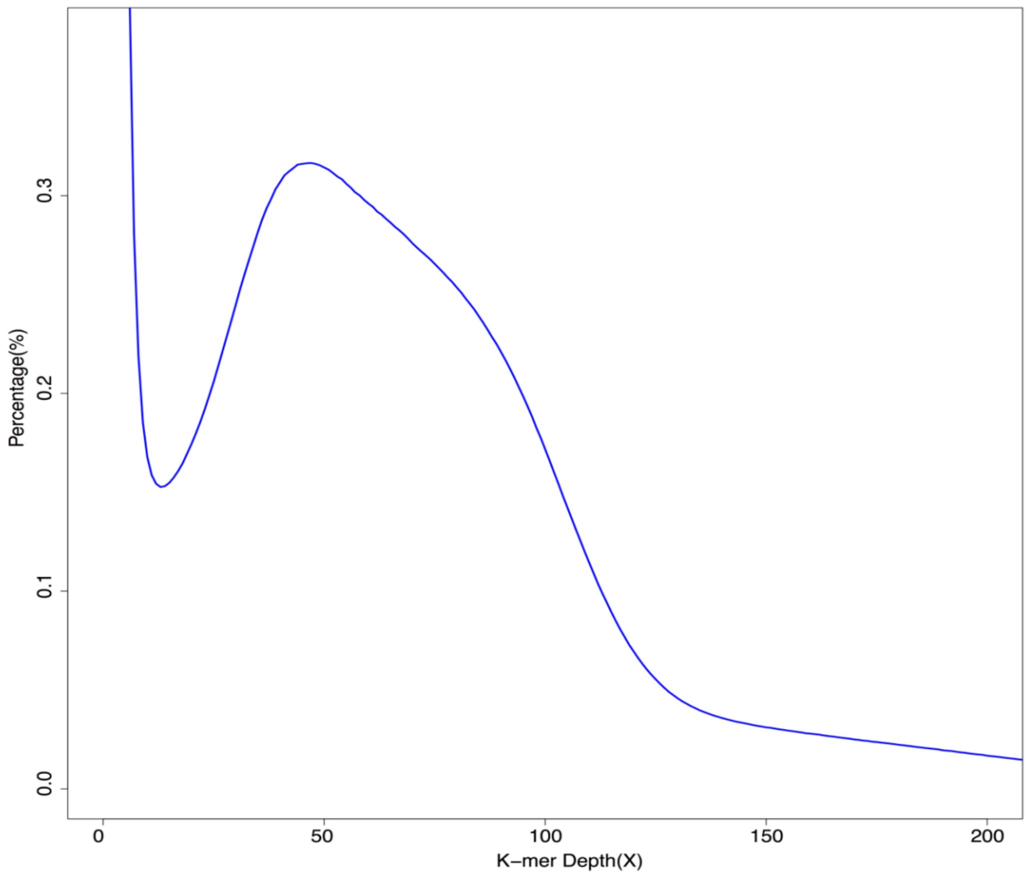

Supplementary Figure 1 | K-mer analysis for estimating the genome size of *Gastrodia elata*. The paired-end reads from short insert-size libraries (230 bp) were used to generate the 17-mer frequency curve. The horizontal axis represented the K-mer depth, namely the number of times occurred. The volume of K-mers was plotted against the frequency at which they occur. The left-hand peak at low frequency and high volume represented K-mers containing essentially random sequencing errors. The main volume peak of K-mers was 47. Using the formulate Genome size = (total number of k-mers) / (the volume peak), the genome size of *G. elata* was estimated to be 1.18 Gb.

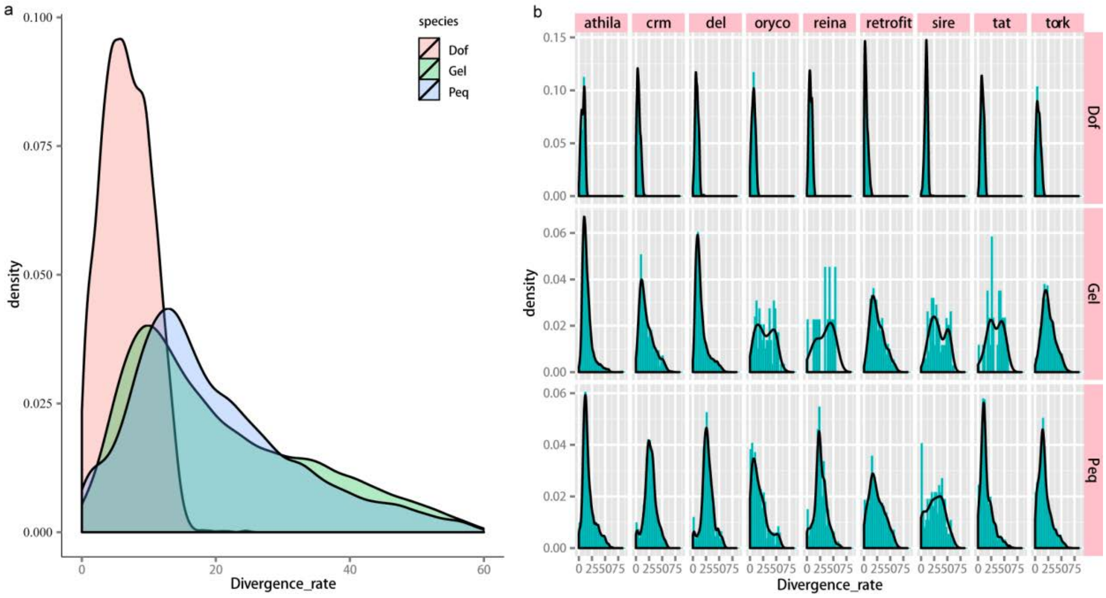

Supplementary Figure 2 | Comparison of LTR (long terminal repeat retroelement) dynamics in *G. elata* (Gel), *Phalaenopsis equestris* (Peq) and *Dendrobium officinale* (Dof). (a) Divergence rate of all intact LTRs. 5'- and 3'-LTR sequences of the intact LTR were aligned and used to calculate the Kimura distances. The Kimura distance was estimated through combination of transition rate and transversion rate, using the formula (K = -(1/2) ln(1-2p-q)-(1/4) ln(1-2q)). 'p' is the proportion of transition site and 'q' is the proportion of transversion site. Using Kimura distances, relative age of different TE types in the genome was plotted. The density curve represented LTR expansion at different divergence rate of the *G. elata* (Gel), *P. equestris* (Peq) and *D. officinale* (Dof) genome. **(b)** Divergence rate of LTR families with copy number > 100. Family names were presented on top of the figure.

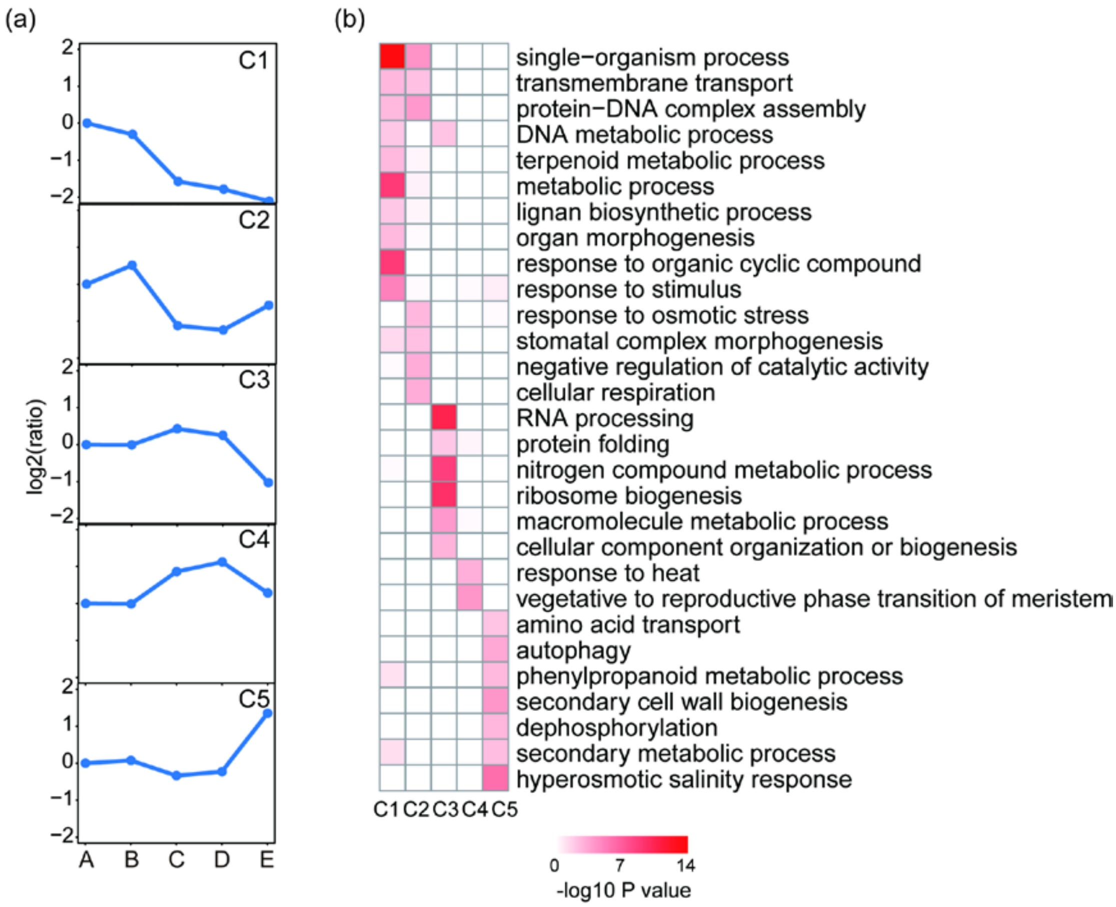

Supplementary Figure 3 | Dynamic progression of *G. elata* transcriptome. (a) The expression pattern of each cluster (C1-C5), each cluster contain genes that have similar expression patterns (co-expression) in five growth stages of *G. elata* (**Figure 1a**, A-E). For each growth stage, average expression value of genes in each cluster were shown (points in the plot). **(b)** GO category enrichment analysis of the five co-expression modules (C1-C5), only shown GO categories that were significant enriched (Fisher's exact test, with a false discovery rate (FDR) < 0.01). Non-redundant GO term set were obtained to assist in interpretation using Sim_{Rel} score, a scoring scheme to measure similarities between GO terms[1].

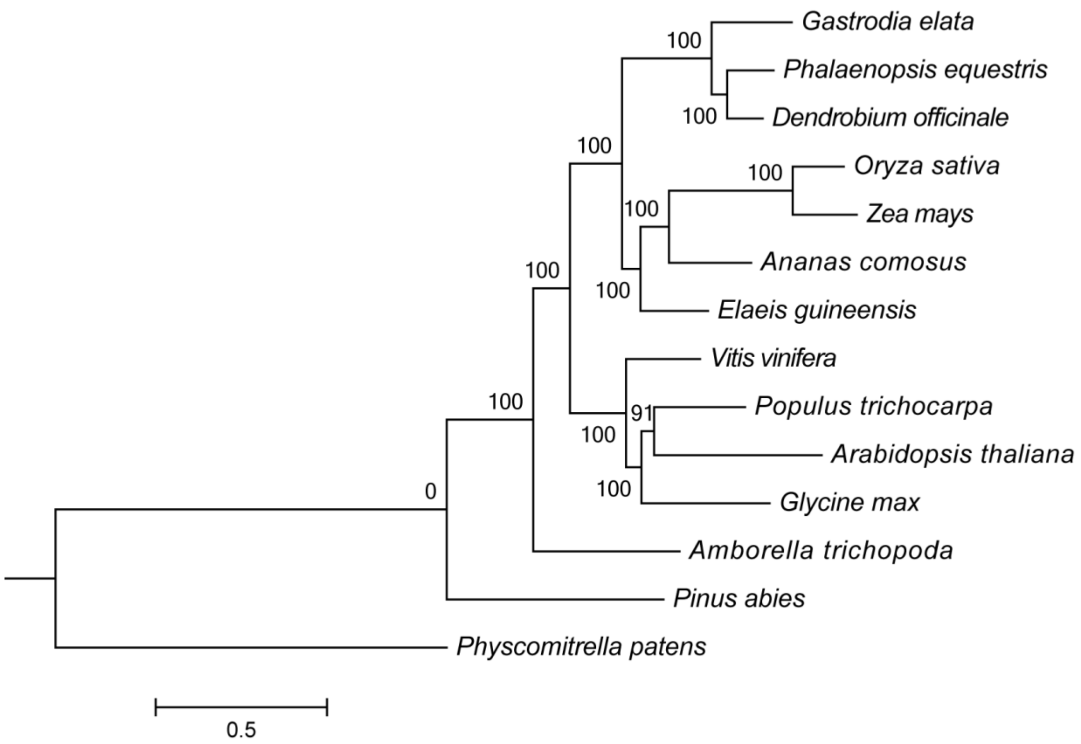

Supplementary Figure 4 | The phylogenetic tree shows the topology and bootstrap values for 14 plant species. MUSCLE was used to generate multiple sequence alignment for protein sequences in single-copy families with default parameters. The alignments were combined into a supermatrix using in-house perl script. Prottest was used to select the best-fit model (JTT+I+G+F) for amino acid replacement. RAxML (PROTCATJTT) was used to construct phylogenetic tree with maximum-likelihood algorithms.

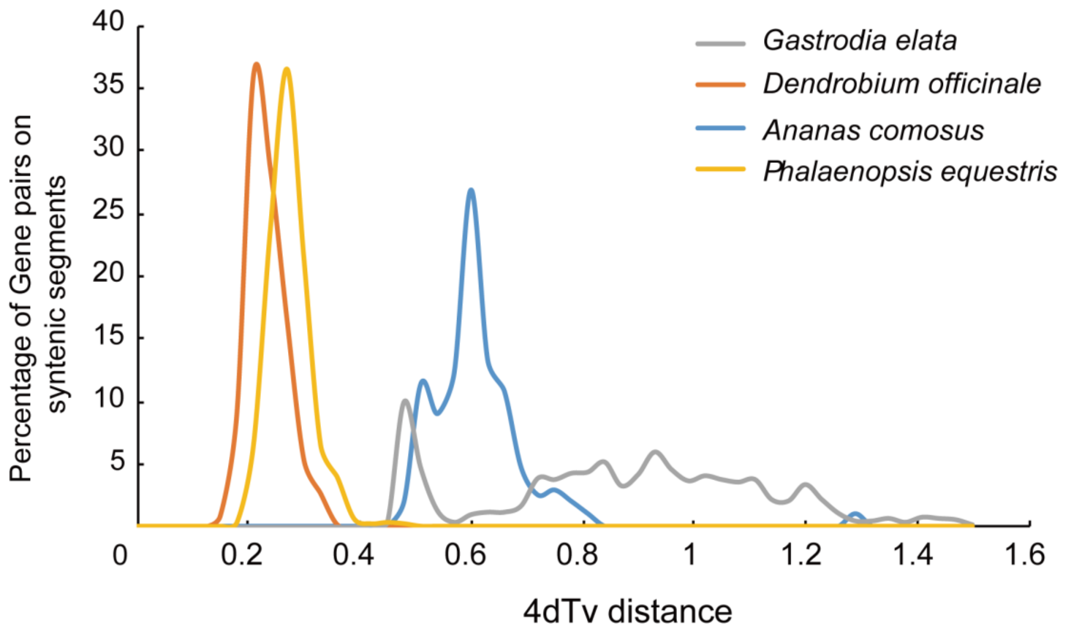

Supplementary Figure 5 | Whole genome duplication (WGD) events detected in genome of *G. elata*, *D. officinale*, *Ananas comosus* and *P. equestris*. 4dTv, transversion substitutions at fourfold degenerate sites.

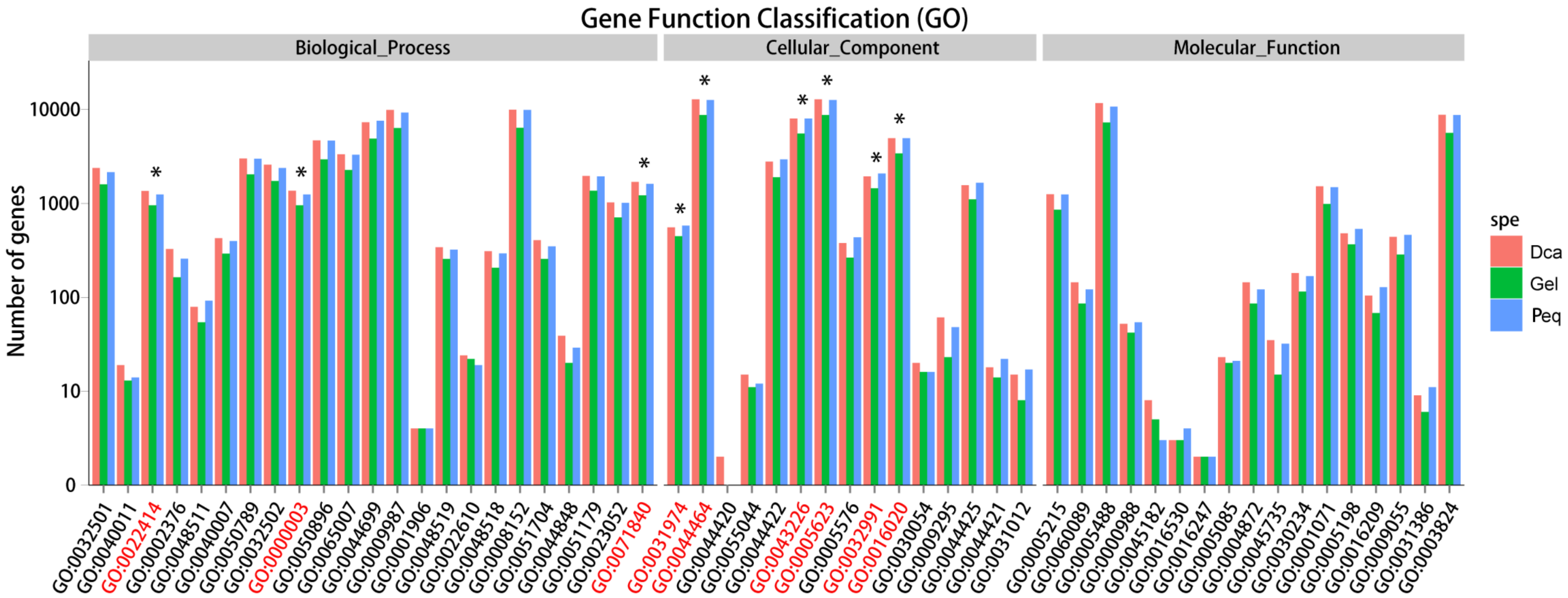

Supplementary Figure 6 | **Comparison of gene number in second level GO terms between three orchid genomes.** Asterisk represent gene number significant reduced in *G. elata* (Fisher's exact test, $p < 0.05$); Dca, *D. officinale*; Gel, *G. elata*; Peq, *P. equestris*.

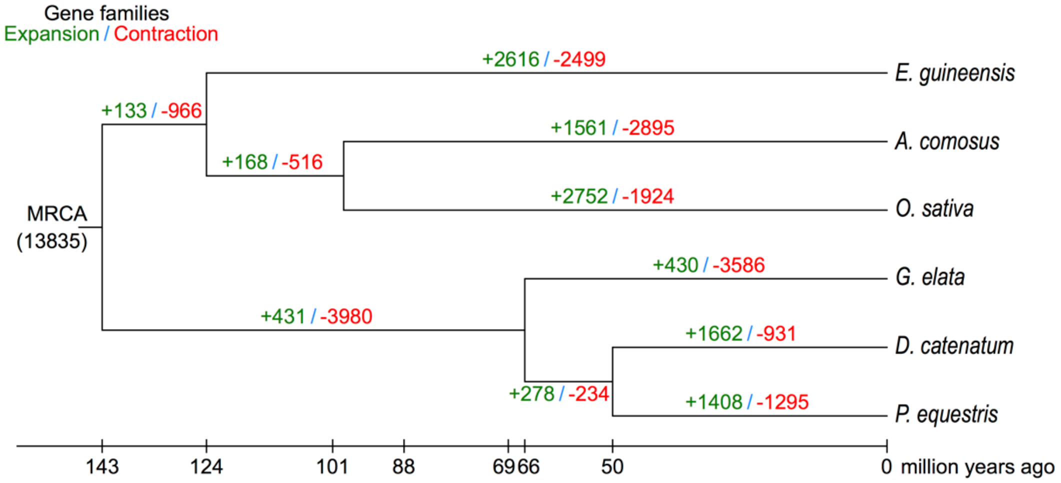

Supplementary Figure 7 | Expansion and contraction in gene families. Numbers deignate the number of gene families that have expanded (green) and contracted (red) since the split from the common ancestor. MRCA, most recent common ancestor.

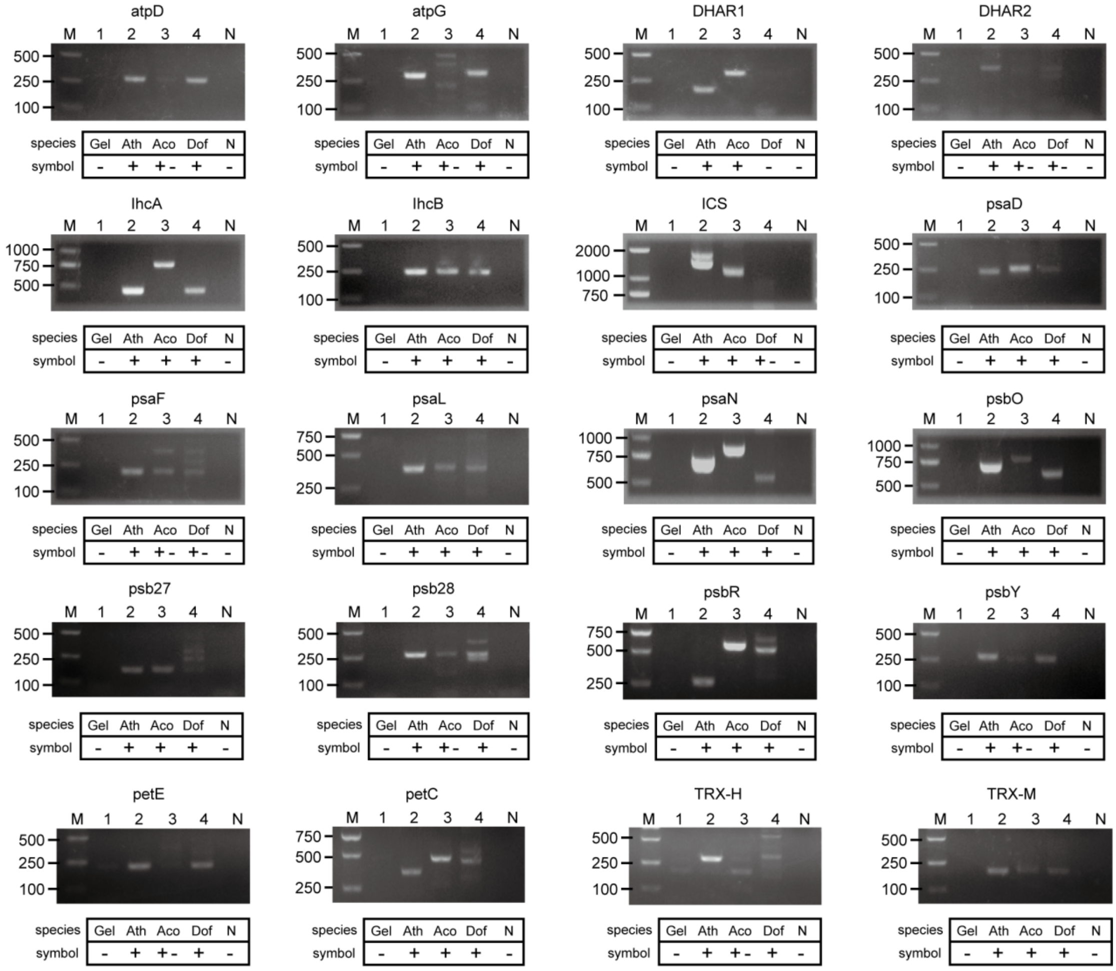

Supplementary Figure 8 | PCR verification of the loss genes of *G. elata*. Photosynthesis related genes: *atp*D, *atp*G, *Ihc*A, *Ihc*B, *psa*D, *psa*F, *psa*L, *psa*N, *psb*O, *psb*R, *psb*Y, *psb*27, *psb*28, *pet*C, *pet*E; Defense related genes: *ICS* (Isochorismate synthase), *DHAR* (Dehydroascorbate reductase), *TRX-H* (Thioredoxin H-type), *TRX-M* (Thioredoxin M-type). M - Marker; 1 – *G. elata*; 2 - *Arabidopsis thaliana*; 3 – *A. comosus*; 4 – *D. officinale*; N - negative control.

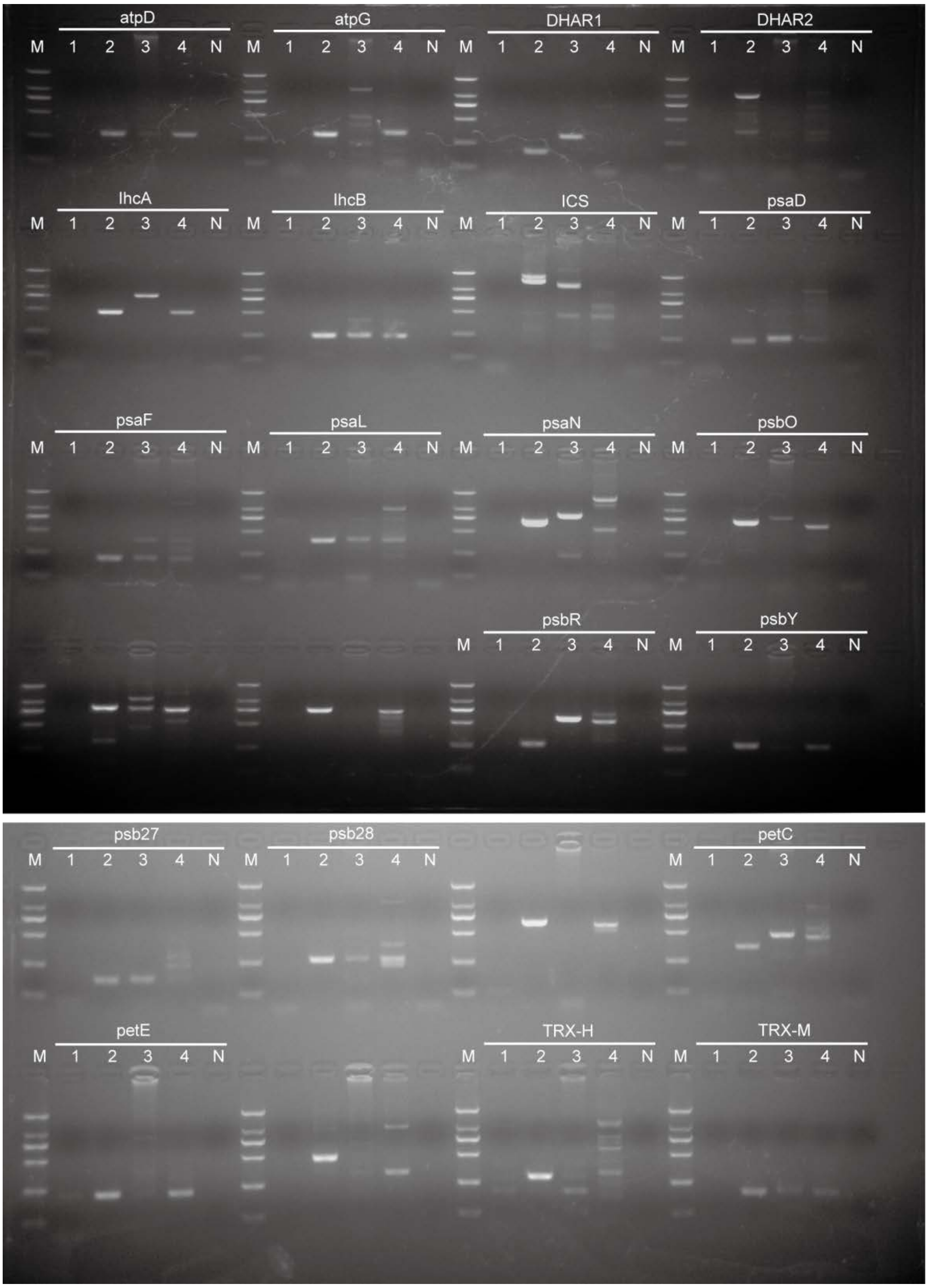

Supplementary Figure 9 | PCR verification of the loss genes of *G. elata* (uncropped gels in supplementary figure 8). M - Marker; 1 – *G. elata*; 2 - *Arabidopsis thaliana*; 3 – *A. comosus*; 4 – *D. officinale*; N - negative control.

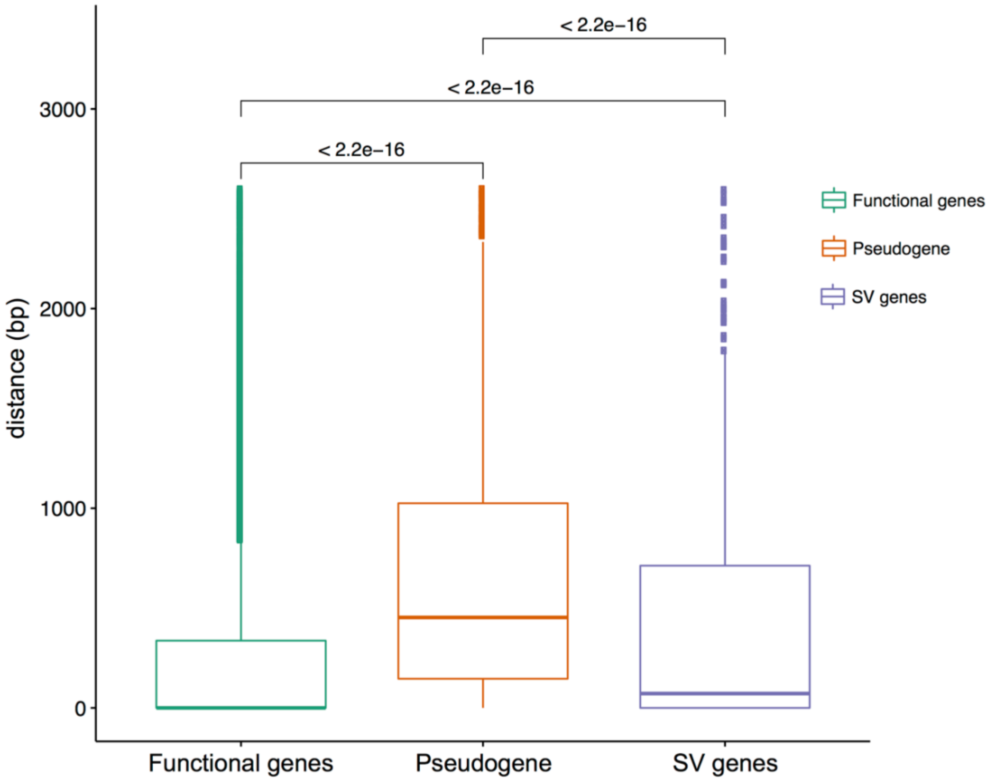

Supplementary Figure 10 | Potential impact of transposable elements (TEs) on *G. elata* gene loss process. Green, distance to the closest TE for all *G. elata* functional genes; Orange, distance to the closest TE for all pseudogenes; Purple, distance to the closest TE for all rearranged loacations that causing gene loss.

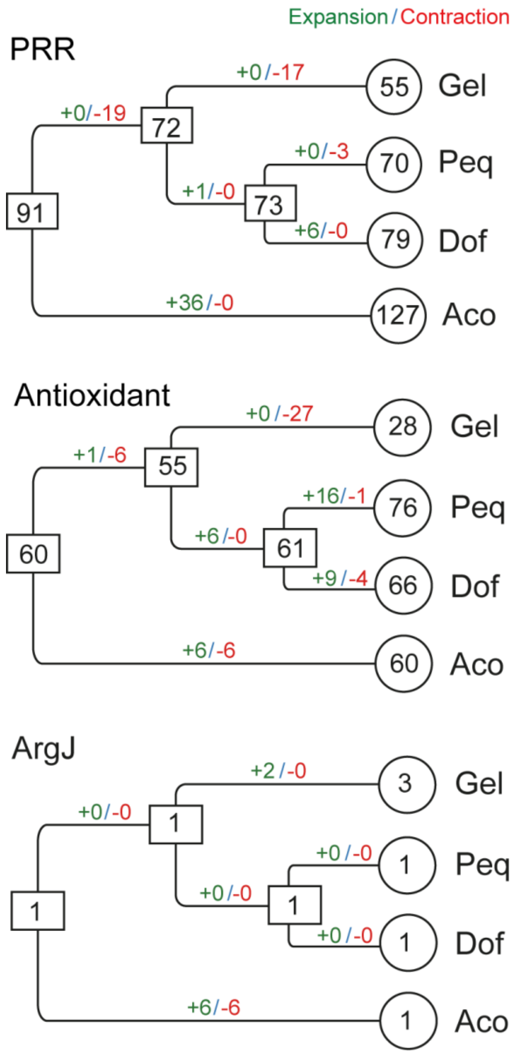

Supplementary Figure 11 | Analysis of gene numbers of Pattern Recognition Receptor (PRR) genes, Antioxidant related proteins, and glutamate N-acetyltransferases (ArgJ) in *G. elata* (Gel), *P. equestris* (Peq), *D. officinale* (Dof) and *A. comosus* (Aco) genome. Numbers in circles represent the number of family member in each genome; numbers with plus and minus signs indicate the number of duplicated and deleted genes, respectively.

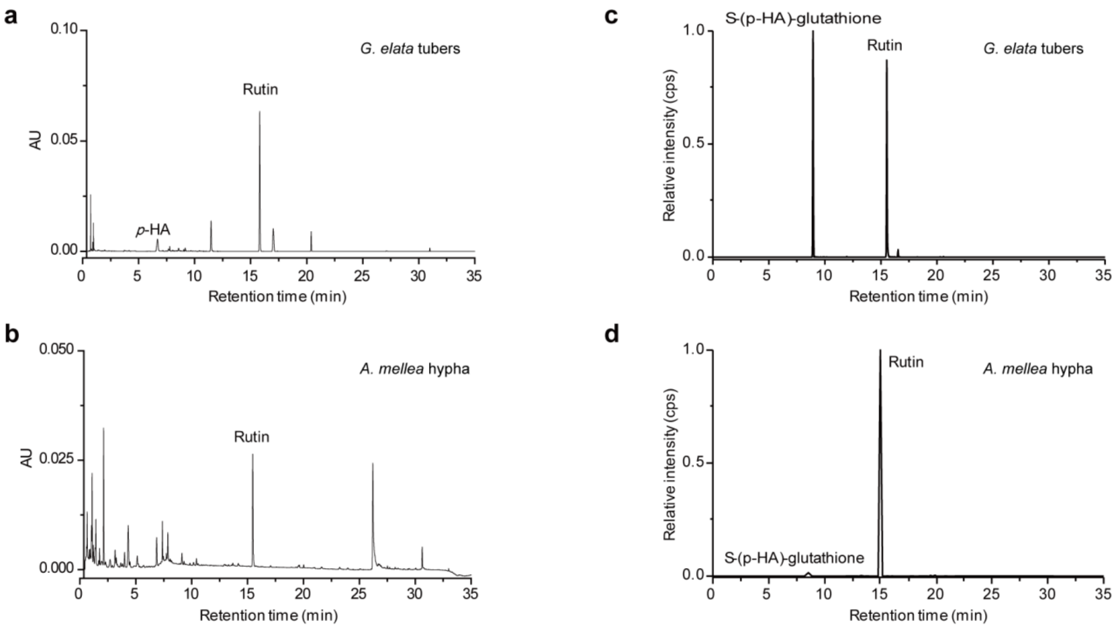

Supplementary Figure 12 | Relative content of phytoalexin related compounds (4-Hydroxybenzyl alcohol (*p*-HA) and *S*-(*p*-HA)-glutathione) in *G. elata* tubers and *A. mellea* hypha. (a) The chromatogram detected at 270 nm of *p*-HA in *G. elata* tubers. **(b)** The chromatogram detected at 270 nm of *p*-HA in *A. mellea* hypha. **(c)** The extract ion chromatogram of *S*-(*p*-HA)-glutathione in *G. elata* tubers. **(d)** The extract ion chromatogram of *S*-(*p*-HA)-glutathione in *A. mellea* hypha.

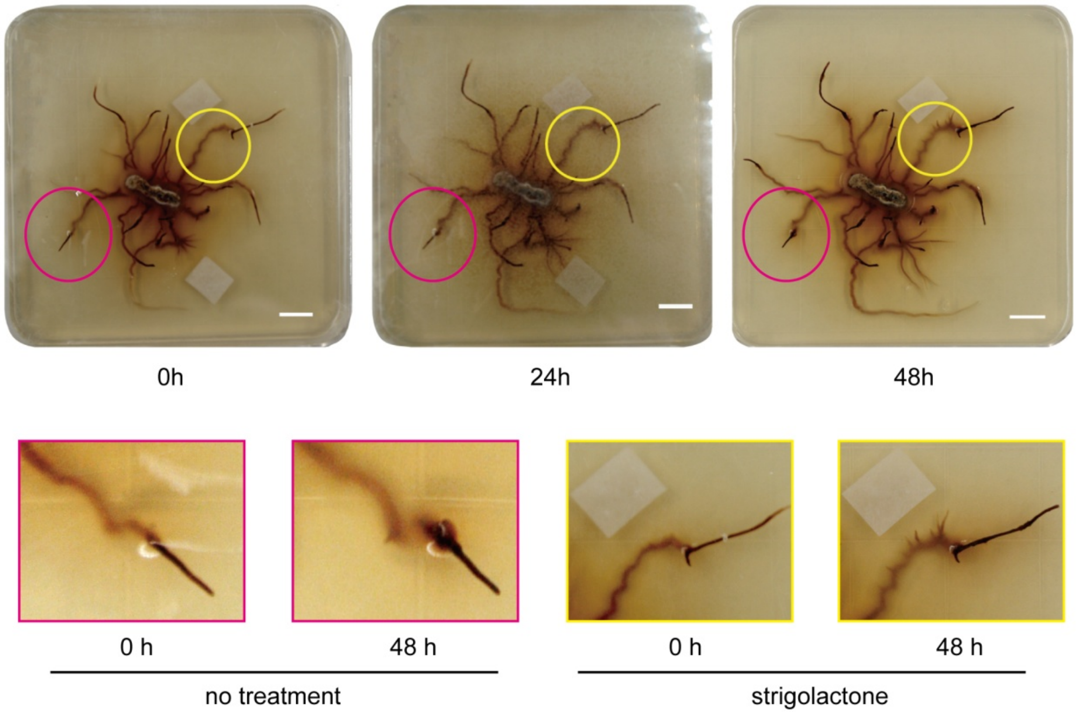

Supplementary Figure 13 | Hyphae branching of *A. mellea* after strigolactone treatment. Scale bar was 1 cm.

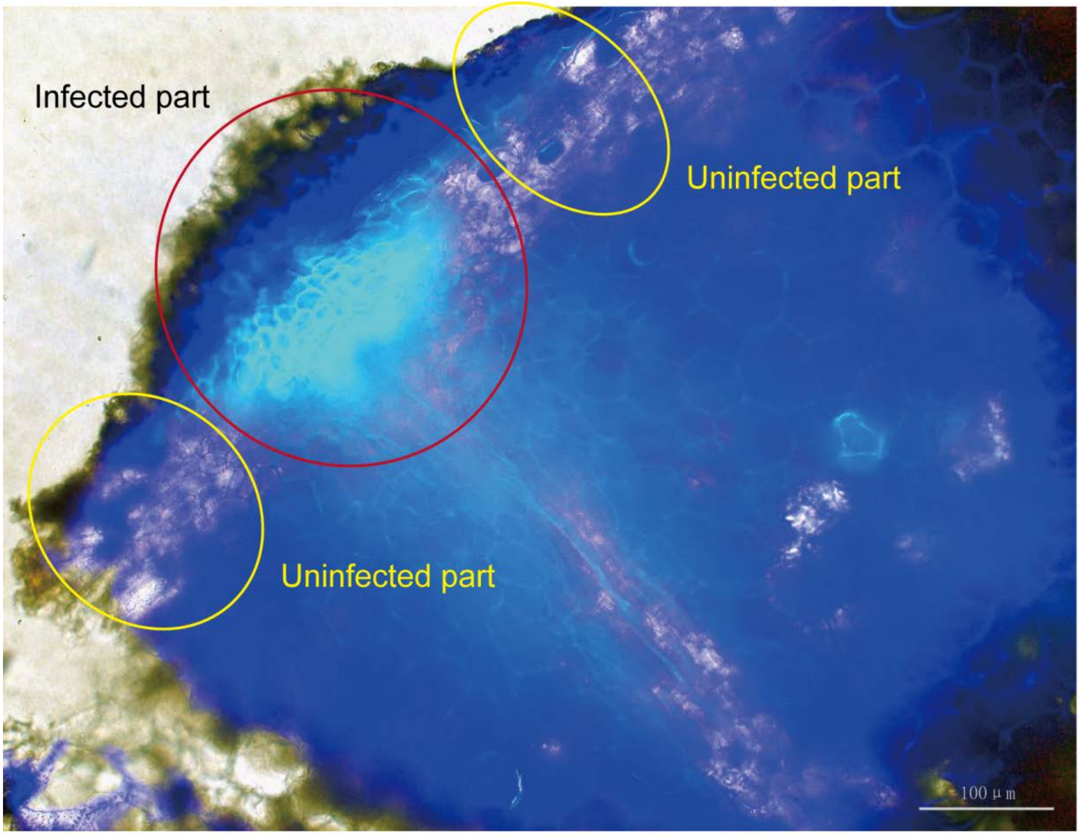

Supplementary Figure 14 | Cross sections and micrographs of immature *G. elata* tubers in association with *A. mellea*. Scale bar was 100 μm.

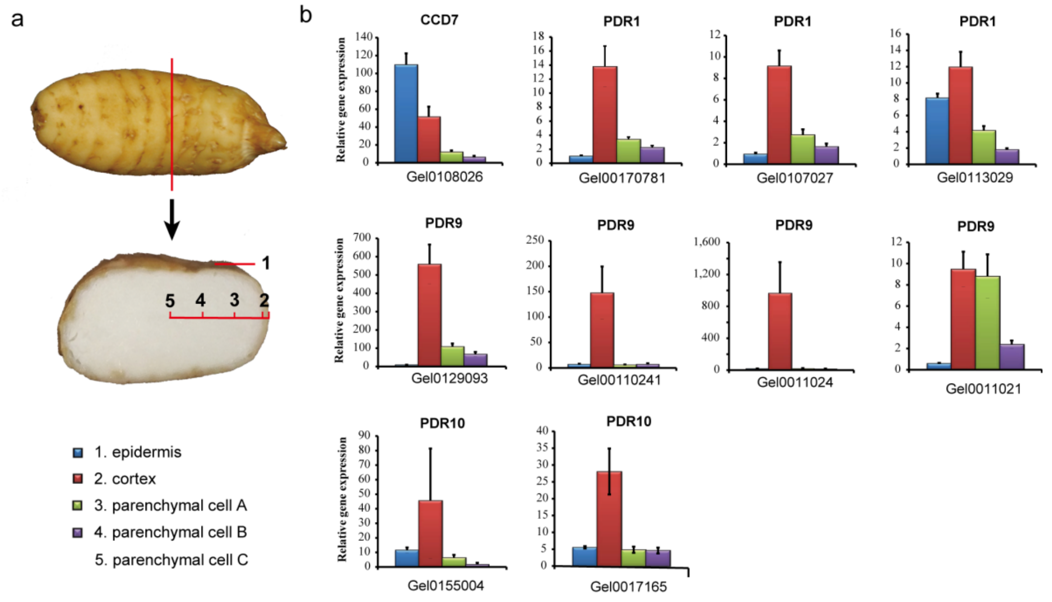

Supplementary Figure 15 | Strigolactone as signaling molecule in the association between *G. elata* and *A. mellea*. (a) Different sections of mature tuber of *G. elata*. **(b)** Gene expression of CCD7 and PDRs in different sections of mature tuber of *G. elata* (6 biological replicates and 3 technical replicates; error bar showed the standard deviation of gene expressions of 6 biological replicates).

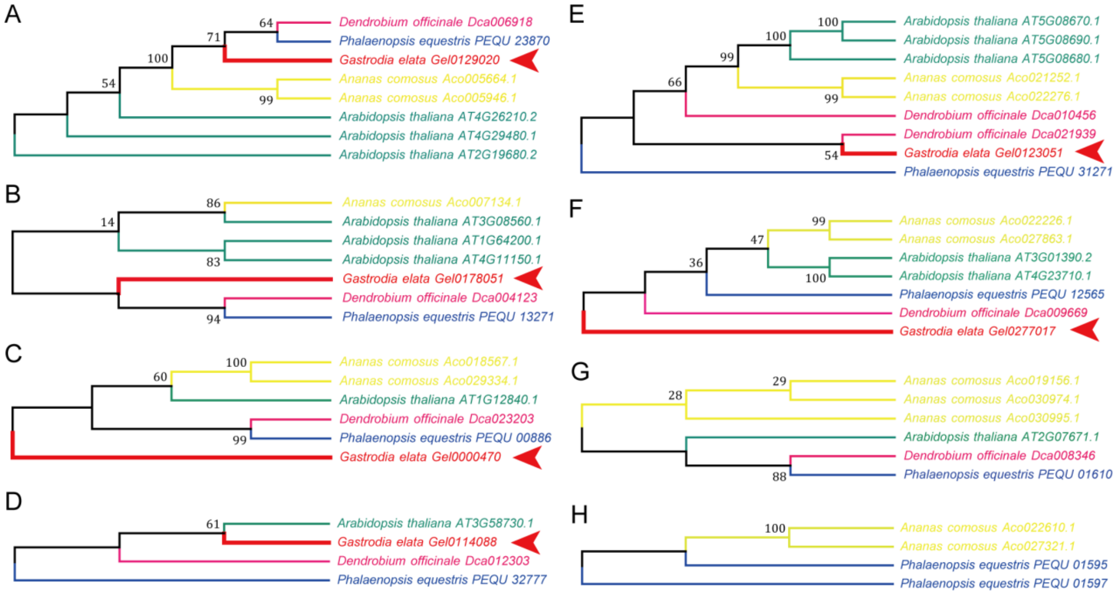

Supplementary Figure 16 | A phylogenetic neighbor-joining tree constructed from the photosynthetic gene ATPsyn of *G. elata*, *A. thaliana*, *A. comosus*, *D. officinale*, and *P. equestris*. ATPsyn members of *G. elata* are marked with rose lines and arrow head. ATPsyn *genes* of *A. thaliana*, *A. comosus*, *D. officinale*, and *P. equestris* are respectively marked with green, yellow, pink and blue line. (A) Subfamily of mitochondrial ATP synthase subunit G. (B) Subfamily of vacuolar H^+-ATPase subunit E. (C) Subfamily of vacuolar ATP synthase subunit C. (D) Subfamily of vacuolar ATP synthase subunit D. (E) Subfamily of ATP synthase alpha/beta family protein. (F) Subfamily of vacuolar ATP synthase subunit G. (G) Subfamily of ATP synthase subunit C family protein. (H) Subfamily of ATP-synt_ab.

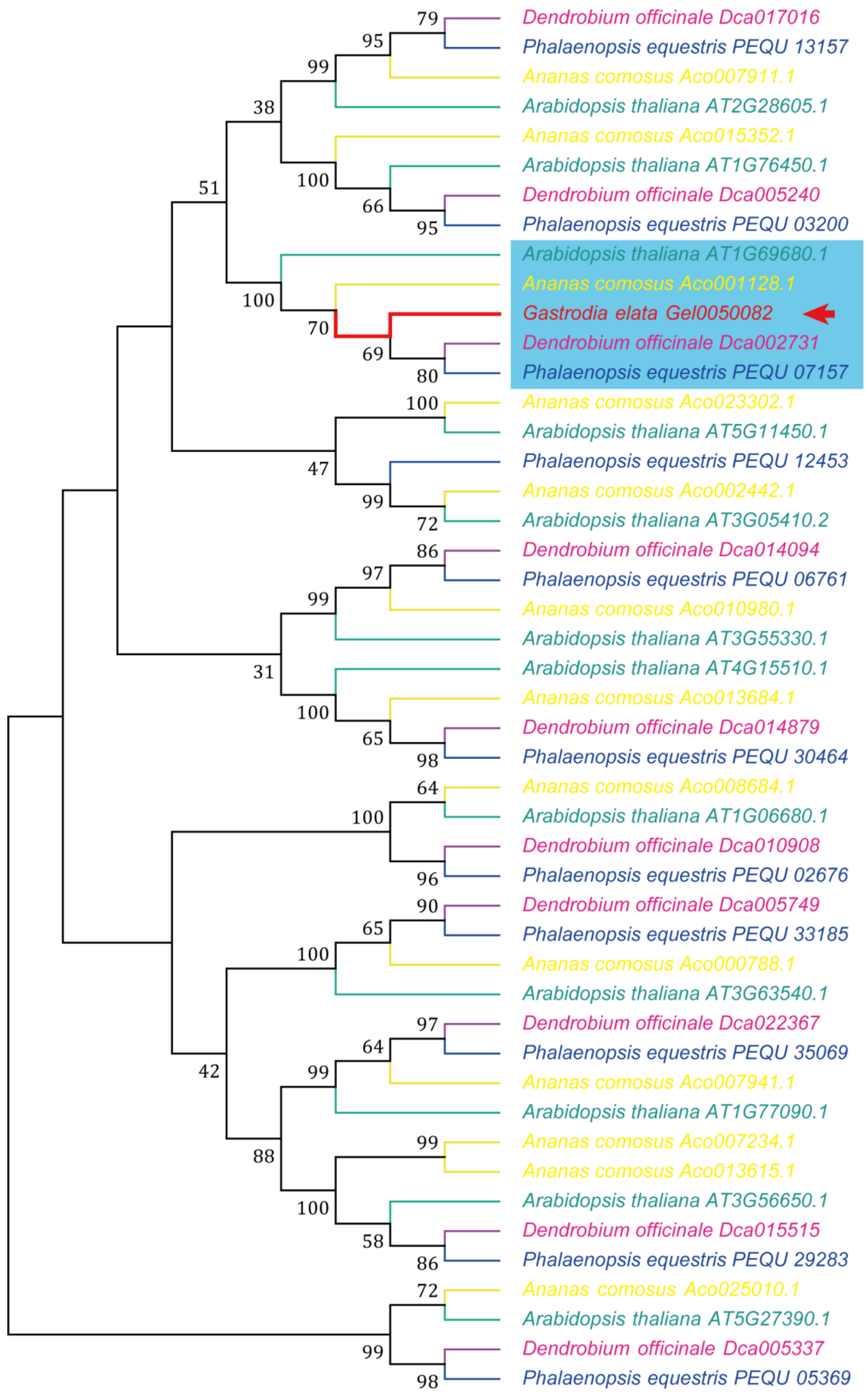

Supplementary Figure 17 | A phylogenetic neighbor-joining tree constructed from the photosynthetic gene *psb*P of *G. elata, A. thaliana, A. comosus, D. officinale,* and *P. equestris*. *psb*P gene of *G. elata* is marked with rose lines and arrow head. *psb*P *genes* of *A. thaliana*, *A. comosus*, *D. officinale*, and *P. equestris* are respectively marked with green, yellow, pink and blue line.

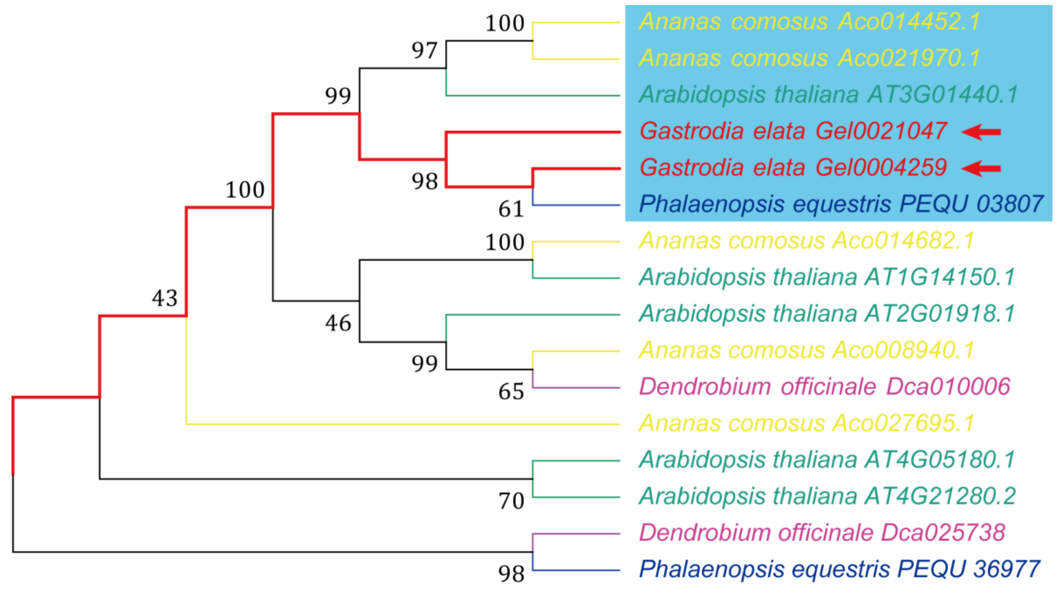

Supplementary Figure 18 | A phylogenetic neighbor-joining tree constructed from the photosynthetic gene *psb*Q of *G. elata*, *A. thaliana*, *A. comosus*, *D. officinale*, and *P. equestris*. *psb*Q gene of *G. elata* is marked with rose lines and arrow head. *psb*Q *genes* of *A. thaliana*, *A. comosus*, *D. officinale*, and *P. equestris* are respectively marked with green, yellow, pink and blue line.

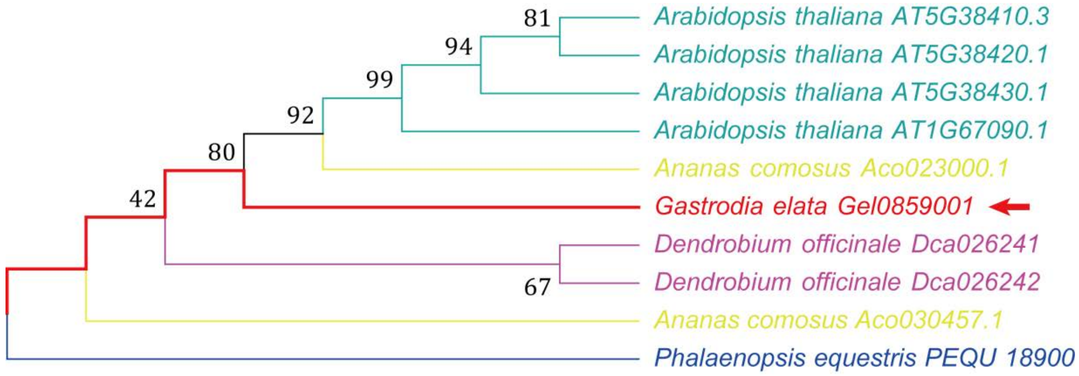

Supplementary Figure 19 | A phylogenetic neighbor-joining tree constructed from the photosynthetic gene *rbc*S of *G. elata, A. thaliana, A. comosus, D. officinale*, and *P. equestris*. *rbc*S gene of *G. elata* is marked with rose lines and arrow head. *rbc*S *genes* of *A. thaliana, A. comosus*, *D. officinale*, and *P. equestris* are respectively marked with green, yellow, pink and blue line.

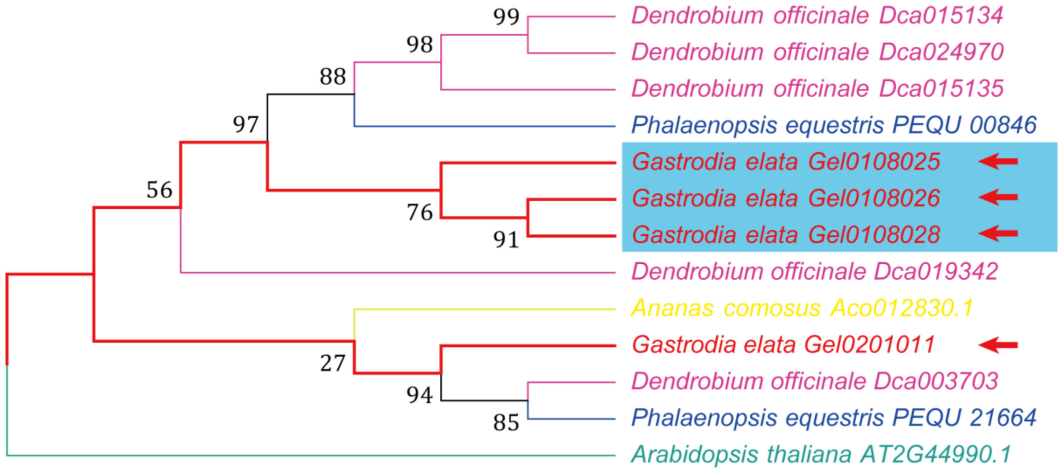

Supplementary Figure 20 | A phylogenetic neighbor-joining tree constructed from *CCD7* involved in strigolactone synthesis of *G. elata*, *A. thaliana*, *A. comosus*, *D. officinale*, and *P. equestris*. *CCD7* gene of *G. elata* is marked with rose lines and arrow head. *CCD7 genes* of *A. thaliana*, *A. comosus*, *D. officinale*, and *P. equestris* are respectively marked with green, yellow, pink and blue line.

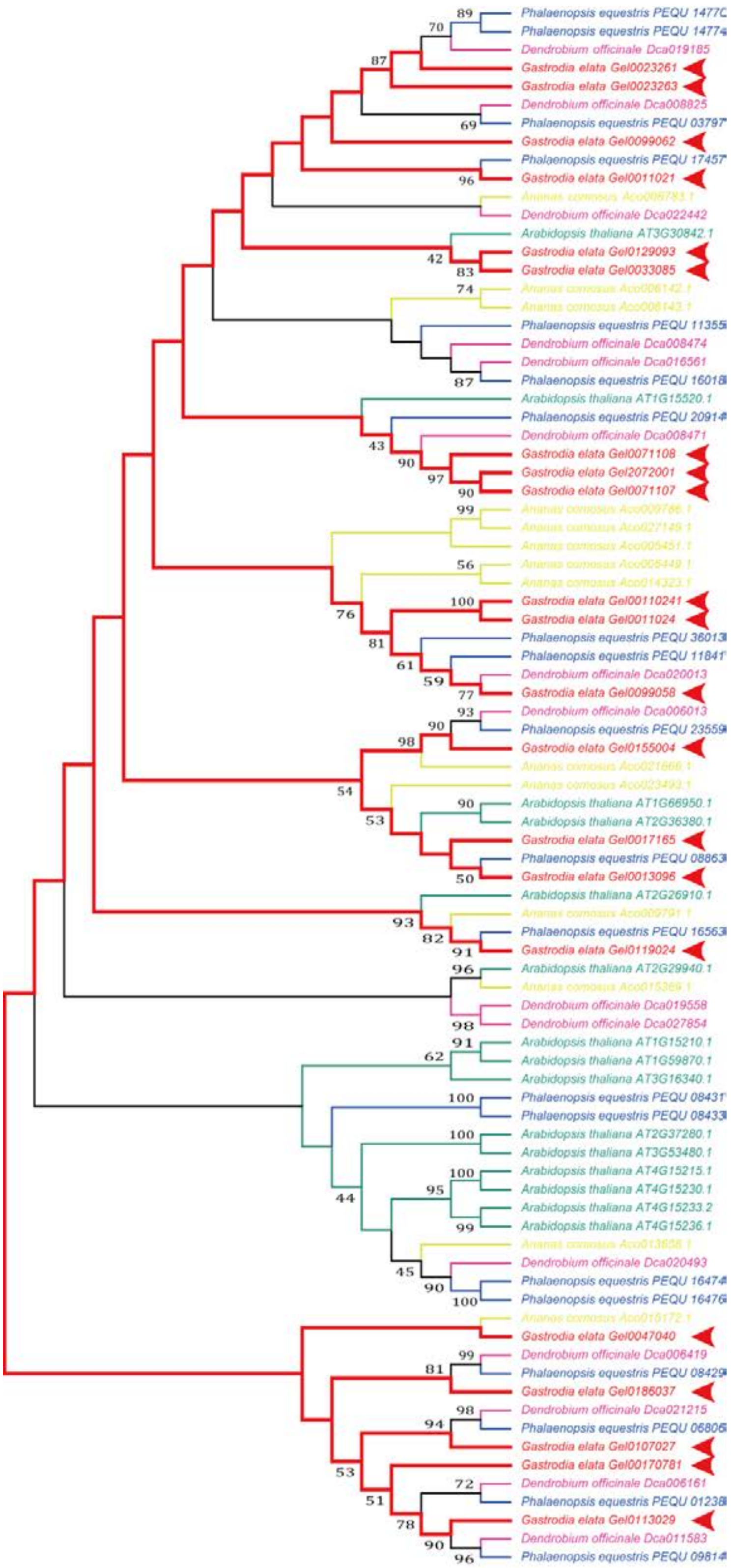

Supplementary Figure 21 | A phylogenetic neighbor-joining tree constructed from *PDR* involved in strigolactone synthesis of *G. elata*, *A. thaliana*, *A. comosus*, *D. officinale*, and *P. equestris*. *PDR* gene of *G. elata* is marked with rose lines and arrow head. *PDR genes* of *A. thaliana*, *A. comosus*, *D. officinale*, and *P. equestris* are respectively marked with green, yellow, pink and blue line.

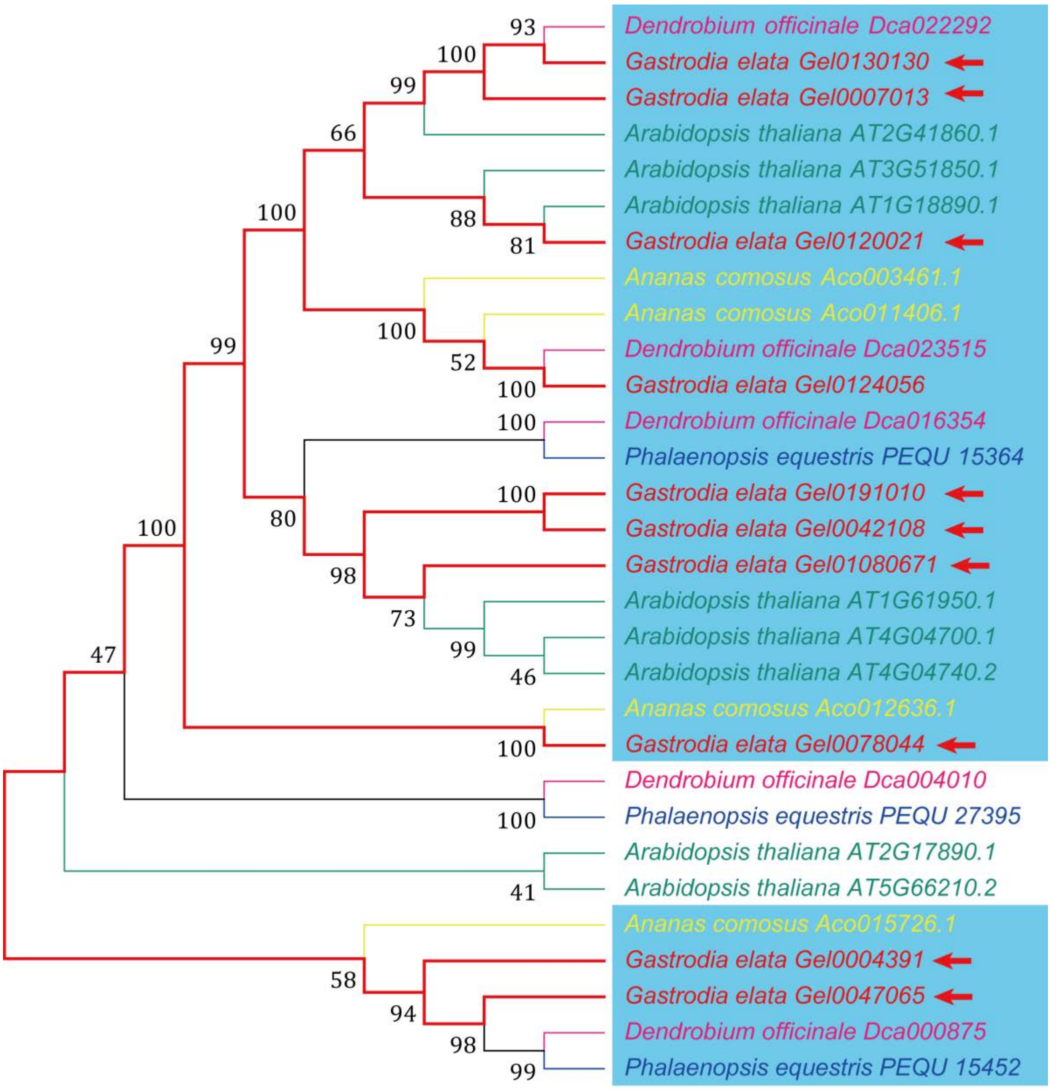

Supplementary Figure 22 | A phylogenetic neighbor-joining tree constructed from signal related gene of *DMI3* of *G. elata, A. thaliana, A. comosus, D. officinale*, and *P. equestris*. *DIM3* gene of *G. elata* is marked with rose lines and arrow head. *DIM3 genes* of *A. thaliana*, *A. comosus*, *D. officinale*, and *P. equestris* are respectively marked with green, yellow, pink and blue line.

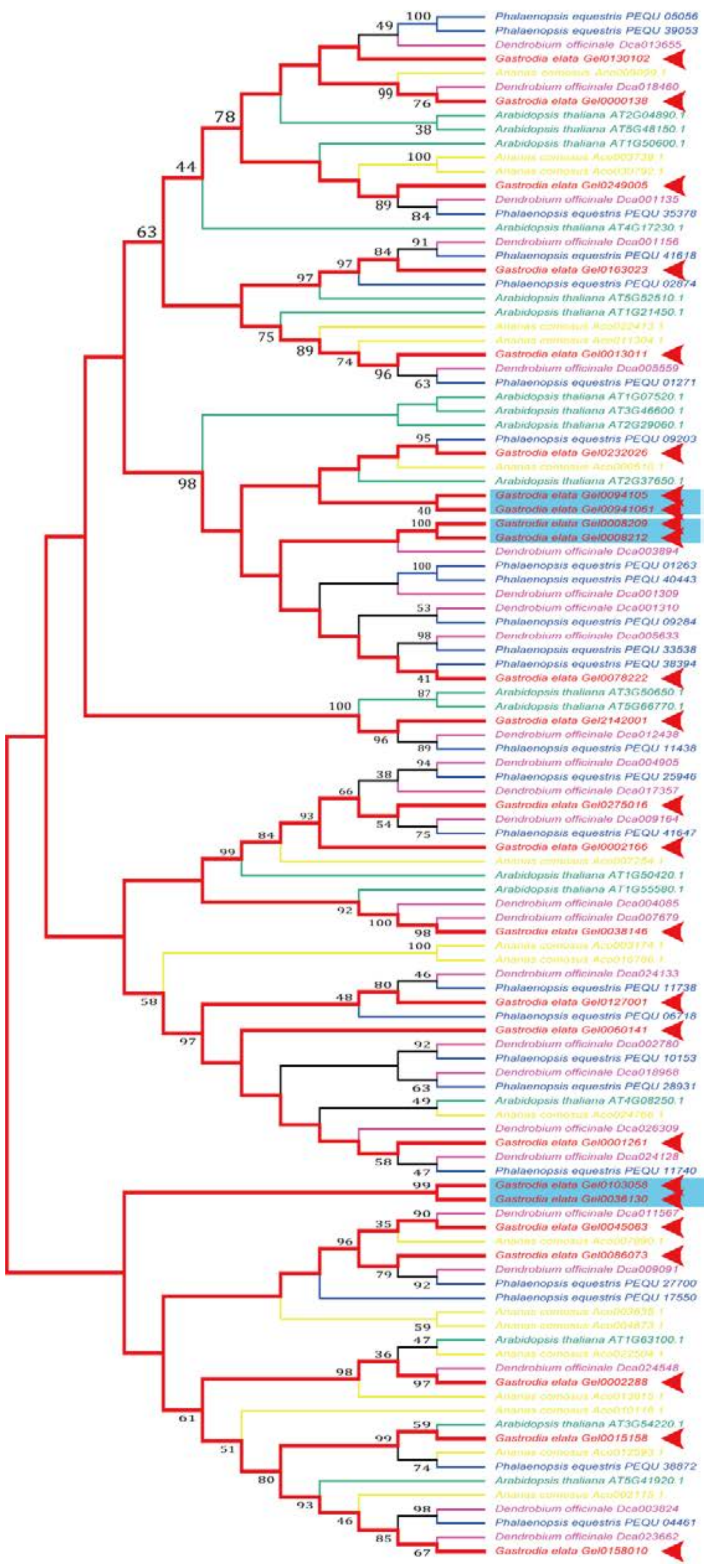

Supplementary Figure 23 | A phylogenetic neighbor-joining tree constructed from signal related gene of *RAM1* of *G. elata*, *A. thaliana*, *A. comosus*, *D. officinale*, and *P. equestris*. *RAM1* gene of *G. elata* is marked with rose lines and arrow head. *RAM1 genes* of *A. thaliana*, *A. comosus*, *D. officinale*, and *P. equestris* are respectively marked with green, yellow, pink and blue line.

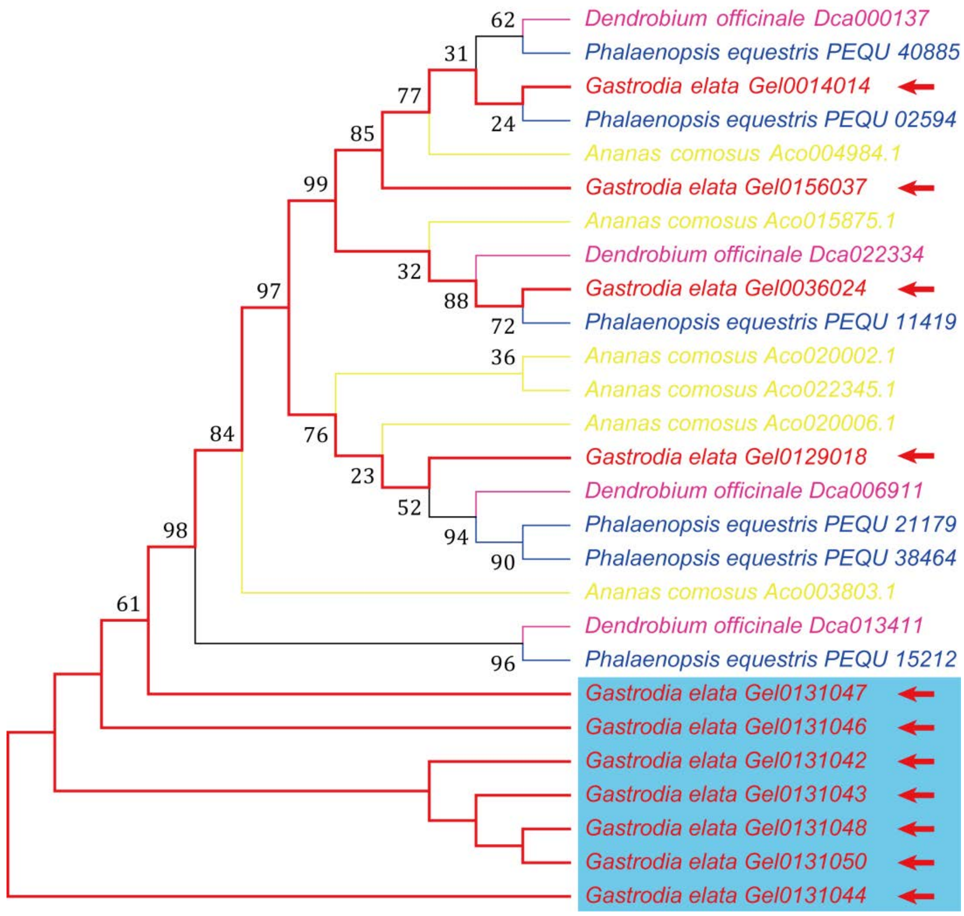

Supplementary Figure 24 | A phylogenetic neighbor-joining tree constructed from glycoside hydrolase gene of endo-*β*-1,4-D-xylanase of *G. elata*, *A. thaliana*, *A. comosus*, *D. officinale*, and *P. equestris*. Endo-*β*-1,4-D-xylanase gene of *G. elata* is marked with rose lines and arrow head. Endo-β-1,4-D-xylanase *genes* of *A. thaliana*, *A. comosus*, *D. officinale*, and *P. equestris* are respectively marked with green, yellow, pink and blue line.

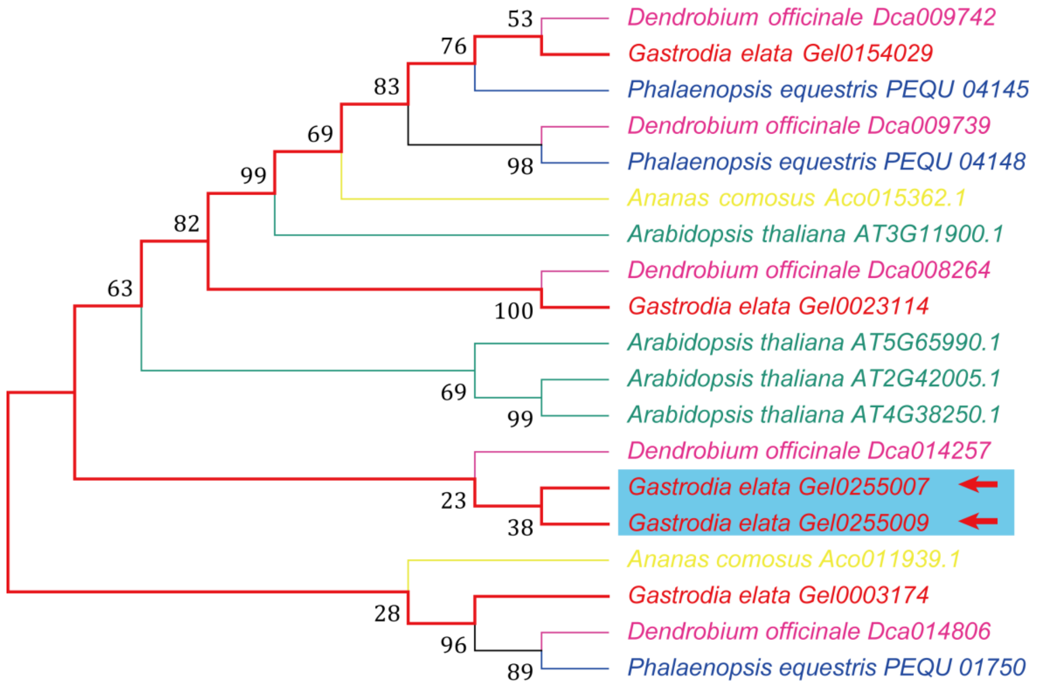

Supplementary Figure 25| A phylogenetic neighbor-joining tree constructed from amino acid transporter gene of *ANT* of *G. elata*, *A. thaliana*, *A. comosus*, *D. officinale*, and *P. equestris*. *ANT* gene of *G. elata* is marked with rose lines and arrow head. *ANT genes* of *A. thaliana*, *A. comosus*, *D. officinale*, and *P. equestris* are respectively marked with green, yellow, pink and blue line.

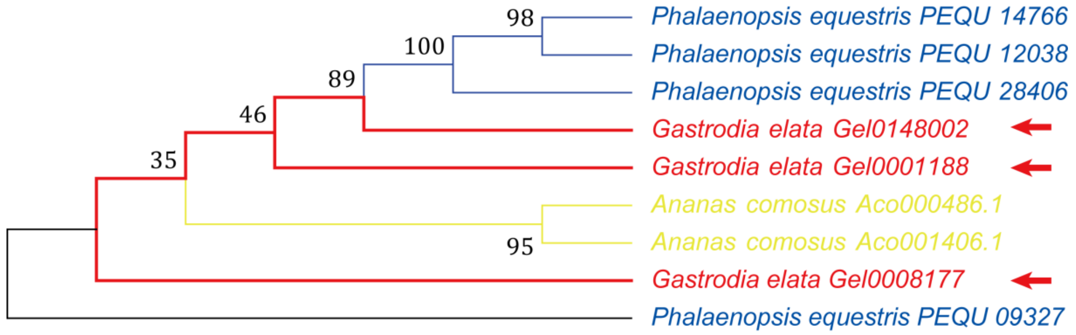

Supplementary Figure 26 | A phylogenetic neighbor-joining tree constructed from amino acid transporter gene of *PROT* of *G. elata, A. thaliana, A. comosus, D. officinale,* and *P. equestris*. *PROT* gene of *G. elata* is marked with rose lines and arrow head. *PROT genes* of *A. thaliana*, *A. comosus*, *D. officinale*, and *P. equestris* are respectively marked with green, yellow, pink and blue line.

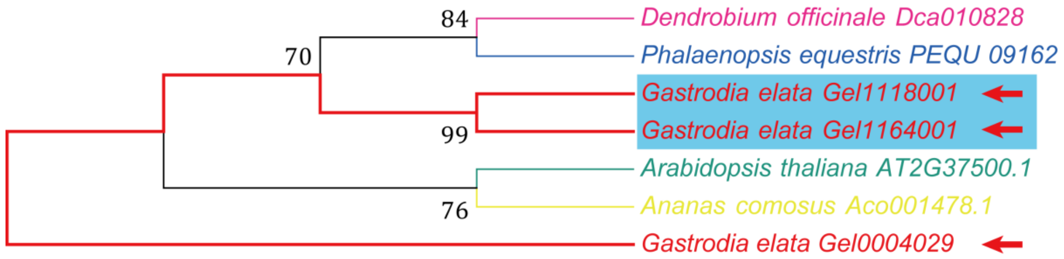

Supplementary Figure 27 | A phylogenetic neighbor-joining tree constructed from *ArgJ* involved in arginine biosynthesis of *G. elata*, *A. thaliana*, *A. comosus*, *D. officinale*, and *P. equestris*. *ArgJ* gene of *G. elata* is marked with rose lines and arrow head. *ArgJ genes* of *A. thaliana*, *A. comosus*, *D. officinale*, and *P. equestris* are respectively marked with green, yellow, pink and blue line.

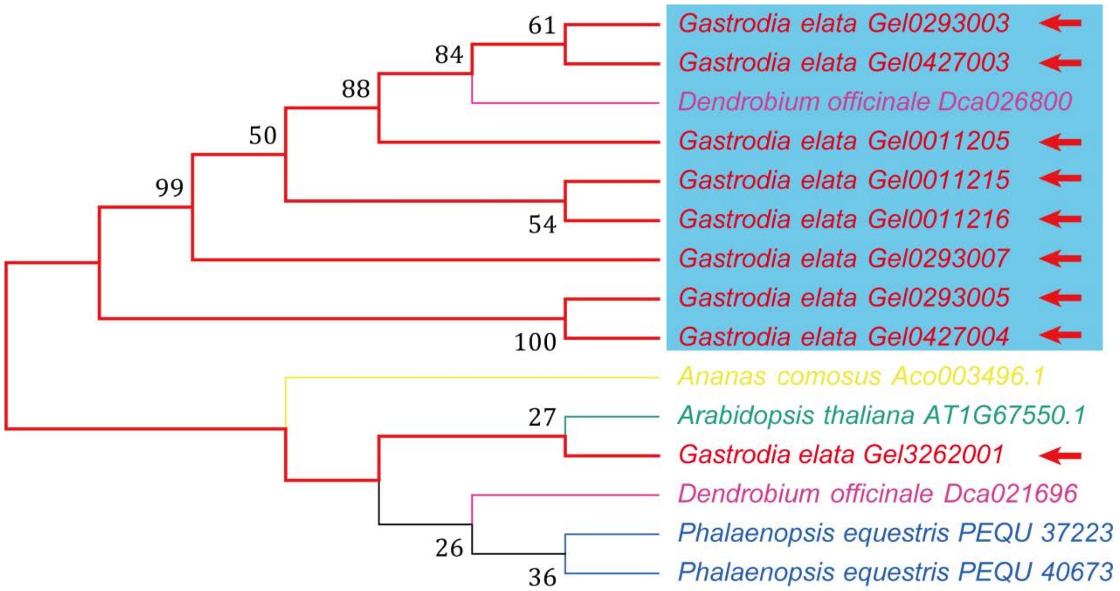

Supplementary Figure 28 | A phylogenetic neighbor-joining tree constructed from *Urease* of *G. elata*, *A. thaliana*, *A. comosus*, *D. officinale*, and *P. equestris*. *Urease* gene of *G. elata* is marked with rose lines and arrow head. *Urease genes* of *A. thaliana*, *A. comosus*, *D. officinale*, and *P. equestris* are respectively marked with green, yellow, pink and blue line.

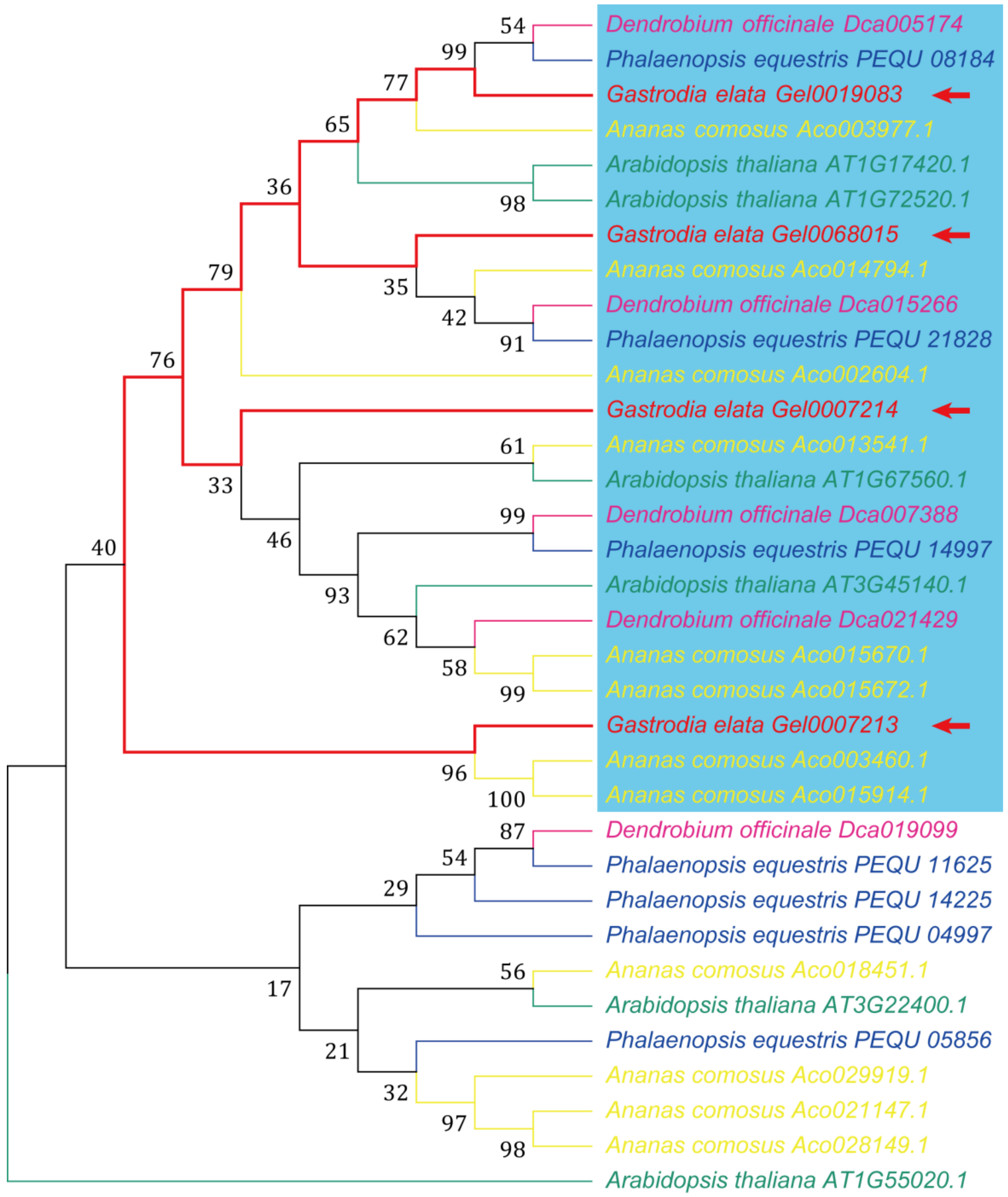

Supplementary Figure 29 | A phylogenetic neighbor-joining tree constructed from Lipoxygenase of *G. elata*, *A. thaliana*, *A. comosus*, *D. officinale*, and *P. equestris*. Lipoxygenase gene of *G. elata* is marked with rose lines and arrow head. Lipoxygenase *genes* of *A. thaliana*, *A. comosus*, *D. officinale*, and *P. equestris* are respectively marked with green, yellow, pink and blue line.

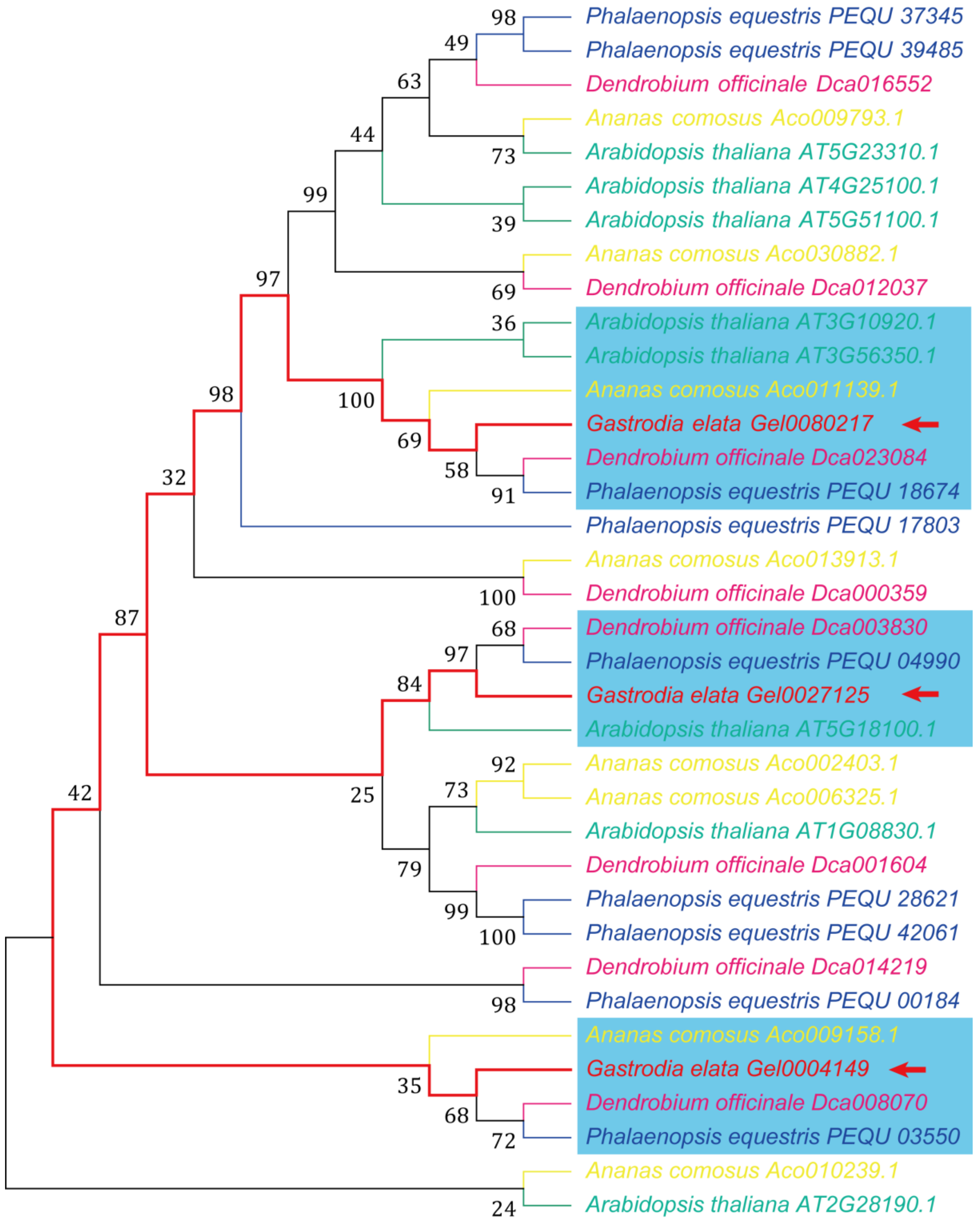

Supplementary Figure 30 | A phylogenetic neighbor-joining tree constructed from Superoxide Dismutase (SOD) of *G. elata*, *A. thaliana*, *A. comosus*, *D. officinale*, and *P. equestris*. SOD gene of *G. elata* is marked with rose lines and arrow head. SOD *genes* of *A. thaliana*, *A. comosus*, *D. officinale*, and *P. equestris* are respectively marked with green, yellow, pink and blue line.

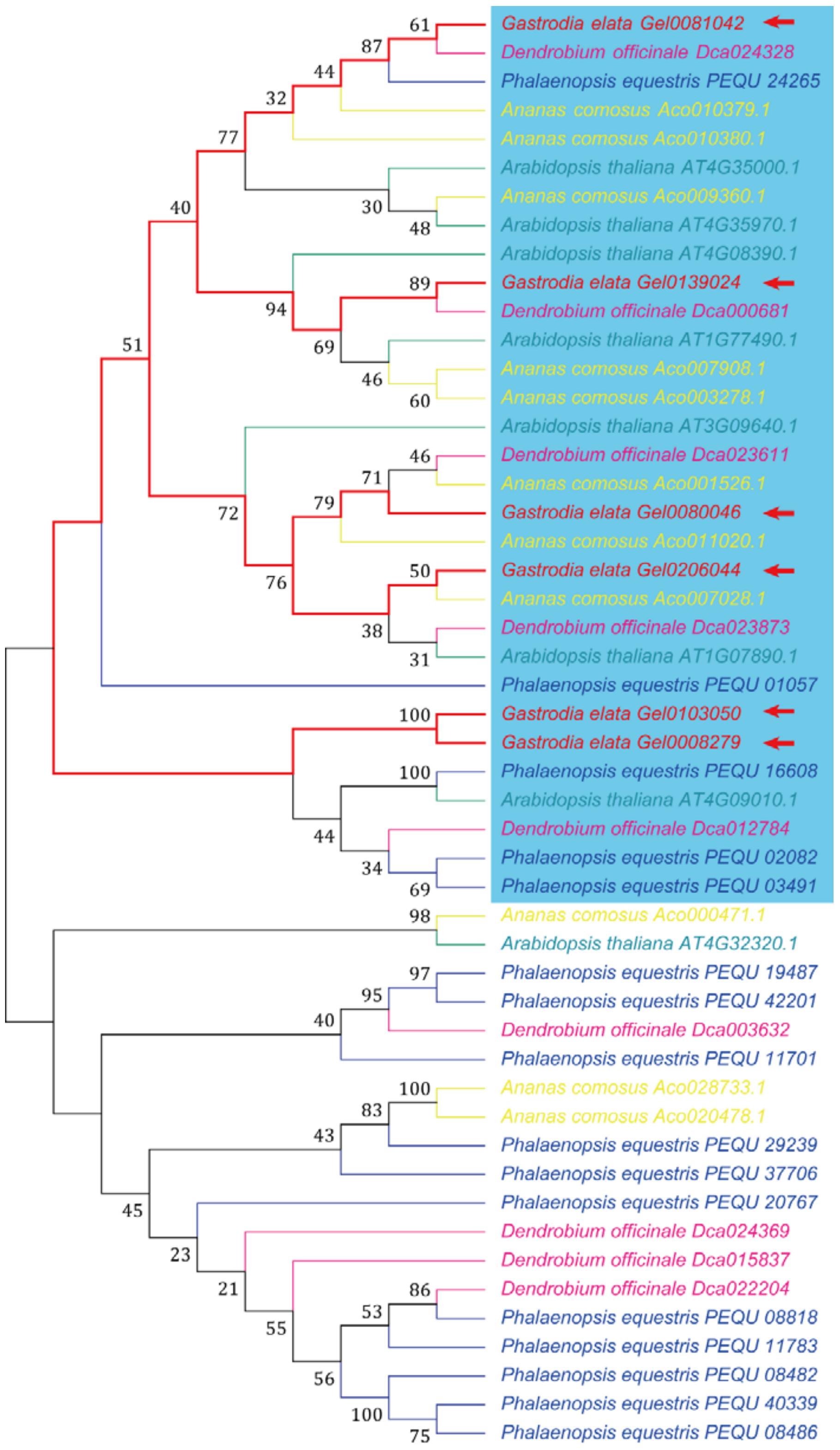

Supplementary Figure 31 | A phylogenetic neighbor-joining tree constructed from Ascorbate Peroxidase (APX) of *G. elata*, *A. thaliana*, *A. comosus*, *D. officinale*, and *P. equestris*. APX gene of *G. elata* is marked with rose lines and arrow head. APX *genes* of *A. thaliana*, *A. comosus*, *D. officinale*, and *P. equestris* are respectively marked with green, yellow, pink and blue line.

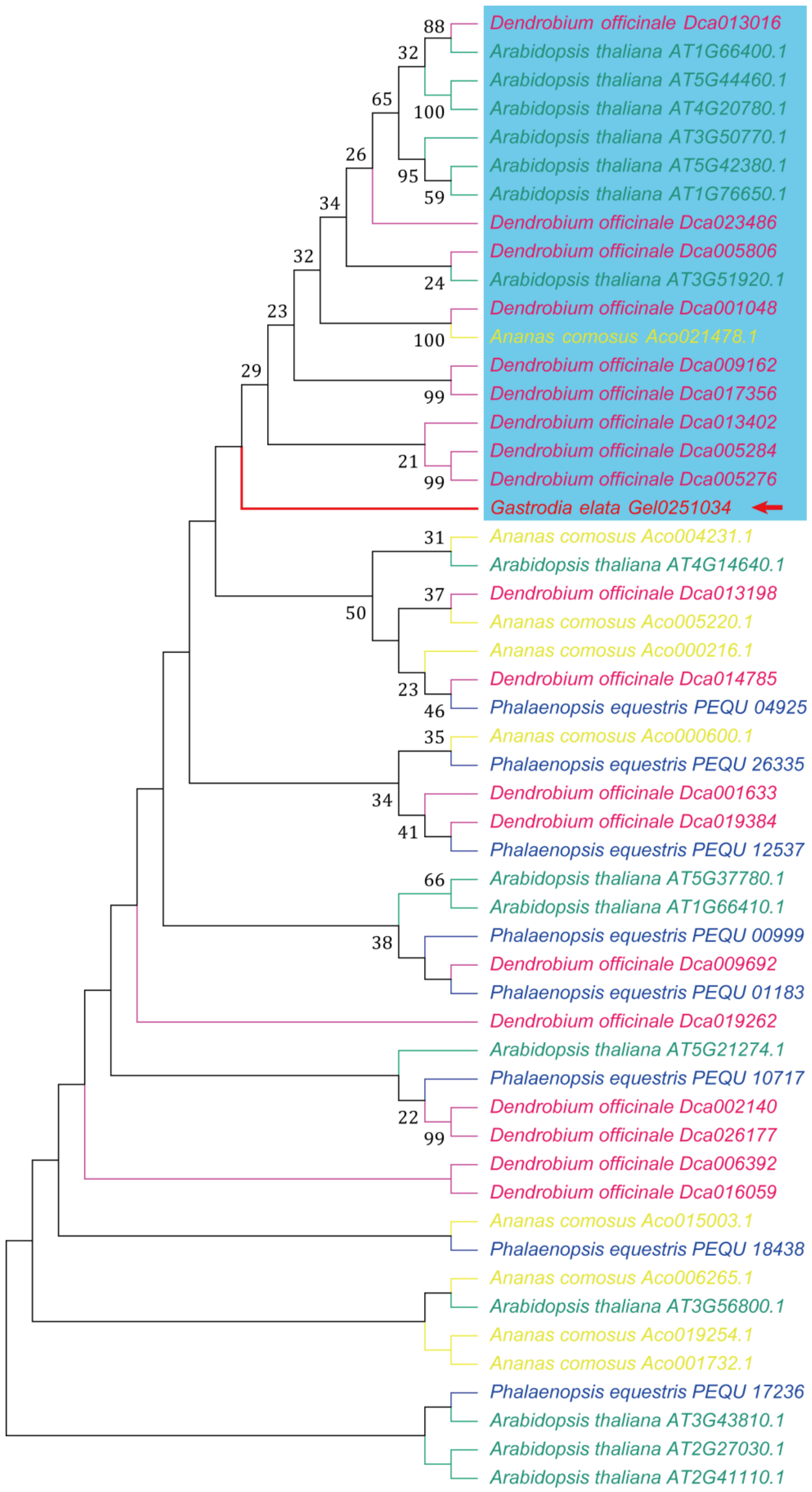

Supplementary Figure 32 | A phylogenetic neighbor-joining tree constructed from Calmodulin-1 (CAM) of *G. elata*, *A. thaliana*, *A. comosus*, *D. officinale*, and *P. equestris*. CAM gene of *G. elata* is marked with rose lines and arrow head. CAM *genes* of *A. thaliana*, *A. comosus*, *D. officinale*, and *P. equestris* are respectively marked with green, yellow, pink and blue line.

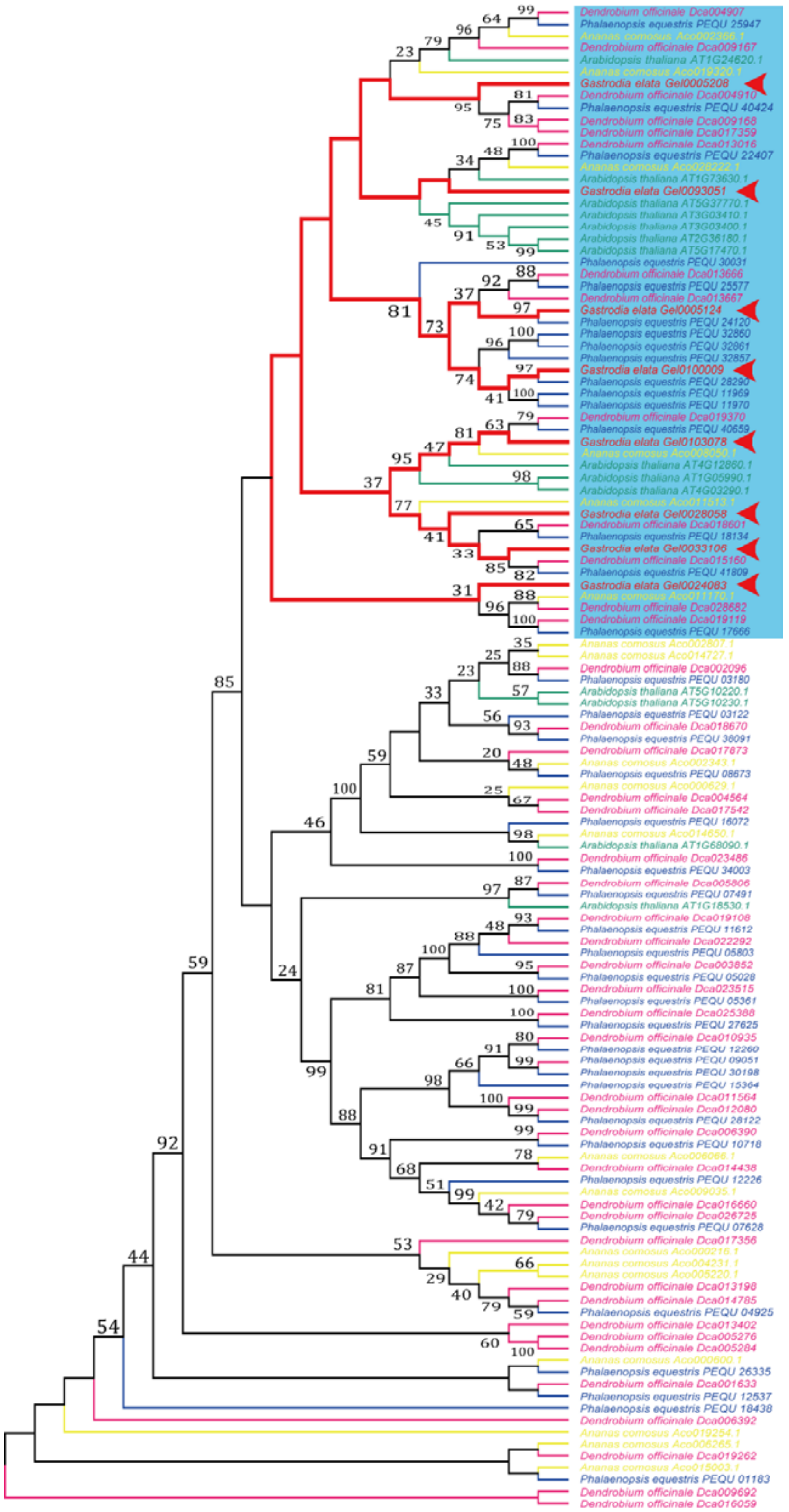

Supplementary Figure 33 | A phylogenetic neighbor-joining tree constructed from Calcium-binding protein (CML) of *G. elata*, *A. thaliana*, *A. comosus*, *D. officinale*, and *P. equestris*. CML gene of *G. elata* is marked with rose lines and arrow head. CML *genes* of *A. thaliana*, *A. comosus*, *D. officinale*, and *P. equestris* are respectively marked with green, yellow, pink and blue line.

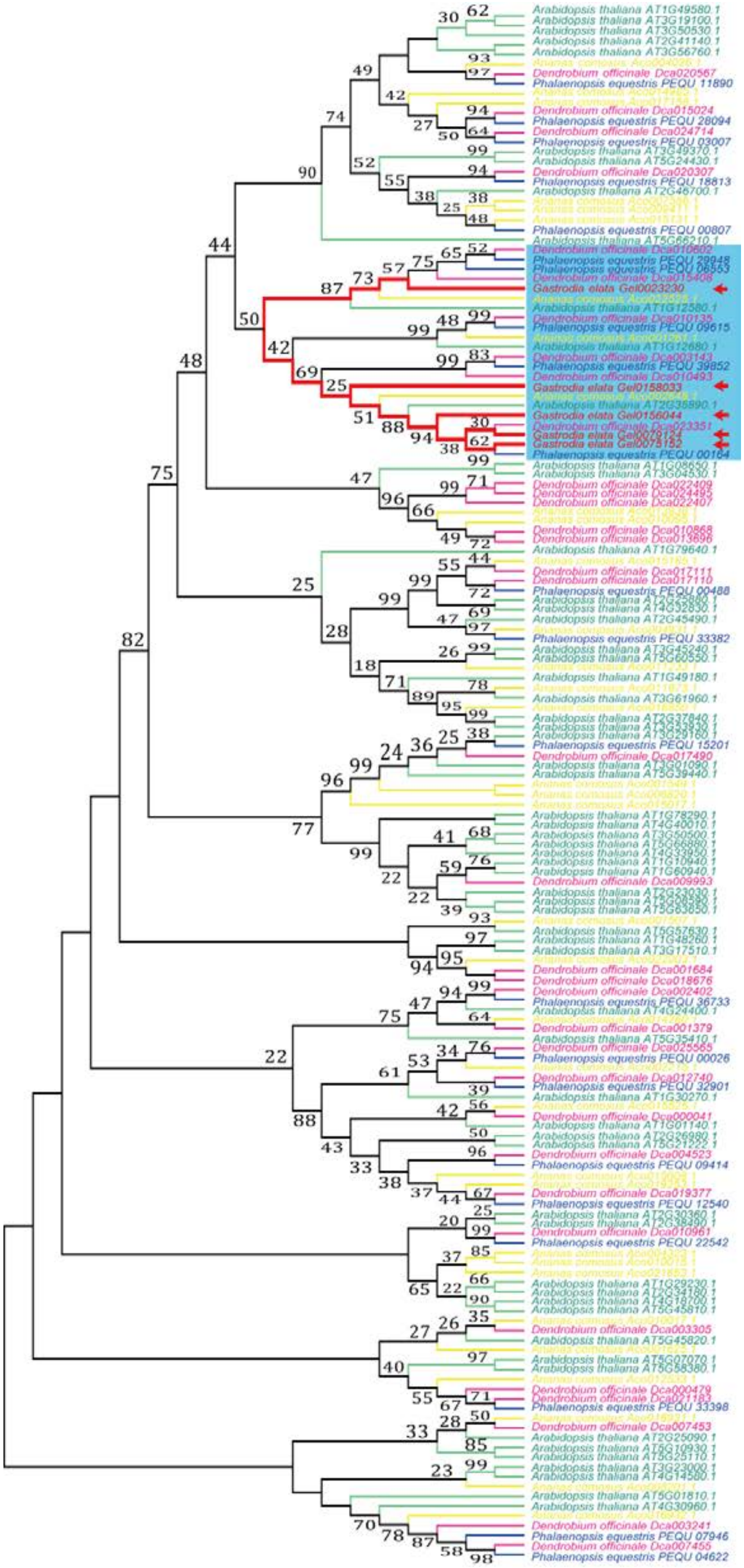

Supplementary Figure 34 | A phylogenetic neighbor-joining tree constructed from Calcium-dependent Protein Kinase (CPK) of *G. elata*, *A. thaliana*, *A. comosus*, *D. officinale*, and *P. equestris*. CPK gene of *G. elata* is marked with rose lines and arrow head. CPK *genes* of *A. thaliana*, *A. comosus*, *D. officinale*, and *P. equestris* are respectively marked with green, yellow, pink and blue line.

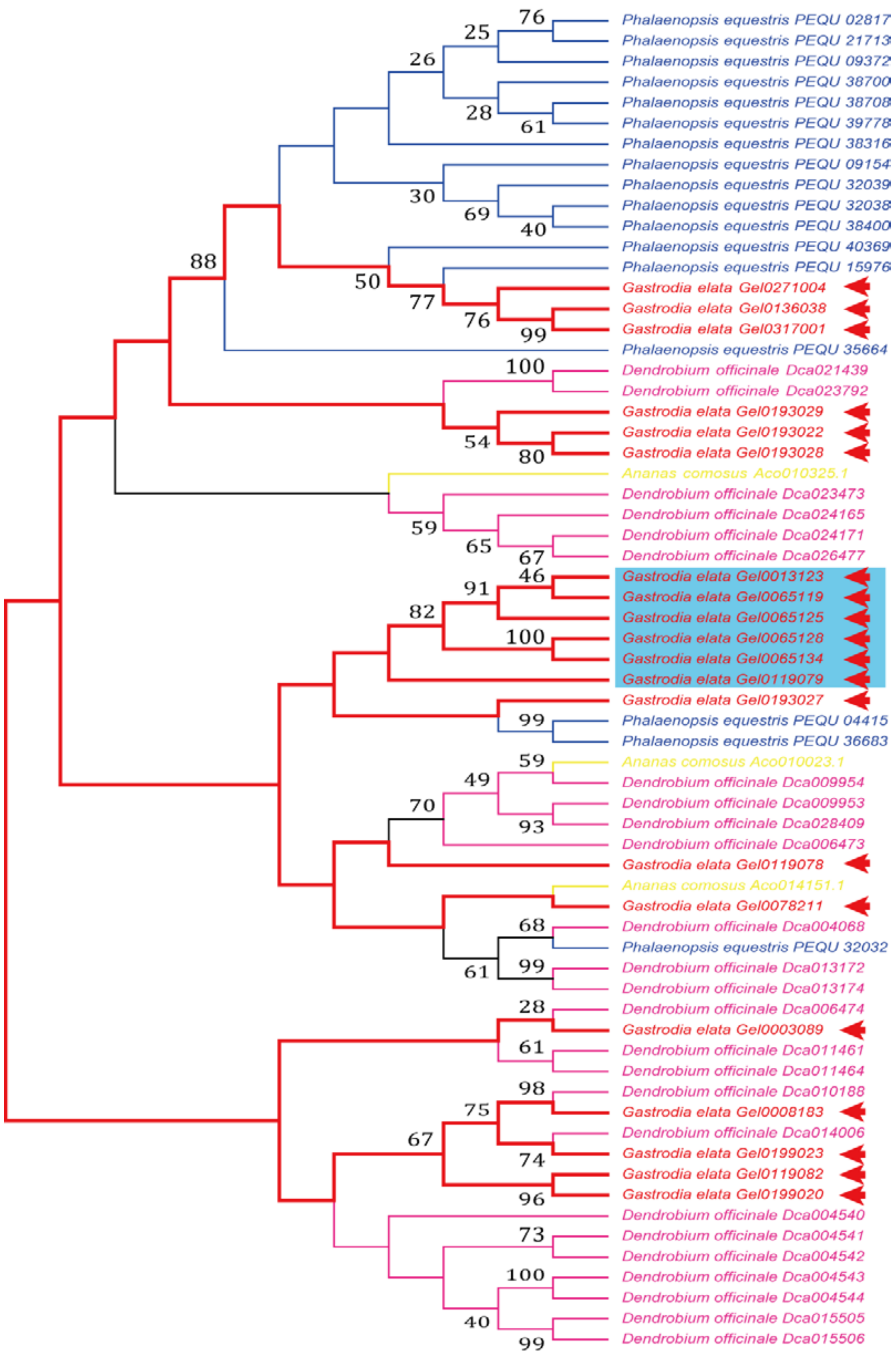

Supplementary Figure 35 | A phylogenetic neighbor-joining tree constructed from Monocot Mannose-binding Lectin Antifungal Protein (GAFP) of *G. elata*, *A. thaliana*, *A. comosus*, *D. officinale*, and *P. equestris*. GAFP gene of *G. elata* is marked with rose lines and arrow head. GAFP *genes* of *A. thaliana*, *A. comosus*, *D. officinale*, and *P. equestris* are respectively marked with green, yellow, pink and blue line.

Supplementary Tables

Supplementary Table 1 | Genome sequencing analysis. The Illumina sequencing libraries with insert size ranging from 230 bp to 20 kb. Coverage figures assume a 1.18 Gb genome size.

Platform	Insert size	Raw data (G)	Sequence coverage (X)
Illumina HiSeq 2500	230 bp	73	61.86
	350 bp	17.5	14.83
	500 bp	17.8	15.08
	2 kb	34.3	29.07
	5 kb	15.4	13.05
	10 kb	9.8	8.31
	15 kb	7.8	6.61
	20 kb	3.5	2.97
	Total	179.1	151.78

* The estimated genome size is about 1.18 Gb, and sequence coverage equals the total raw bases/1.18 Gb.

Supplementary Table 2 | Statistics related to the assembly of the *G. elata* genome. The contig N50 of *G. elata* genome assembled was 68.9 Kb and the scaffold N50 was 4.9 Mb. Only the length of scaffold greater than 100 bp was counted.

**Sample ID**	**Length**		**Number**	
	**Contig (bp)**	**Scaffold (>100 bp)**	**Contig**	**Scaffold (>100 bp)**
Total	1,025,512,599	1,061,091,640	45,804	3,779
Max	607,732	24,690,109	-	-
Number>=100	-	-	45,803	3,779
Number>=2000	-	-	29,208	2,871
N50	68,979	4,911,943	4,397	67
N60	54,429	4,323,729	6,072	90
N70	41,581	3,270,835	8,224	117
N80	28,892	2,497,872	11,172	155
N90	15,171	1,349,508	15,944	210

Note: N50 refers to the length of sequence equal to or greater than the half of total sequences length.

Supplementary Table 3 | Assessment of genome coverage rate using raw reads. Mapping rate was calculated through raw reads mapped to the *G. elata* genome to quantify the depth of genome coverage.

	**Sample ID**	**Percentage**
Reads	Mapping rate (%)	98.51%
	Average sequencing depth	61.69
	Coverage (%)	99.84%
Genome	Coverage at least 4X (%)	99.11%
	Coverage at least 10X (%)	97.25%
	Coverage at least 20X (%)	92.84%

Supplementary Table 4 | Assessment of gene coverage rate using EST data. ESTs assembled by transcriptome data, sequenced from the scape, were aligned to the scaffolds, and 94.57% ESTs were over 90% covered in one scaffold.

**Dataset**	**Number**	**Total length (bp)**	**Sequences covered by assembly (%)**	**with >90% sequence in one scaffold**		**with >50% sequence in one scaffold**	
				**Number**	**Percent**	**Number**	**Percent**
>0bp	80,646	53,498,860	98.66	76,140	94.41	79,149	98.48
>500bp	26,147	37,678,425	99.82	24,728	94.57	26,040	99.59
>1kbp	13,925	29,209,024	99.94	13,170	94.58	13,886	99.72
>2kbp	5,556	17,198,080	100	5,198	93.56	5,538	99.68

Supplementary Table 5 | Core Eukaryotic Genes Mapping Approach (CEGMA) results of *G. elata* genome. CEGMA defined the number of 248 ultra-conserved CEGs that occur in a wide range of eukaryotes. A protein is classified as complete if the alignment of the predicted protein to the HMM profile represents at least 70% of the original KOG domain, otherwise it is classified as partial.

**Species**	**Complete**		**Complete + partial**	
	**Prots**	**% completeness**	**Prots**	**% completeness**
G. elata	217	87.5	239	96.37
P. equestris	186	75	229	92.34
D. officinale	187	75.4	222	89.52

Supplementary Table 6 | Categories of TEs predicted in the *G. elata* genome. The genomic abundance of TEs were predicted by RepeatModeler and RepeatMasker through an ab-initio method and homology based method. The TEs of Denovo+Repbase were predicted based on the TEs libraries predicted RepeatModeler and Repbase. The TEs of TE proteins were predicted based on Repbased. The final results of TEs in *G. elata* genome were integrated of both methods without redundancy.

		**Denovo+Repbase**		**TE Proteins**		**Combined TEs**	
		**Length (bp)**	**% in Genome**	**Length (bp)**	**% in Genome**	**Length (bp)**	**% in Genome**
DNA transposon	DNA	45,215,332	4.26	2,971,001	0.28	46,521,569	4.38
	LINE	25,759,088	2.43	20,738,011	1.95	36,614,112	3.45
Retrotransposon	SINE	1,118	0	0	0	1,118	0
	LTR	573,095,875	54.01	135,423,895	12.76	593,527,755	55.94
	Other	68	0	0	0	68	0
	Satellite	0	0	0	0	0	0
Other	Simple repeat	858,911	0.08	0	0	858,911	0.08
	Unknown	70,501,667	6.64	0	0	70,501,667	6.64
	Total	680,001,727	64.09	158,915,266	14.98	702,250,876	66.18

Supplementary Table 7 | Summary of transposable elements (TEs) and other repeats in the assembly of *Gastrodia elata*.

**TE category[2]**	**Total length (bp)**	**Percentage (%)**
LTR/Gypsy	477,875,665	45.04
LTR/Copia	75,311,650	7.10
LTR/LTR	67,561,718	6.37
LTR/ERV1	1,482,733	0.14
LTR/ERV4	1,293,176	0.12
LINE/L1	21,497,264	2.03
LINE/RTE	13,149,489	1.24
LINE/Penelope	960,965	0.09
DNA/CMC	27,808,296	2.62
DNA/hAT	4,187,470	0.39
DNA/DNA	4,020,143	0.38
DNA/MULE	3,354,733	0.32

Supplementary Table 8 | Copy number of LTR families.

Super family	family[2]	LTR number		
		Gastrodia elata	***Phalaenopsis equestris***	***Dendrobium catenatum***
Copia	oryco	106	303	127
	retrofit	873	1187	1,312
	sire	125	134	1,861
	tork	1,720	2,057	63
	copia	45	198	23
Gypsy	del	6,604	393	375
	athila	2,431	5,325	29
	crm	821	844	944
	reina	16	291	609
	tat	31	1926	422

Supplementary Table 9 | General statistics of mapping rate to functional database of protein-coding genes. Gene function were obtained from the best BLASTp hit in SwissProt and TrEMBL databases. Gene domain annotation was carried out by searching the InterPro database. All genes were aligned against KEGG and GO terms to obtain a functional annotation.

	Gastrodia elata		***Dendrobium officinale***		***Phalaenopsis equestris***	
	Number	Percent (%)	Number	Percent (%)	Number	Percent (%)
Total	18,969	-	28,910	-	29,431	-
InterPro	12,720	67.06	18,655	64.53	18,305	62.20
KEGG	15,089	79.55	13,656	47.24	18,350	62.35
Swissprot	11,444	60.33	15,697	54.30	16,966	57.65
TrEMBL	12,538	66.10	23,978	82.94	23,227	78.92
GO	9,356	49.32	13,414	46.40	14,163	48.12
Annotated	15,479	81.60	24,059	83.22	23,611	80.22
Unannotated	3,490	18.40	4,851	16.78	5,820	19.78

Supplementary Table 10 | Number of genes expressed during five growth stages of *G. elata*. (A) protocorms, (B) juvenile tubers, (C) immature tubers, (D) mature tubers, and (E) flowering scapes.

	**A**	**B**	**C**	**D**	**E**	**A-E**[*]
Number (RPKM[†] >= 1)	15748	15992	15071	15086	15115	16823
Percentage	83.02	84.31	79.45	79.53	79.68	88.69

* Gene at least in one tissue. † RPKM: reads per kilobase per million mapped reads.

Supplementary Table 11 | GO category enrichment analysis of the five co-expression modules (C1-C5) during five development stages of *G. elata*. The first row lists the module numbers (from cluster1 to cluster 5). GO categories that were significant enriched for each cluster were shown (Fisher's exact test, with a false discovery rate < 0.01) (MF: molecular function; BP: biological process; CC: cellular component).

GO ID	GO Term	GO Class	Cluster 1	Cluster 2	Cluster 3	Cluster 4	Cluster 5
GO:0006334	nucleosome assembly	BP	0.000936894	0.006785007	0	0	0
GO:0044699	single-organism process	BP	1.59E-13	0.000133303	0	0	0
GO:0055085	transmembrane transport	BP	0.000138156	0.005692885	0	0	0
GO:0055114	oxidation-reduction process	BP	4.38E-12	0.001168483	0	0	0
GO:0065004	protein-DNA complex assembly	BP	0.001302193	0.006664705	0	0	0
GO:0006259	DNA metabolic process	BP	0.000492194	0	0	0	0
GO:0006268	DNA unwinding involved in DNA replication	BP	0.007588686	0	0	0	0
GO:0006323	DNA packaging	BP	0.000229306	0	0	0	0
GO:0006721	terpenoid metabolic process	BP	0.002418803	0	0	0	0
GO:0006855	drug transmembrane transport	BP	3.18E-08	0	0	0	0
GO:0006979	response to oxidative stress	BP	0.005138504	0	0	0	0
GO:0007169	transmembrane receptor protein tyrosine kinase signaling pathway	BP	0.001302193	0	0	0	0
GO:0008152	metabolic process	BP	9.55E-08	0	0	0	0
GO:0009751	response to salicylic acid	BP	2.41E-05	0	0	0	0
GO:0009886	post-embryonic morphogenesis	BP	0.009026426	0	0	0	0
GO:0009887	organ morphogenesis	BP	0.001353268	0	0	0	0
GO:0010583	response to cyclopentenone	BP	0.000604699	0	0	0	0
GO:0014070	response to organic cyclic compound	BP	1.59E-06	0	0	0	0
GO:0016114	terpenoid biosynthetic process	BP	0.002867773	0	0	0	0

GO:0031497	chromatin assembly	BP	0.000754174	0	0	0	0
GO:0032776	DNA methylation on cytosine	BP	0.006167519	0	0	0	0
GO:0042221	response to chemical	BP	0.002316133	0	0	0	0
GO:0044710	single-organism metabolic process	BP	2.29E-08	0	0	0	0
GO:0044763	single-organism cellular process	BP	0.001990924	0	0	0	0
GO:0050896	response to stimulus	BP	4.28E-05	0	0	0	0
GO:0071103	DNA conformation change	BP	1.77E-06	0	0	0	0
GO:0006970	response to osmotic stress	BP	0	0.000427772	0	0	0
GO:0009651	response to salt stress	BP	0	0.001053555	0	0	0
GO:0010103	stomatal complex morphogenesis	BP	0	0.007277409	0	0	0
GO:0042775	mitochondrial ATP synthesis coupled electron transport	BP	0	0.005692885	0	0	0
GO:0043086	negative regulation of catalytic activity	BP	0	0.000315196	0	0	0
GO:0045333	cellular respiration	BP	0	0.001128031	0	0	0
GO:0050790	regulation of catalytic activity	BP	0	0.002356055	0	0	0
GO:0065009	regulation of molecular function	BP	0	0.001308455	0	0	0
GO:0000377	RNA splicing, via transesterification reactions with bulged adenosine as nucleophile	BP	0	0	0.007555666	0	0
GO:0000398	mRNA splicing, via spliceosome	BP	0	0	0.007089317	0	0
GO:0006139	nucleobase-containing compound metabolic process	BP	0	0	0.00031265	0	0
GO:0006260	DNA replication	BP	0	0	0.004179703	0	0
GO:0006364	rRNA processing	BP	0	0	0.008209067	0	0
GO:0006396	RNA processing	BP	0	0	1.48E-11	0	0

GO:0006397	mRNA processing	BP	0	0	0.004070251	0	0
GO:0006412	translation	BP	0	0	5.43E-11	0	0
GO:0006457	protein folding	BP	0	0	0.00210215	0	0
GO:0006626	protein targeting to mitochondrion	BP	0	0	0.001583624	0	0
GO:0006725	cellular aromatic compound metabolic process	BP	0	0	0.008497932	0	0
GO:0006807	nitrogen compound metabolic process	BP	0	0	1.25E-08	0	0
GO:0007005	mitochondrion organization	BP	0	0	0.004239399	0	0
GO:0008380	RNA splicing	BP	0	0	6.50E-05	0	0
GO:0009059	macromolecule biosynthetic process	BP	0	0	0.004070251	0	0
GO:0009451	RNA modification	BP	0	0	0.001583624	0	0
GO:0009793	embryo development ending in seed dormancy	BP	0	0	0.003962529	0	0
GO:0010467	gene expression	BP	0	0	2.10E-10	0	0
GO:0016070	RNA metabolic process	BP	0	0	0.002428431	0	0
GO:0016071	mRNA metabolic process	BP	0	0	0.000614625	0	0
GO:0034641	cellular nitrogen compound metabolic process	BP	0	0	2.19E-08	0	0
GO:0034645	cellular macromolecule biosynthetic process	BP	0	0	0.004064449	0	0
GO:0042254	ribosome biogenesis	BP	0	0	6.59E-11	0	0
GO:0043043	peptide biosynthetic process	BP	0	0	5.43E-11	0	0
GO:0043170	macromolecule metabolic process	BP	0	0	0.000100825	0	0
GO:0043604	amide biosynthetic process	BP	0	0	1.66E-10	0	0
GO:0044085	cellular component biogenesis	BP	0	0	2.49E-06	0	0

GO:0044260	cellular macromolecule metabolic process	BP	0	0	5.91E-06	0	0
GO:0046483	heterocycle metabolic process	BP	0	0	0.00308187	0	0
GO:0071840	cellular component organization or biogenesis	BP	0	0	0.001583624	0	0
GO:0090304	nucleic acid metabolic process	BP	0	0	1.28E-05	0	0
GO:1901564	organonitrogen compound metabolic process	BP	0	0	0.009712328	0	0
GO:1901566	organonitrogen compound biosynthetic process	BP	0	0	1.38E-06	0	0
GO:0009266	response to temperature stimulus	BP	0	0	0	0.005269076	0
GO:0009408	response to heat	BP	0	0	0	0.002237174	0
GO:0010228	vegetative to reproductive phase transition of meristem	BP	0	0	0	0.001152215	0
GO:0006470	protein dephosphorylation	BP	0	0	0	0	0.001835364
GO:0006865	amino acid transport	BP	0	0	0	0	0.000487672
GO:0006914	autophagy	BP	0	0	0	0	0.000462466
GO:0009698	phenylpropanoid metabolic process	BP	0	0	0	0	0.003052209
GO:0009834	secondary cell wall biogenesis	BP	0	0	0	0	7.52E-05
GO:0010417	glucuronoxylan biosynthetic process	BP	0	0	0	0	0.009989018
GO:0016311	dephosphorylation	BP	0	0	0	0	0.000656039
GO:0019748	secondary metabolic process	BP	0	0	0	0	0.003700579
GO:0042538	hyperosmotic salinity response	BP	0	0	0	0	1.11E-07
GO:0045491	xylan metabolic process	BP	0	0	0	0	0.009833155
GO:0046942	carboxylic acid transport	BP	0	0	0	0	0.009586478

Supplementary Table 12 | Estimated gene number in reported angiosperm genomes compared to *Gastrodia elata*.

Species	Gene number	Reference
Oryza sativa	46,022 to 55,615	3
Brachypodium distachyon	25,532	4
Setaria italica	29,000	5
Zea mays	32,540	6
Hordeum vulgare	26,159	7
Triticum aestivum	124,201	8
Aegilops tauschii	34,498	9
Sorghum bicolor	34,496	10
Ananas comosus	27,024	11
Elaeis guineensis	34,802	12
Phoenix dactylifera	25,059	13
Musa acuminata	36,542	14
Zostera marina	20,450	15
Spirodela polyrhiza	19,623	16
Phalaenopsis equestris	29,431	17
Vitis vinifera	30,434	18
Eucalyptus grandis	36,376	19
Carica papaya	24,746	20
Arabidopsis thaliana	25,498	21
Arabidopsis lyrata	32,670	22
Eutrema salsugineum	28,457	23
Brassica rapa	41,174	24
Brassica oleracea	45,758	25
Brassica napus	101,040	26
Capsella rubella	26,521	27
Raphanus sativus	61,572	28
Theobroma cacao	28,798	29
Gossypium raimondii	40,976	30
Citrus sinensis	29,445	31
Cucumis sativus	26,682	32
Citrullus lanatus	23,440	33
Cucumis melo	27,427	34
Glycine max	46,430	35
Medicago truncatula	62,388	36
Cicer arietinum	28,269	37
Vigna angularis	26,857	38
Trifolium pratense	40,868	39
Phaseolus vulgaris	26,279	40
Glycyrrhiza uralensis	34,445	41

Arachis duranensis	50,324	42
Prunus persica	27,852	43
Malus domestica	57,386	44
Pyrus bretschneideri	42,812	45
Fragaria vesca	34,809	46
Prunus mume	31,390	47
Morus notabilis	29,338	48
Ziziphus jujuba	27,443	49
Ricinus communis	31,237	50
Jatropha curcas	40,929	51
Manihot esculenta	30,666	52
Linum usitatissimum	43,384	53
Populus trichocarpa	45,555	54
Juglans regia	32,496	55
Actinidia chinensis	39,040	56
Sesamum indicum	27,148	57
Dorcoceras hygrometricum	49,374	58
Solanum tuberosum	39,031	59
Solanum lycopersicum	34,727	60
Capsicum annuum	34,903	61
Petunia inflata	36,697	62
Petunia axillaris	32,928	62
Ipomoea nil	42,783	63
Coffea canephora	25,574	64
Daucus carota	32,113	65
Chenopodium quinoa	44,776	66
Beta vulgaris	27,421	67
Nelumbo nucifera	36,385	68
Amborella trichopoda	27,313	69
Gastrodia elata	18,969	This paper

Supplementary Table 13 | Comparison of gene number in second level GO terms between three Orchid genomes. The table depicts the comparison of gene numbers of second level GO terms in *G. elata* versus *D. catenatum* and *P. equestris* (Fisher's exact test, $p < 0.05$, adjusted by false discovery rate) (MF: molecular function; BP: biological process; CC: cellular component).

**GO ID**	**GO Term**	**GO Class**	**Number of Genes**			**Adjusted**
			D. catenatum	*P. equestris*	*G. elata*	***p*-value**
**GO:0005623**	cell	CC	12836	12634	8748	3.41788E-09
**GO:0044464**	cell part	CC	12836	12634	8748	3.41788E-09
**GO:0043226**	organelle	CC	7988	7998	5538	7.01714E-05
**GO:0071840**	cellular component organization or biogenesis	BP	1697	1614	1218	0.004807157
**GO:0031974**	membrane-enclosed lumen	CC	553	579	447	0.008734457
**GO:0032991**	macromolecular complex	CC	1941	2084	1444	0.0184643
**GO:0000003**	reproduction	BP	1360	1246	954	0.02080694
**GO:0016020**	membrane	CC	4942	4942	3406	0.02156278
**GO:0022414**	reproductive process	BP	1358	1244	951	0.02156278
**GO:0032501**	multicellular organismal process	BP	2382	2152	1592	0.06573695
**GO:0032502**	developmental process	BP	2581	2379	1734	0.06839851
**GO:0050896**	response to stimulus	BP	4673	4669	2938	0.1050429
**GO:0051179**	localization	BP	1955	1940	1366	0.1050429
**GO:0048519**	negative regulation of biological process	BP	341	320	255	0.1061546
**GO:0065007**	biological regulation	BP	3329	3290	2270	0.1493996
**GO:0009295**	nucleoid	CC	60	47	22	0.1734055
**GO:0002376**	immune system process	BP	325	256	162	0.2055717
**GO:0003824**	catalytic activity	MF	8782	8721	5620	0.2055717
**GO:0008152**	metabolic process	BP	9935	9915	6387	0.2055717
**GO:0022610**	biological adhesion	BP	23	18	21	0.2820339

**GO:0005198**	structural molecule activity	MF	478	534	365	0.2914804
**GO:0050789**	regulation of biological process	BP	2998	2993	2036	0.3073398
**GO:0005488**	binding	MF	11678	10706	7243	0.3499489
**GO:0023052**	signaling	BP	1024	1013	707	0.3968134
**GO:0044425**	membrane part	CC	1564	1656	1103	0.3968134
**GO:0045735**	nutrient reservoir activity	MF	34	31	14	0.3968134
**GO:0005085**	guanyl-nucleotide exchange factor activity	MF	22	20	19	0.4570529
**GO:0040007**	growth	BP	424	395	291	0.4570529
**GO:0005215**	transporter activity	MF	1252	1246	856	0.4589171
**GO:0009987**	cellular process	BP	9889	9286	6354	0.5328943
**GO:0030054**	cell junction	CC	19	15	15	0.5634897
**GO:0000988**	protein binding transcription factor activity	MF	51	53	41	0.5646206
**GO:0016209**	antioxidant activity	MF	103	127	67	0.6848928
**GO:0009055**	electron carrier activity	MF	439	460	284	0.8020843
**GO:0016247**	channel regulator activity	MF	1	1	1	0.8020843
**GO:0031012**	extracellular matrix	CC	14	16	7	0.8020843
**GO:0040011**	locomotion	BP	18	13	12	0.8020843
**GO:0044422**	organelle part	CC	2781	2937	1899	0.8020843
**GO:0045182**	translation regulator activity	MF	7	2	4	0.8020843
**GO:0048518**	positive regulation of biological process	BP	308	292	205	0.8020843
**GO:0055044**	symplast	CC	14	11	10	0.8020843
**GO:0051704**	multi-organism process	BP	405	348	255	0.8460895
**GO:0001906**	cell killing	BP	3	3	3	0.8473848
**GO:0016530**	metallochaperone activity	MF	2	3	2	0.8473848
**GO:0044848**	biological phase	BP	38	28	19	0.8473848
**GO:0044699**	single-organism process	BP	7294	7595	4903	0.847834

**GO:0048511**	rhythmic process	BP	78	91	53	0.9368516
**GO:0044421**	extracellular region part	CC	17	21	13	0.9805771
**GO:0001071**	nucleic acid binding transcription factor activity	MF	1517	1486	985	1
**GO:0004872**	receptor activity	MF	143	120	85	1
**GO:0005576**	extracellular region	CC	376	434	263	1
**GO:0030234**	enzyme regulator activity	MF	180	167	114	1
**GO:0031386**	protein tag	MF	8	10	5	1
**GO:0060089**	molecular transducer activity	MF	143	120	85	1

Supplementary Table 14 | Under-represented Pfam domains in *G. elata* genome. The table depicts the comparison of percentages and absolute numbers of respective under-represented domains in *G. elata* (Fisher's exact test, $p<0.01$, adjusted by false discovery rate).

Pfam ID: Pfam Name	Gel[*]	Peq[†]	Dof[‡]	Ath[§]	Vvi[‖]	Aco[¶]	Osa[#]	Adjusted p-value
PF00931:NB-ARC	5	64	138	167	316	169	498	4.17E-47
PF00646:F-box	36	47	71	520	71	78	340	3.11E-14
PF07727:RVT_2	0	139	170	4	3	2	4	2.11E-11
PF12776:Myb_DNA-bind_3	1	209	12	14	17	40	28	5.04E-10
PF00078:RVT_1	5	20	300	8	6	10	27	4.46E-08
PF13855:LRR_8	95	133	163	254	400	243	405	1.86E-06
PF13456:RVT_3	2	24	73	54	4	9	46	7.06E-05
PF13968:DUF4220	0	1	13	6	18	10	106	7.5E-05
PF10536:PMD	2	16	37	15	10	94	33	0.000136
PF04578:DUF594	0	1	10	6	16	12	100	0.00023
PF08268:FBA_3	0	0	3	110	3	3	26	0.00023
PF03140:DUF247	2	9	19	26	16	50	74	0.00023
PF00504:Chloroa_b-bind	0	17	23	28	23	24	22	0.00045
PF13966:zf-RVT	1	7	67	21	7	41	14	0.000643
PF13962:PGG	7	6	13	42	123	14	74	0.000805
PF00560:LRR_1	122	171	195	285	441	242	394	0.000942
PF13947:GUB_WAK_bind	8	8	19	33	39	35	124	0.006734
PF13516:LRR_6	40	73	80	98	153	117	168	0.009305
PF00139:Lectin_legB	8	9	20	54	39	28	97	0.016355

* Gel: *G. elata*; † Peq: *P. equestris*; ‡ Dof: *D. officinale*; § Ath: *A. thaliana*; ‖ Vvi: *V. vinifera*; ¶ Aco: *A. comosus*; # Osa: *O. sativa*.

Supplementary Table 15 | Comparison of the estimated number of gene families of *G. elata* with other plants.

Species	Total no. of genes [*]	No. of unclustered genes	Clustered genes	Families	Unique families	No. of genes per family
G. elata	17,999	4,324	13,675	10,487	251	1.30
P. equestris	29,316	7,862	21,454	13,358	630	1.61
D. officinale	28,879	6,729	22,150	13,475	614	1.64
O. sativa	39,004	11,486	27,518	15,236	1,481	1.81
Z. mays	33,266	7,038	26,228	14,176	1,251	1.85
A. comosus	26,918	5,094	21,824	12,719	724	1.72
E. guineensis	25,342	2,283	23,059	12,372	298	1.86
A. thaliana	26,930	3,451	23,479	12,694	727	1.85
P. trichocarpa	39,981	7,513	32,468	14,279	997	2.27
V. vinifera	28,227	5,894	22,333	13,393	684	1.67
G. max	54,864	9,097	45,767	14,731	1,684	3.11
Pinus abies	25,760	4,920	20,840	8,872	1,378	2.35
Physcomitrella patens	31,502	11,868	19,634	8,878	1,376	2.21
Amborella trichopoda	26,797	7,676	19,121	12,237	995	1.56

Supplementary Table 16 | GO term enrichment of *G. elata* contracted gene families.

GO ID	GO Term	GO Class†	Adjusted p-value*
GO:0004713	protein tyrosine kinase activity	MF	7.21E-126
GO:0004672	protein kinase activity	MF	9.20E-103
GO:0006468	protein phosphorylation	BP	4.01E-102
GO:0016310	phosphorylation	BP	3.79E-86
GO:0016773	phosphotransferase activity, alcohol group as acceptor	MF	3.07E-79
GO:0020037	heme binding	MF	3.36E-78
GO:0016301	kinase activity	MF	3.22E-73
GO:0004497	monooxygenase activity	MF	1.24E-72
GO:0019825	oxygen binding	MF	4.23E-58
GO:1901363	heterocyclic compound binding	MF	7.58E-56
GO:0097159	organic cyclic compound binding	MF	2.06E-55
GO:0035639	purine ribonucleoside triphosphate binding	MF	6.03E-54
GO:0005524	ATP binding	MF	9.22E-54
GO:0032555	purine ribonucleotide binding	MF	2.36E-53
GO:0032550	purine ribonucleoside binding	MF	2.36E-53
GO:0004674	protein serine/threonine kinase activity	MF	2.11E-52
GO:0008152	metabolic process	BP	4.43E-52
GO:0003824	catalytic activity	MF	2.17E-50
GO:0097367	carbohydrate derivative binding	MF	2.47E-50
GO:0016705	oxidoreductase activity, acting on paired donors, with incorporation or reduction of molecular oxygen	MF	2.52E-50
GO:0016772	transferase activity, transferring phosphorus-containing groups	MF	3.54E-50
GO:0006796	phosphate-containing compound metabolic process	BP	2.23E-47
GO:0006793	phosphorus metabolic process	BP	2.73E-46
GO:0003700	sequence-specific DNA binding transcription factor activity	MF	2.55E-45
GO:0043168	anion binding	MF	4.08E-44
GO:0005506	iron ion binding	MF	2.23E-43
GO:0043167	ion binding	MF	1.55E-41
GO:0005488	binding	MF	4.54E-40
GO:0006355	regulation of transcription, DNA-templated	BP	4.58E-40
GO:0019222	regulation of metabolic process	BP	7.64E-38
GO:0046527	glucosyltransferase activity	MF	1.99E-37
GO:0031326	regulation of cellular biosynthetic process	BP	8.75E-37
GO:0035251	UDP-glucosyltransferase activity	MF	1.32E-36
GO:2000112	regulation of cellular macromolecule biosynthetic process	BP	2.60E-36
GO:0009889	regulation of biosynthetic process	BP	5.67E-36
GO:0009055	electron carrier activity	MF	3.13E-35
GO:0043086	negative regulation of catalytic activity	BP	2.39E-33

GO:0010033	response to organic substance	BP	2.68E-33
GO:0051171	regulation of nitrogen compound metabolic process	BP	7.44E-33
GO:0006351	transcription, DNA-templated	BP	8.94E-33
GO:0006857	oligopeptide transport	BP	1.70E-32
GO:0016740	transferase activity	MF	4.08E-32
GO:0044092	negative regulation of molecular function	BP	1.10E-31
GO:0031323	regulation of cellular metabolic process	BP	2.59E-31
GO:0050896	response to stimulus	BP	4.97E-31
GO:0050789	regulation of biological process	BP	2.23E-30
GO:0065007	biological regulation	BP	4.34E-30
GO:0006464	cellular protein modification process	BP	2.47E-29
GO:0080090	regulation of primary metabolic process	BP	5.04E-29
GO:0050794	regulation of cellular process	BP	5.84E-29
GO:0003682	chromatin binding	MF	2.25E-28
GO:0010468	regulation of gene expression	BP	4.51E-28
GO:0043412	macromolecule modification	BP	4.89E-27
GO:0030312	external encapsulating structure	CC	9.15E-27
GO:0014070	response to organic cyclic compound	BP	1.43E-26
GO:0036094	small molecule binding	MF	9.82E-26
GO:0000166	nucleotide binding	MF	3.34E-25
GO:0005618	cell wall	CC	5.17E-25
GO:0060255	regulation of macromolecule metabolic process	BP	1.08E-24
GO:0004601	peroxidase activity	MF	1.08E-24
GO:0042221	response to chemical	BP	1.48E-24
GO:0043170	macromolecule metabolic process	BP	1.66E-24
GO:0016758	transferase activity, transferring hexosyl groups	MF	2.05E-24
GO:0044238	primary metabolic process	BP	3.98E-24
GO:0008194	UDP-glycosyltransferase activity	MF	2.04E-22
GO:0071704	organic substance metabolic process	BP	4.20E-22
GO:0048046	apoplast	CC	1.76E-21
GO:0009719	response to endogenous stimulus	BP	2.68E-21
GO:0034654	nucleobase-containing compound biosynthetic process	BP	5.96E-20
GO:0010243	response to organonitrogen compound	BP	9.81E-20
GO:0055114	oxidation-reduction process	BP	1.87E-19
GO:0010200	response to chitin	BP	4.58E-19
GO:1901700	response to oxygen-containing compound	BP	5.41E-19
GO:0004553	hydrolase activity, hydrolyzing O-glycosyl compounds	MF	6.76E-19
GO:0030599	pectinesterase activity	MF	8.69E-19
GO:0019438	aromatic compound biosynthetic process	BP	1.11E-18
GO:0071944	cell periphery	CC	1.84E-17
GO:0008236	serine-type peptidase activity	MF	1.05E-16
GO:0016757	transferase activity, transferring glycosyl groups	MF	1.32E-16
GO:0051273	beta-glucan metabolic process	BP	1.60E-16

GO:1901362	organic cyclic compound biosynthetic process	BP	3.01E-16
GO:0016491	oxidoreductase activity	MF	3.51E-16
GO:0051274	beta-glucan biosynthetic process	BP	4.49E-16
GO:0004175	endopeptidase activity	MF	7.16E-16
GO:0051704	multi-organism process	BP	1.36E-15
GO:0005576	extracellular region	CC	2.06E-15
GO:0003677	DNA binding	MF	2.32E-15
GO:0042802	identical protein binding	MF	2.83E-15
GO:0004252	serine-type endopeptidase activity	MF	3.46E-15
GO:0044260	cellular macromolecule metabolic process	BP	3.64E-15
GO:0009505	plant-type cell wall	CC	3.71E-15
GO:0071554	cell wall organization or biogenesis	BP	1.08E-14
GO:0010583	response to cyclopentenone	BP	1.19E-14
GO:1901698	response to nitrogen compound	BP	1.22E-14
GO:0012505	endomembrane system	CC	1.34E-14
GO:0006073	cellular glucan metabolic process	BP	1.73E-14
GO:0019748	secondary metabolic process	BP	1.91E-14
GO:0016759	cellulose synthase activity	MF	1.92E-14
GO:0007169	transmembrane receptor protein tyrosine kinase signaling pathway	BP	1.93E-14
GO:0004857	enzyme inhibitor activity	MF	3.63E-14
GO:0048513	organ development	BP	9.78E-14
GO:0006928	cellular component movement	BP	1.07E-13
GO:0018130	heterocycle biosynthetic process	BP	1.29E-13
GO:0004650	polygalacturonase activity	MF	2.00E-13
GO:0050790	regulation of catalytic activity	BP	2.05E-13
GO:0044699	single-organism process	BP	3.81E-13
GO:0019538	protein metabolic process	BP	6.63E-13
GO:0016760	cellulose synthase (UDP-forming) activity	MF	7.43E-13
GO:0016070	RNA metabolic process	BP	7.98E-13
GO:0009725	response to hormone	BP	1.16E-12
GO:0009987	cellular process	BP	1.44E-12
GO:0015198	oligopeptide transporter activity	MF	2.48E-12
GO:0003774	motor activity	MF	7.39E-12
GO:0065009	regulation of molecular function	BP	9.94E-12
GO:0044707	single-multicellular organism process	BP	2.21E-11
GO:0016762	xyloglucan:xyloglucosyl transferase activity	MF	2.59E-11
GO:0032501	multicellular organismal process	BP	2.65E-11
GO:0044237	cellular metabolic process	BP	5.51E-11
GO:0016787	hydrolase activity	MF	1.20E-10
GO:0001101	response to acid chemical	BP	1.30E-10
GO:0042545	cell wall modification	BP	1.71E-10
GO:0044703	multi-organism reproductive process	BP	2.31E-10

GO:0009154	purine ribonucleotide catabolic process	BP	2.48E-10
GO:0009207	purine ribonucleoside triphosphate catabolic process	BP	2.48E-10
GO:0046130	purine ribonucleoside catabolic process	BP	2.48E-10
GO:0007154	cell communication	BP	2.85E-10
GO:0009808	lignin metabolic process	BP	3.21E-10
GO:0070011	peptidase activity, acting on L-amino acid peptides	MF	3.76E-10
GO:0006184	GTP catabolic process	BP	6.70E-10
GO:0051707	response to other organism	BP	6.81E-10
GO:0007166	cell surface receptor signaling pathway	BP	7.05E-10
GO:0030243	cellulose metabolic process	BP	7.30E-10
GO:0009605	response to external stimulus	BP	1.18E-09
GO:0009887	organ morphogenesis	BP	1.41E-09
GO:0048609	multicellular organismal reproductive process	BP	1.44E-09
GO:0005976	polysaccharide metabolic process	BP	2.05E-09
GO:0030244	cellulose biosynthetic process	BP	2.14E-09
GO:0009751	response to salicylic acid	BP	2.32E-09
GO:0007275	multicellular organismal development	BP	3.35E-09
GO:0033993	response to lipid	BP	3.92E-09
GO:0042936	dipeptide transporter activity	MF	3.95E-09
GO:0042938	dipeptide transport	BP	3.95E-09
GO:0044430	cytoskeletal part	CC	4.17E-09
GO:0042445	hormone metabolic process	BP	6.30E-09
GO:0009408	response to heat	BP	7.03E-09
GO:0048856	anatomical structure development	BP	7.13E-09
GO:0009607	response to biotic stimulus	BP	7.75E-09
GO:0010333	terpene synthase activity	MF	1.06E-08
GO:0007165	signal transduction	BP	1.28E-08
GO:0004712	protein serine/threonine/tyrosine kinase activity	MF	1.31E-08
GO:0006508	proteolysis	BP	1.40E-08
GO:0015630	microtubule cytoskeleton	CC	1.81E-08
GO:0042937	tripeptide transporter activity	MF	2.67E-08
GO:0007017	microtubule-based process	BP	2.94E-08
GO:0044700	single organism signaling	BP	2.98E-08
GO:0016131	brassinosteroid metabolic process	BP	3.10E-08
GO:0071705	nitrogen compound transport	BP	5.16E-08
GO:0008017	microtubule binding	MF	5.22E-08
GO:0046777	protein autophosphorylation	BP	5.40E-08
GO:1901136	carbohydrate derivative catabolic process	BP	6.31E-08
GO:0009733	response to auxin	BP	7.40E-08
GO:0009698	phenylpropanoid metabolic process	BP	9.14E-08
GO:0005871	kinesin complex	CC	9.60E-08
GO:0007018	microtubule-based movement	BP	1.30E-07
GO:0080044	quercetin 7-O-glucosyltransferase activity	MF	1.42E-07

GO:0032502	developmental process	BP	1.55E-07
GO:0097305	response to alcohol	BP	3.31E-07
GO:0019439	aromatic compound catabolic process	BP	3.33E-07
GO:0048544	recognition of pollen	BP	4.43E-07
GO:0003777	microtubule motor activity	MF	4.49E-07
GO:0044767	single-organism developmental process	BP	5.79E-07
GO:0009653	anatomical structure morphogenesis	BP	7.28E-07
GO:0042939	tripeptide transport	BP	1.15E-06
GO:0000976	transcription regulatory region sequence-specific DNA binding	MF	1.18E-06
GO:0009620	response to fungus	BP	1.36E-06
GO:0034768	(E)-beta-ocimene synthase activity	MF	1.41E-06
GO:0050551	myrcene synthase activity	MF	1.41E-06
GO:0071805	potassium ion transmembrane transport	BP	1.72E-06
GO:0003924	GTPase activity	MF	1.86E-06
GO:0010016	shoot system morphogenesis	BP	1.96E-06
GO:0045229	external encapsulating structure organization	BP	2.17E-06
GO:0071555	cell wall organization	BP	2.17E-06
GO:0048827	phyllome development	BP	2.34E-06
GO:0031425	chloroplast RNA processing	BP	2.34E-06
GO:0006970	response to osmotic stress	BP	2.52E-06
GO:0009845	seed germination	BP	2.81E-06
GO:0048438	floral whorl development	BP	3.95E-06
GO:0006950	response to stress	BP	5.05E-06
GO:1901361	organic cyclic compound catabolic process	BP	5.06E-06
GO:0042127	regulation of cell proliferation	BP	5.30E-06
GO:0019953	sexual reproduction	BP	5.79E-06
GO:0004499	N,N-dimethylaniline monooxygenase activity	MF	6.20E-06
GO:0048359	mucilage metabolic process involved in seed coat development	BP	6.21E-06
GO:0050832	defense response to fungus	BP	6.53E-06
GO:0005975	carbohydrate metabolic process	BP	6.84E-06
GO:0050502	cis-zeatin O-beta-D-glucosyltransferase activity	MF	7.39E-06
GO:0050403	trans-zeatin O-beta-D-glucosyltransferase activity	MF	7.39E-06
GO:0035197	siRNA binding	MF	7.39E-06
GO:0051753	mannan synthase activity	MF	7.39E-06
GO:0000148	1,3-beta-D-glucan synthase complex	CC	7.39E-06
GO:0003843	1,3-beta-D-glucan synthase activity	MF	7.39E-06
GO:0006075	(1->3)-beta-D-glucan biosynthetic process	BP	7.39E-06
GO:0017111	nucleoside-triphosphatase activity	MF	7.50E-06
GO:0010334	sesquiterpene synthase activity	MF	7.96E-06
GO:0016165	linoleate 13S-lipoxygenase activity	MF	7.96E-06
GO:0030048	actin filament-based movement	BP	7.96E-06

GO:0009856	pollination	BP	8.89E-06
GO:0009404	toxin metabolic process	BP	1.10E-05
GO:0010214	seed coat development	BP	1.52E-05
GO:0005856	cytoskeleton	CC	1.69E-05
GO:0048367	shoot system development	BP	1.72E-05
GO:0015698	inorganic anion transport	BP	2.01E-05
GO:0009816	defense response to bacterium, incompatible interaction	BP	2.25E-05
GO:0005874	microtubule	CC	2.31E-05
GO:0048443	stamen development	BP	2.42E-05
GO:0006913	nucleocytoplasmic transport	BP	2.65E-05
GO:0048437	floral organ development	BP	2.95E-05
GO:0009832	plant-type cell wall biogenesis	BP	3.34E-05
GO:0044267	cellular protein metabolic process	BP	3.71E-05
GO:0009266	response to temperature stimulus	BP	3.74E-05
GO:0043565	sequence-specific DNA binding	MF	3.74E-05
GO:0009699	phenylpropanoid biosynthetic process	BP	4.26E-05
GO:0006979	response to oxidative stress	BP	4.66E-05
GO:0010047	fruit dehiscence	BP	4.66E-05
GO:0045309	protein phosphorylated amino acid binding	MF	4.66E-05
GO:0044550	secondary metabolite biosynthetic process	BP	4.69E-05
GO:0016620	oxidoreductase activity, acting on the aldehyde or oxo group of donors, NAD or NADP as acceptor	MF	4.70E-05
GO:0018685	alkane 1-monooxygenase activity	MF	4.70E-05
GO:0008395	steroid hydroxylase activity	MF	4.70E-05
GO:0016099	monoterpenoid biosynthetic process	BP	4.70E-05
GO:0080001	mucilage extrusion from seed coat	BP	5.19E-05
GO:0080046	quercetin 4'-O-glucosyltransferase activity	MF	5.19E-05
GO:0009809	lignin biosynthetic process	BP	5.37E-05
GO:0030246	carbohydrate binding	MF	6.25E-05
GO:0010817	regulation of hormone levels	BP	6.62E-05
GO:0007264	small GTPase mediated signal transduction	BP	7.27E-05
GO:0009407	toxin catabolic process	BP	7.67E-05
GO:0000910	cytokinesis	BP	8.31E-05
GO:0009651	response to salt stress	BP	8.39E-05
GO:0009628	response to abiotic stimulus	BP	8.44E-05
GO:0009753	response to jasmonic acid	BP	8.58E-05
GO:0035556	intracellular signal transduction	BP	9.10E-05
GO:0007276	gamete generation	BP	9.38E-05
GO:0005886	plasma membrane	CC	9.92E-05
GO:0048366	leaf development	BP	0.000106205
GO:0048232	male gamete generation	BP	0.000119687
GO:0045735	nutrient reservoir activity	MF	0.000119687
GO:0004364	glutathione transferase activity	MF	0.00012357

GO:0004185	serine-type carboxypeptidase activity	MF	0.00012357
GO:0009965	leaf morphogenesis	BP	0.000144434
GO:0009741	response to brassinosteroid	BP	0.000147089
GO:0034637	cellular carbohydrate biosynthetic process	BP	0.000156937
GO:0048235	pollen sperm cell differentiation	BP	0.000164292
GO:0015020	glucuronosyltransferase activity	MF	0.000194474
GO:0048569	post-embryonic organ development	BP	0.000194864
GO:0061640	cytoskeleton-dependent cytokinesis	BP	0.000212723
GO:0016459	myosin complex	CC	0.000232865
GO:0015103	inorganic anion transmembrane transporter activity	MF	0.000233148
GO:0009833	primary cell wall biogenesis	BP	0.000255184
GO:0080016	(-)-E-beta-caryophyllene synthase activity	MF	0.000294794
GO:0051762	sesquiterpene biosynthetic process	BP	0.000294794
GO:0080017	alpha-humulene synthase activity	MF	0.000294794
GO:0000080	mitotic G1 phase	BP	0.000294794
GO:0008202	steroid metabolic process	BP	0.000331775
GO:0044262	cellular carbohydrate metabolic process	BP	0.000354998
GO:0009617	response to bacterium	BP	0.000360356
GO:0048646	anatomical structure formation involved in morphogenesis	BP	0.000360395
GO:0009737	response to abscisic acid	BP	0.000372069
GO:0046700	heterocycle catabolic process	BP	0.000444942
GO:0019904	protein domain specific binding	MF	0.00044652
GO:0022412	cellular process involved in reproduction in multicellular organism	BP	0.000470362
GO:0022403	cell cycle phase	BP	0.000470362
GO:0046274	lignin catabolic process	BP	0.000499522
GO:0048731	system development	BP	0.000508677
GO:0009814	defense response, incompatible interaction	BP	0.00058654
GO:0009986	cell surface	CC	0.000679527
GO:0009838	abscission	BP	0.000679527
GO:0006952	defense response	BP	0.000812351
GO:0071669	plant-type cell wall organization or biogenesis	BP	0.000830722
GO:0080043	quercetin 3-O-glucosyltransferase activity	MF	0.000844991
GO:0022414	reproductive process	BP	0.000904792
GO:0042335	cuticle development	BP	0.000989272
GO:0010089	xylem development	BP	0.001022617
GO:0010160	formation of organ boundary	BP	0.001022617
GO:0016903	oxidoreductase activity, acting on the aldehyde or oxo group of donors	MF	0.001124777
GO:0010150	leaf senescence	BP	0.001173439
GO:0016597	amino acid binding	MF	0.001333089
GO:0048645	organ formation	BP	0.001367202
GO:0030234	enzyme regulator activity	MF	0.001395263

GO:0030397	membrane disassembly	BP	0.001401707
GO:0004708	MAP kinase kinase activity	MF	0.001401707
GO:0030247	polysaccharide binding	MF	0.001421607
GO:0031406	carboxylic acid binding	MF	0.001584965
GO:0000003	reproduction	BP	0.001837169
GO:0010304	PSII associated light-harvesting complex II catabolic process	BP	0.001872603
GO:0051322	anaphase	BP	0.001872603
GO:0009850	auxin metabolic process	BP	0.001882131
GO:0009954	proximal/distal pattern formation	BP	0.002028951
GO:0051510	regulation of unidimensional cell growth	BP	0.002028951
GO:0016621	cinnamoyl-CoA reductase activity	MF	0.002028951
GO:0042178	xenobiotic catabolic process	BP	0.002028951
GO:0055085	transmembrane transport	BP	0.002102985
GO:0009624	response to nematode	BP	0.002204093
GO:0010103	stomatal complex morphogenesis	BP	0.00227649
GO:0004197	cysteine-type endopeptidase activity	MF	0.002603785
GO:0042446	hormone biosynthetic process	BP	0.002603785
GO:0004176	ATP-dependent peptidase activity	MF	0.002985237
GO:0044270	cellular nitrogen compound catabolic process	BP	0.003215547
GO:0098542	defense response to other organism	BP	0.003250017
GO:0016709	oxidoreductase activity, acting on paired donors, with incorporation or reduction of molecular oxygen, NAD(P)H as one donor, and incorporation of one atom of oxygen	MF	0.00332882
GO:0097306	cellular response to alcohol	BP	0.003522738
GO:0055046	microgametogenesis	BP	0.003530053
GO:0016132	brassinosteroid biosynthetic process	BP	0.003685284
GO:0016114	terpenoid biosynthetic process	BP	0.003940307
GO:0008361	regulation of cell size	BP	0.003940307
GO:0010345	suberin biosynthetic process	BP	0.003940307
GO:0003841	1-acylglycerol-3-phosphate O-acyltransferase activity	MF	0.003940307
GO:0006813	potassium ion transport	BP	0.004016775
GO:0010199	organ boundary specification between lateral organs and the meristem	BP	0.004184748
GO:0015116	sulfate transmembrane transporter activity	MF	0.004184748
GO:0045087	innate immune response	BP	0.004310796
GO:0044462	external encapsulating structure part	CC	0.004706199
GO:0016106	sesquiterpenoid biosynthetic process	BP	0.004706199
GO:0010374	stomatal complex development	BP	0.005086836
GO:0044702	single organism reproductive process	BP	0.00601932
GO:0008356	asymmetric cell division	BP	0.006206924
GO:0000038	very long-chain fatty acid metabolic process	BP	0.006206924
GO:0048653	anther development	BP	0.006267152
GO:1901565	organonitrogen compound catabolic process	BP	0.006337002

GO:0009791	post-embryonic development	BP	0.006403337
GO:0071396	cellular response to lipid	BP	0.006933045
GO:0090304	nucleic acid metabolic process	BP	0.007121867
GO:0009897	external side of plasma membrane	CC	0.007183394
GO:0010227	floral organ abscission	BP	0.007183394
GO:0010942	positive regulation of cell death	BP	0.007232285
GO:0080092	regulation of pollen tube growth	BP	0.007232285
GO:0015706	nitrate transport	BP	0.007232285
GO:0080045	quercetin 3'-O-glucosyltransferase activity	MF	0.007232285
GO:0045298	tubulin complex	CC	0.007232285
GO:0016847	1-aminocyclopropane-1-carboxylate synthase activity	MF	0.007232285
GO:0004630	phospholipase D activity	MF	0.007232285
GO:0009789	positive regulation of abscisic acid-activated signaling pathway	BP	0.007592543
GO:0055028	cortical microtubule	CC	0.007592543
GO:0016411	acylglycerol O-acyltransferase activity	MF	0.007592543
GO:0009414	response to water deprivation	BP	0.00820218
GO:0009044	xylan 1,4-beta-xylosidase activity	MF	0.008516561
GO:0043531	ADP binding	MF	0.008516561
GO:0010358	leaf shaping	BP	0.008516561
GO:0042742	defense response to bacterium	BP	0.008564816
GO:0009738	abscisic acid-activated signaling pathway	BP	0.0096893
GO:0050661	NADP binding	MF	0.01027941
GO:1901360	organic cyclic compound metabolic process	BP	0.010814294
GO:0031426	polycistronic mRNA processing	BP	0.010814294
GO:0080086	stamen filament development	BP	0.010814294
GO:0098581	detection of external biotic stimulus	BP	0.010814294
GO:0010068	protoderm histogenesis	BP	0.010814294
GO:0009786	regulation of asymmetric cell division	BP	0.010814294
GO:0015334	high-affinity oligopeptide transporter activity	MF	0.010814294
GO:0043201	response to leucine	BP	0.010814294
GO:0080053	response to phenylalanine	BP	0.010814294
GO:0080052	response to histidine	BP	0.010814294
GO:0007584	response to nutrient	BP	0.010814294
GO:0009830	cell wall modification involved in abscission	BP	0.010814294
GO:0070825	micropyle	CC	0.010814294
GO:0045697	regulation of synergid differentiation	BP	0.010814294
GO:0080006	internode patterning	BP	0.010814294
GO:0005546	phosphatidylinositol-4,5-bisphosphate binding	MF	0.010814294
GO:0010012	steroid 22-alpha hydroxylase activity	MF	0.010814294
GO:0045962	positive regulation of development, heterochronic	BP	0.010814294
GO:0080019	fatty-acyl-CoA reductase (alcohol-forming) activity	MF	0.010814294
GO:0002376	immune system process	BP	0.011454374

GO:0052689	carboxylic ester hydrolase activity	MF	0.011746409
GO:0048829	root cap development	BP	0.012225789
GO:0046246	terpene biosynthetic process	BP	0.012225789
GO:0009975	cyclase activity	MF	0.012225789
GO:0008272	sulfate transport	BP	0.012225789
GO:0009735	response to cytokinin	BP	0.013300833
GO:0009886	post-embryonic morphogenesis	BP	0.013519857
GO:0030145	manganese ion binding	MF	0.014122478
GO:0051716	cellular response to stimulus	BP	0.014421582
GO:0070887	cellular response to chemical stimulus	BP	0.014828811
GO:0008540	proteasome regulatory particle, base subcomplex	CC	0.015395727
GO:0022603	regulation of anatomical structure morphogenesis	BP	0.016790228
GO:0080156	mitochondrial mRNA modification	BP	0.016790228
GO:0004022	alcohol dehydrogenase (NAD) activity	MF	0.016790228
GO:0008285	negative regulation of cell proliferation	BP	0.016790228
GO:0010565	regulation of cellular ketone metabolic process	BP	0.017863334
GO:0009787	regulation of abscisic acid-activated signaling pathway	BP	0.019280091
GO:0009739	response to gibberellin	BP	0.019682491
GO:0019199	transmembrane receptor protein kinase activity	MF	0.019841446
GO:0048441	petal development	BP	0.019841446
GO:0030154	cell differentiation	BP	0.020270219
GO:0044710	single-organism metabolic process	BP	0.020465877
GO:0007568	aging	BP	0.021055963
GO:0006725	cellular aromatic compound metabolic process	BP	0.021055963
GO:0008299	isoprenoid biosynthetic process	BP	0.021059249
GO:0071229	cellular response to acid chemical	BP	0.021437198
GO:0044248	cellular catabolic process	BP	0.024418293
GO:0048364	root development	BP	0.025169704
GO:0005199	structural constituent of cell wall	MF	0.025381833
GO:0008283	cell proliferation	BP	0.025381833
GO:0009637	response to blue light	BP	0.025381833
GO:0008762	UDP-N-acetylmuramate dehydrogenase activity	MF	0.025381833
GO:0005484	SNAP receptor activity	MF	0.025381833
GO:0048638	regulation of developmental growth	BP	0.025507767
GO:0009556	microsporogenesis	BP	0.027470675
GO:0044271	cellular nitrogen compound biosynthetic process	BP	0.02765035
GO:0004805	trehalose-phosphatase activity	MF	0.02791553
GO:0040008	regulation of growth	BP	0.031473918
GO:0015112	nitrate transmembrane transporter activity	MF	0.032049397
GO:0010540	basipetal auxin transport	BP	0.032049397
GO:0000165	MAPK cascade	BP	0.032049397
GO:0016688	L-ascorbate peroxidase activity	MF	0.032049397
GO:0003006	developmental process involved in reproduction	BP	0.03290363

GO:0009056	catabolic process	BP	0.032981462
GO:0043068	positive regulation of programmed cell death	BP	0.032981462
GO:0004709	MAP kinase kinase kinase activity	MF	0.032981462
GO:0048830	adventitious root development	BP	0.032981462
GO:0015700	arsenite transport	BP	0.032981462
GO:0015105	arsenite transmembrane transporter activity	MF	0.032981462
GO:0050378	UDP-glucuronate 4-epimerase activity	MF	0.032981462
GO:0005544	calcium-dependent phospholipid binding	MF	0.032981462
GO:0004301	epoxide hydrolase activity	MF	0.032981462
GO:0000281	mitotic cytokinesis	BP	0.035966818
GO:0001558	regulation of cell growth	BP	0.036400756
GO:0044712	single-organism catabolic process	BP	0.037888134
GO:1900055	regulation of leaf senescence	BP	0.038474061
GO:0009851	auxin biosynthetic process	BP	0.038474061
GO:0006633	fatty acid biosynthetic process	BP	0.043629949
GO:0008092	cytoskeletal protein binding	MF	0.044194931
GO:0098662	inorganic cation transmembrane transport	BP	0.046219143
GO:0005200	structural constituent of cytoskeleton	MF	0.047657584
GO:0034293	sexual sporulation	BP	0.047657584
GO:0009755	hormone-mediated signaling pathway	BP	0.048798918

† MF: molecular function; BP: biological process; CC: cellular component. * Fisher's exact test, corrected by false discovery rate.

Supplementary Table 17 | BUSCO (Benchmarking Universal Single-Copy Orthologs) results for 14 plant genomes. The recovered genes in tested genome are classified as 'complete' when their lengths are within two standard deviations of the BUSCO group mean length (i.e. within ~95% expectation). 'Complete' genes found with more than one copy are classified as 'duplicated'. Genes only partially recovered are classified as 'fragmented', and genes not recovered are classified as 'missing'.

Species	Complete Single-Copy BUSCOs (%)	Complete Duplicated BUSCOs (%)	Fragmented BUSCOs (%)	Missing BUSCOs (%)
Ananas comosus	94.87	18.20	2.20	2.93
Arabidopsi thaliana	97.18	16	0.63	2.2
Amborella trichopoda	93.41	12.13	2.72	3.87
Dendrobium officinale	89.02	13.08	4.92	6.07
Elaeis guineensis	95.08	21.65	1.67	3.24
Gastrodia elata	67.15	9.21	4.39	28.45
Glycine max	95.61	66.21	1.15	3.24
Oryza sativa	97.91	13.91	0.84	1.26
Picea abies	45.71	11.51	14.96	39.33
Phalaenopsis equestris	81.90	12.03	5.33	12.76
Physcomitrella patens	88.28	22.07	2.72	9
Populus trichocarpa	96.65	24.27	1.05	2.3
Vitis vinifera	95.4	14.54	1.88	2.72
Zea mays	96.13	20.08	1.36	2.51

Supplementary Table 18 | Exon mapping rate comparison between *G. elata*, *O. sativa* and *P. equestris*. RNA sequencing reads were mapped to the genome, percent of reads that mapped to exon regions were denoted as exon mapping rate. Exon mapping rate was used as a measure of gene annotation quality.

Species	Sample	Reads mapped in exon region (%)	Reads mapped in intron region (%)	Reads mapped in intergenic region (%)
G. elata	Immature tuber	88.56	4.69	6.76
		87.94	5.58	6.48
		90.00	3.99	6.01
	Stem	87.66	3.84	8.50
		86.28	4.99	8.74
		86.82	4.47	8.71
	Flower	90.06	3.93	6.01
		87.99	5.24	6.77
		89.46	4.33	6.21
	Mature tuber	88.19	4.90	6.91
		87.79	5.37	6.85
		87.31	5.36	7.34
	Juvenile tuber	86.39	5.97	7.64
		87.80	5.13	7.07
		87.71	5.20	7.09
	Innutritious tuber	88.20	5.01	6.79
		91.81	2.55	5.65
		90.70	3.52	5.78
	Protocorm	89.74	3.91	6.35
O. sativa	Leaf	76.10	1.87	22.03
		80.26	1.83	17.91
		82.30	1.14	16.56
P. equestris	Stem	75.10	8.80	16.10
	Flower bud	84.36	2.95	12.69
	Sepal	83.12	3.28	13.60
	lip	77.46	6.28	16.26
	petal	81.20	3.58	15.21
	root	68.88	12.79	18.33

Supplementary Table 19 | Detection of gene deletion events in *G. elata* through syntenic region comparisons.

Gene deletion events	Presented in *P. equestris* but deleted in *G. elata*	Presented in *D. officinale* but deleted in *G. elata*
Total number of detected syntenic gene deletion	2,961	3,120
True events*	2,891	3,059
Missing†	2,812	2,986
Decayed pseudogene‡	39	38
Truncated pseudogenes§	40	35
False positives#	70 (11)	61 (8)

* Number of deletion after excluding false positives;

† Could not predict gene structure from seed gene;

‡ Predicted gene fragments contain frameshift or premature stop codons;

§ Predicted gene fragments match less than 70% of the length of the seed gene;

# Putative unannotated genes: predicted gene fragments match more than 70% of the length of the seed gene, and did not contain frameshift or premature stop codon mutations; Numbers in the bracket were number of gene fragments that were supported by RNA-seq data.

Supplementary Table 20 | Details of the primers of PCR verification of loss genes in *G. elata*.

Gene Name	Gene Function	Forward Primer (5' to 3')	Reverse Primer (5' to 3')
atpD	ATP synthase	TTCCTCAACGTCCTCGTC	TTCACGCTCATATCAATAAGCTT
atpG	ATP synthase	ATGTCAATGAAGGCAAAGG	ACCAAGCCTCTCTCCATTC
IhcA	Photosystem I	GATTTCGGTTTCGATCCTCT	TCTTAATCTCTTTAGTCCTCAAAAC
IhcB	Photosystem II	TGGGTCGATTTCTTCAACC	CCCGGCCTCAAAGTAGAA
petC	Cytochrome b6f	CAAGGATTGAAGGGAGATCC	CTGAAATCAGTTTCCACCCA
psaD	Photosystem I	CACCCCATCACCAATCTTCG	ACTGGTAATTGATCTTGTACTTGGA
psaF	Photosystem I	TTCGACAACTACGGGAAGT	GGGACATCGATGATGATCTC
psaL	Photosystem I	TGGTACCTTTCCAACCTCC	GTAGAGGAGGAAGTAAGCCCA
psaN	Photosystem I	ATGGCGACCATGAACTCAA	ACACCATTTCCAGAAAACATT
psb27	Photosystem II	TTGGGTGGCCAAGTACCG	TCTCAGCGGTGTCCATCTC
psb28	Photosystem II	ATCCAAGGGACGGATGAG	ATAAACCTCATGAACCTGTCC
psbO	Photosystem II	GATACAGAGCAAGACGTACATG	CTGTCTCCGGTTTGCTCTT
psbR	Photosystem II	CAAGTATGGAGCTAACGTCG	GAGCCAAAGCACTTGTGTT
psbY	Photosystem II	TGGGTCCTTTACAACATCCT	CCCAGACCTCATCTTGTTGA
petE	Rubisco	GAGAAGATTGTGTTCAAGAACAA	CGGTGACTTTACCCACCAT
ICS	Isochorismate synthase	CCTCAGGTTGAGTTTGATGAG	CCGGCATACATTCCTCTATC
DHAR1	Dehydroascorbate reductase	TTGTGACGTTCTTGAAAAGCAA	CCAAGAGCAACCTCAAGATG
DHAR2	Dehydroascorbate reductase	CTGAAAAGGCTTCAGTTGG	CATCCAGCAATTACATCCTC
TRX-H	Thioredoxin H-type	GTGGTTGATTTCACGGCT	CTTTCTTGGCACCGACAA
TRX-M	Thioredoxin M-type	TTTTGGGCACCATGGTGT	GACTGTATCCTTCTTCTCTCCAT

Supplementary Table 21 | Number of pseudogenes and their types of *G. elata* were identified.

**Pseudogene type**	**DUP***	**FRAG†**	**PSSD‡**	**FP§**	**High Confidence**	**Sum**
P. equestris	398	413	65	4	876	880
D. officinale	461	540	79	16	1,080	1096

* DUP: duplicated pseudogene; † FRAG: fragmented pseudogene;

‡ PSSD: Processed/retrotransposed pseudogenes; § FP: false positives.

Supplementary Table 22 | GO terms significantly enriched in pseudogene of *G. elata* using *D. officinale* genes as a reference.

GO ID	GO Term	GO Class	Adjusted P-value*
GO:0006278	RNA-dependent DNA replication	BP	1.89E-11
GO:0003964	RNA-directed DNA polymerase activity	MF	3.83E-11
GO:0034061	DNA polymerase activity	MF	3.05E-10
GO:0006260	DNA replication	BP	2.46E-07
GO:0016779	nucleotidyltransferase activity	MF	2.29E-05
GO:0006259	DNA metabolic process	BP	6.74E-05
GO:0000313	organellar ribosome	CC	0.012554903
GO:0016772	transferase activity, transferring phosphorus-containing groups	MF	0.018854934
GO:0003676	nucleic acid binding	MF	0.031778142
GO:0009547	plastid ribosome	CC	0.04236502

*Fisher's exact test, corrected by false discovery rate.

Supplementary Table 23 | GO terms significantly enriched in pseudogene of *G. elata* using *P. equestris* genes as a reference.

GO ID	GO Term	GO Class	Adjusted P-value*
GO:0009547	plastid ribosome	CC	0.000122231
GO:0000313	organellar ribosome	CC	0.000244006
GO:0000311	plastid large ribosomal subunit	CC	0.019531626
GO:1902589	single-organism organelle organization	BP	0.045016499

*Fisher’s exact test, corrected by false discovery rate.

Supplementary Table 24 | Genome composition of the orchid genomes.

Annotation		*G. elata*		*P. equestris*	
		**Length (bp)**	**Percent**	**Length (bp)**	**Percent**
Genic		293,048,294	27.62%	283,578,300	26.65%
	Exon	28,820,953	2.72%	26,389,941	2.48%
	Intron	264,227,341	24.90%	257,188,359	24.17%
Intergenic					
TEs and repeats		702,250,876	66.18%	633,166,320	59.51%
	LTR retrotransposon	593,527,755	55.94%	491,966,301	46.24%
	DNA transposon	46,521,569	4.38%	45,683,395	4.29%
Non-coding RNA		799,049	0.07%		
Pseudogene		768,453	0.07%		
N		35,579,041	3.35%	82,558,897	7.60%
Assembled genome length		1,061,091,640	---	1,064,051,384	----

Supplementary Table 25 | Whole genome alignment results between *G. elata* and *P. equestris*. LastZ was used to align the genomes, with the parameter "M = 254 K = 2000 L = 3000 Y = 3000 T = 1 – gf extend – no chain – gapped".

Species	Genome size (bp)	Aligned size (bp)	Aligned percentage
G. elata	1,061,091,640	312,559,265	29.46%
P. equestris	1,064,051,384	315,236,337	29.63%

Supplementary Table 26 | Distance to closest TE for different types of gene loss events.

	Number	Average distance	P-value
**Pseudogenes***	1359	937.36	< 2.2e-16
**SV genes$**	487	606.22	< 2.2e-16
**Functional genes#**	18969	388.48	

*. Combination of Pseudogene locus predicted from *P. equestris* and *D. officinale* genes

$. Genes lost in *G. elata* due to insertions or deletions between *P. equestris* and *G. elata*

#. Functional genes of *G. elata*

P-value: Wilcox rank sum test, Pseudogenes vs. Functional genes and SV genes vs. Functional genes.

Supplementary Table 27 | Analysis of defense related genes in the *G. elata* genome compared to *Phalaenopsis equestris*, *Dendrobium officinale*, *Ananas comosus* and *Arabidopsis thaliana.*

Defense related genes and pathways	Gene name	Gel[*]	Peq[†]	Dof[‡]	Aco[§]	Ath[‖]	P1[¶]	P2[#]	P3[☆]	P4[**]	P5[††]
Jasmonate (JA) biosynthetic pathway	JA amino acid synthetase	2	2	2	2	1	0.3553	0.5714	1.0000	0.6233	1.0000
	Lipoxygenase	4	7	5	12	6	1.0000	1.0000	0.2141	0.7686	0.1154
	Allene oxide synthase	3	4	4	3	1	0.4487	0.3117	0.6959	0.7199	0.4308
	Allene oxide cyclase	1	1	1	1	4	1.0000	0.6547	1.0000	0.5362	0.2770
	12-oxophytodienoate reductase	6	9	5	8	4	0.4357	0.3348	1.0000	0.4252	1.0000
	acyl-CoA-oxidase (β-oxidation)	6	5	6	6	6	0.2765	0.5655	0.5680	0.2428	0.8505
Salicylic acid (SA) synthetic pathway	Phenylalanine ammonia-lyase	4	3	3	6	4	0.3426	0.7237	1.0000	0.2480	0.3640
	Isochorismate synthase (ICS)	0	1	1	1	4	-	-	-	-	-
	Isochorismate pyruvate lyase (IPL)	1	1	1	1	3	1.0000	0.6495	1.0000	0.5362	0.4445
Ethylene (ET) synthetic pathway	1-aminocyclopropane-1-carboxylate oxidase	2	10	5	3	5	0.5668	0.7079	1.0000	0.3918	0.5420
	S-adenosyl-L-methionine synthetase	4	3	4	4	3	0.2967	0.4545	0.7249	0.2826	1.0000
	1-aminocyclopropane-1-carboxylate synthase	3	6	7	3	11	0.7906	0.1779	0.6959	1.0000	0.4565
Antioxidant system	Superoxide dismutase (SOD)	3	10	9	8	9	0.3559	0.3812	0.5422	0.4460	0.6244
	Ascorbate peroxidase (APX)	6	16	9	11	8	1.0000	1.0000	0.8064	0.8308	0.8860
	Glutathione-disulfide reductase (GR)	2	4	5	2	2	1.0000	1.0000	1.0000	1.0000	0.4308

	Catalase (CAT)	4	3	4	3	3	0.2774	0.4545	0.4572	0.2826	0.8087
	NADPH Oxidase	3	6	2	11	1	0.7487	0.3117	0.3808	0.7039	0.3640
	NADPH Oxidase-like Alternative oxidase (AOX)	2	12	8	6	6	0.3128	0.4848	0.4834	0.1991	0.7268
	Monodehydroascorbate reductase (MDAR)	4	4	4	6	5	0.5458	1.0000	1.0000	0.4866	0.4025
	Dehydroascorbate reductase (DHAR)	0	0	1	1	9	-	-	-	-	-
	Ferritin	1	1	1	1	4	1.0000	0.6547	1.0000	0.5362	0.2770
	Peroxiredoxin (PrxR)	2	10	16	5	4	0.2264	1.0000	0.7073	0.0669	0.0654
	Thioredoxins (Trx)	0	1	1	1	8	-	-	-	-	-
Programmed cell death (PCD)	Metacaspase (MCP)	3	8	6	5	9	0.7925	0.3812	1.0000	0.7778	0.5820
**Nucleotide-binding site family (NBS)**	**Nucleotide-binding site family (NBS)**	**6**	**55**	**124**	**163**	**141**	**4.27E-23**	**9.16E-25**	**5.41E-30**	**1.64E-13**	**3.41E-24**
**Pattern recognition receptor (PRR)**	**pattern recognition receptor (PRR)**	**55**	**70**	**79**	**127**	**379**	**9.81E-07**	**1.03E-37**	**0.00248**	0.1537	**6.46E-66**
**Ca^{2+} proteins**	**Calmodulin-1 (CAM)**	**1**	**8**	**19**	**9**	**15**	**0.0077**	**0.0039**	**0.0545**	**0.0116**	0.3230
	**Calcium-binding protein (CML)**	**8**	**41**	**43**	**20**	**14**	**0.0133**	0.8290	0.1854	**0.0007**	**0.0045**
	**Calcium-dependent protein kinase (CPK)**	**5**	**22**	**35**	**35**	**62**	**1.34E-05**	**1.84E-09**	**0.0001**	**0.0037**	**5.12E-08**
**Pathogenesis related protein (PRs)**	PR1	5	14	18	11	49	0.0113	4.47E-07	0.4598	0.2386	9.21E-06
	PR2	0	0	0	0	7	-	-	-	-	-
	PR3，4，8，11	10	14	24	16	24	0.6419	0.2218	0.8441	0.8643	0.2749
	PR5	14	24	23	21	20	1.0000	1.0000	1.0000	1.0000	0.9193
	PR6	0	0	0	0	15	-	-	-	-	-

	PR7	1	1	0	1	3	0.5871	0.6495	1.0000	0.4008	0.2200
	PR9	31	55	46	81	108	0.0344	**5.44E-06**	0.003757	0.8349	2.36E-12
	PR10	1	6	5	4	1	**0.4977**	**1.0000**	0.6549	0.3204	**0.4659**
	PR12	2	4	3	2	4	1.0000	1.0000	1.0000	1.0000	1.0000
	PR13	0	0	0	0	288	-	-	-	-	-
	PR14	2	6	5	8	29	0.0402	2.76E-05	0.2126	0.7452	7.77E-07
	PR15.16	7	10	11	34	41	0.0435	0.0002	0.0013	0.8209	**8.39E-12**
	**Total**	**73**	**134**	**135**	**178**	**587**	**1.00E-13**	**1.08E-63**	**8.17E-05**	**0.6501**	**2.93E-88**
**Monocot mannose-binding lectin antifungal protein of *G. elata* (GAFP)**	**GAFP**	**20**	**17**	**25**	**3**	**0**	**0.0004**	**1.72E-08**	**1.29E-05**	**0.0928**	**1.25E-10**

*. Gel: *Gastrodia elata*; †. Peq: *Phalaenopsis equestris*; ‡. Dof: *Dendrobium officinale*; §. Aco: *Ananas comosus*; ‖. Ath: *Arabidopsis thaliana*; ¶. P1: Fisher's exact test of gene number in *G. elata* genome compared to *P. equestris*, *D. officinale*, *A. comosus* and *A. thaliana*; #. P2: Fisher's exact test of gene number in *G. elata* genome compared to *A. thaliana*; ☆.P3: Fisher's exact test of gene number in *G. elata* genome compared to *A. comosus*; **. P4: Fisher's exact test of gene number in *G. elata* genome compared to *P. equestris* and *D. officinale*; ††. P5: Fisher's exact test of gene number in *G. elata*, *P. equestris* and *D. officinale* genome compared to *A. comosus* and *A. thaliana*. The contracted/expanded gene sets were highlighted in bold format ($P<0.05$).

Supplementary Table 28 | Nuclear genes coding polypeptide subunits of protein complexes of the photosynthetic apparatus.

Polypeptide subunit of protein complexes	Nuclear gene	*Gastrodia elata*	*Phalaenopsis equestris*	*Dendrobium officinale*	*Ananas comosus*	*Arabidopsis thaliana*
ATPsyn	ATPsyn	7	11	11	16	15
cytb6f	petC	0	4	4	4	4
	petM	0	1	1	1	1
phoI	phoI	0	4	4	4	4
	psaD	0	1	1	1	2
	psaE	0	1	1	1	2
	psaF	0	1	0	1	1
	psaG	0	3	1	0	1
	psaH	0	1	1	1	2
	psaL	0	1	1	1	1
	psaO	0	1	1	1	1
	psaP	0	1	1	3	2
phoII	lhc	0	11	13	14	18
	phoII	0	12	7	15	1
	psb27	0	2	2	2	2
	psb28	0	1	1	1	1
	psb29	1	1	1	1	1
	psbO	0	1	1	1	2
	psbP	1	11	10	13	12
	psbQ	2	2	2	5	5
	psbR	0	1	1	1	1
	psbS	0	1	1	1	1
	psbW	0	1	1	1	1
	psbX	0	0	1	0	1
	psbY	0	1	1	0	1
Rubisco	petE	0	3	5	1	5
	rbcS	1	1	2	2	4
Total		12	80	77	93	94

Supplementary Table 29 | Plastid genes coding polypeptide subunits of protein complexes of the photosynthetic apparatus.

**Polypeptide subunits of protein complex**	**Plastid coding genes**	***Gastrodia elata***	***Phalaenopsis equestris***	***Dendrobium officinale***
ATP synthase	atpA	0	1	1
	atpB	0	1	1
	atpE	0	1	1
	atpF	0	1	1
	atpH	0	1	1
	atpI	0	1	1
Cytochrome b/f complex	petA	0	1	1
	petB	0	1	1
	petD	0	1	1
	petG	0	1	1
	petL	0	1	1
	petN	0	1	1
Subunits of photosystem Ⅰ	psaB	0	1	1
	psaC	0	1	1
	psaI	0	1	1
	psaJ	0	1	1
	ycf3	0	1	1
Subunits of photosystem Ⅱ	psbA	0	1	1
	psbB	0	1	1
	psbC	0	1	1
	psbD	0	1	1
	psbE	0	1	1
	psbH	0	1	1
	psbI	0	1	1
	psbJ	0	1	1
	psbK	0	1	1
	psbL	0	1	1
	psbM	0	1	1
	psbN	0	1	1
	psbT	0	1	1
Rubisco	rbcL	0	1	1
NADH-dehydrogenase like complex	ndhA	0	0	1
	ndhB	0	pseudogene	2
	ndhC	0	pseudogene	1
	ndhD	0	pseudogene	1
	ndhE	0	0	1

	ndhF	0	0	1
	ndhH	0	0	1
	ndhI	0	pseudogene	1
	ndhJ	0	pseudogene	1
	ndhK	0	pseudogene	1
Total		0	31	43

Supplementary Table 30 | The read number of plastid protein-coding genes in five transcriptomes during different periods of *G. elata* growth.

Gene	Protocorms (A)	Juvenile tubers (B)	Immature tubers (C)	Mature tubers (D)	Scapes (E)
accD	14	45	30	13	8
clpP	2882	8033	8502	5046	1841
matK	1	3	2	2	0
rpl14	148	407	318	142	75
rpl16	924	2170	2365	969	561
rpl2	24	52	60	30	6
rpl20	111	1029	229	52	38
rps11	171	309	305	210	92
rps12	387	497	1218	435	120
rps18	12	33	38	10	2
rps19	0	1	0	2	0
rps2	0	4	6	2	1
rps3	4	6	6	1	0
rps4	3	16	83	81	0
rps7	192	458	320	169	68
rps8	72	210	155	67	7
ycf1	967	2296	2598	1589	87
ycf2	30	90	218	118	7

Supplementary Table 31 | Expression of nuclear genes coding polypeptide subunits of protein complexes of the photosynthetic apparatus.

Gene ID	IPR	Protocorms (A)	Juvenile tubers (B)	Immature tubers (C)	Mature tubers (D)	Scapes (E)
Gel0123051	ATPase, F1/V1/A1 complex, alpha/beta subunit, nucleotide-binding domain	355.2040	498.7467	232.8501	204.2964	195.5822
Gel0178051	ATPase, V1/A1 complex, subunit E	145.7808	173.8212	136.3700	128.1211	130.7405
Gel0169018	ATPase, F1/V1/A1 complex, alpha/beta subunit, nucleotide-binding domain	0.0000	0.3384	0.0448	0.0373	0.1633
Gel0000470	ATPase, V1 complex, subunit C	32.9514	38.4136	36.2313	38.1142	39.7136
Gel0129020	ATPase, F0 complex, subunit G, mitochondrial	293.8164	366.1572	299.9426	287.2306	152.6925
Gel0277017	Vacuolar (H+)-ATPase G subunit	226.9196	298.7174	177.9376	183.1634	220.1739
Gel0030032	ATPase, V1 complex, subunit H	60.7739	59.8468	58.6665	51.9680	41.0230
Gel0004259	Photosystem II PsbQ, oxygen evolving complex	29.6599	35.3208	36.9687	32.7119	76.6176
Gel0114088	ATPase, V1 complex, subunit D	60.5673	56.4176	77.8386	80.4664	53.9667
Gel0064240	Photosystem II Psp29, biogenesis	37.1920	34.9129	26.9458	29.0619	37.7377
Gel0021047	Photosystem II PsbQ, oxygen evolving complex	4.6660	4.3990	22.4109	51.7141	21.3447
Gel0050082	Ran-interacting Mog1 protein	59.3422	66.1692	55.7481	46.0174	54.8429

Supplementary Table 32 | GO term enrichment results of significantly expanded gene families in *G. elata*. (MF: molecular function; BP: biological process; CC: cellular component).

GO ID	GO Term	GO Class	Adjusted p-value*
GO:0008970	phosphatidylcholine 1-acylhydrolase activity	MF	1.40E-56
GO:0047714	galactolipase activity	MF	1.40E-56
GO:0004806	triglyceride lipase activity	MF	1.66E-47
GO:0016788	hydrolase activity, acting on ester bonds	MF	4.98E-40
GO:0006629	lipid metabolic process	BP	2.44E-32
GO:0018024	histone-lysine N-methyltransferase activity	MF	1.36E-20
GO:0042393	histone binding	MF	6.03E-20
GO:0034968	histone lysine methylation	BP	1.26E-18
GO:0018022	peptidyl-lysine methylation	BP	1.26E-18
GO:0016787	hydrolase activity	MF	2.47E-18
GO:0008168	methyltransferase activity	MF	1.01E-14
GO:0004970	ionotropic glutamate receptor activity	MF	4.71E-14
GO:0005234	extracellular-glutamate-gated ion channel activity	MF	4.71E-14
GO:0022824	transmitter-gated ion channel activity	MF	4.71E-14
GO:0030594	neurotransmitter receptor activity	MF	4.71E-14
GO:0008474	palmitoyl-(protein) hydrolase activity	MF	1.08E-13
GO:0030288	outer membrane-bounded periplasmic space	CC	1.52E-13
GO:0006874	cellular calcium ion homeostasis	BP	4.08E-13
GO:0005217	intracellular ligand-gated ion channel activity	MF	2.16E-12
GO:0004555	alpha,alpha-trehalase activity	MF	5.02E-11
GO:0005993	trehalose catabolic process	BP	5.02E-11
GO:0044710	single-organism metabolic process	BP	1.05E-09
GO:0022843	voltage-gated cation channel activity	MF	2.82E-09
GO:0003824	catalytic activity	MF	2.10E-08
GO:0015171	amino acid transmembrane transporter activity	MF	2.54E-08
GO:0006865	amino acid transport	BP	4.49E-08
GO:0017110	nucleoside-diphosphatase activity	MF	6.12E-08
GO:0010429	methyl-CpNpN binding	MF	7.56E-07
GO:0010428	methyl-CpNpG binding	MF	7.56E-07
GO:0004553	hydrolase activity, hydrolyzing O-glycosyl compounds	MF	1.08E-06
GO:0015075	ion transmembrane transporter activity	MF	1.74E-06
GO:0044444	cytoplasmic part	CC	2.88E-06
GO:0043231	intracellular membrane-bounded organelle	CC	7.40E-06
GO:0008327	methyl-CpG binding	MF	1.78E-05
GO:0043933	macromolecular complex subunit organization	BP	1.89E-05
GO:0005720	nuclear heterochromatin	CC	3.76E-05

GO:0040029	regulation of gene expression, epigenetic	BP	0.000229231
GO:0005215	transporter activity	MF	0.000245419
GO:0044699	single-organism process	BP	0.000527723
GO:0009294	DNA mediated transformation	BP	0.000683267
GO:0019618	protocatechuate catabolic process, ortho-cleavage	BP	0.000824809
GO:0019615	catechol catabolic process, ortho-cleavage	BP	0.000824809
GO:0009846	pollen germination	BP	0.001153714
GO:0003988	acetyl-CoA C-acyltransferase activity	MF	0.003697142
GO:0044238	primary metabolic process	BP	0.00374833
GO:0010216	maintenance of DNA methylation	BP	0.004956284
GO:0004930	G-protein coupled receptor activity	MF	0.004956284
GO:0005975	carbohydrate metabolic process	BP	0.006410658
GO:0005773	vacuole	CC	0.006881767
GO:0008324	cation transmembrane transporter activity	MF	0.011212088
GO:0008270	zinc ion binding	MF	0.013997138
GO:0006811	ion transport	BP	0.014242486
GO:0046974	histone methyltransferase activity (H3-K9 specific)	MF	0.022522408
GO:0006464	cellular protein modification process	BP	0.023810159
GO:0071704	organic substance metabolic process	BP	0.023824142
GO:0051536	iron-sulfur cluster binding	MF	0.030114669
GO:0044464	cell part	CC	0.03553302
GO:0030308	negative regulation of cell growth	BP	0.038550375
GO:0004965	G-protein coupled GABA receptor activity	MF	0.038550375

GO:0005275	amine transmembrane transporter activity	MF	0.038550375
GO:0030312	external encapsulating structure	CC	0.040001459
GO:0046686	response to cadmium ion	BP	0.040469663
GO:0007186	G-protein coupled receptor signaling pathway	BP	0.044578266
GO:0044712	single-organism catabolic process	BP	0.045746357

*Fisher’s exact test, corrected by false discovery rate.

Supplementary Table 33 | Over-represented Pfam domains in *G. elata* genome. The table depicts the comparison of percentages and absolute numbers of respective over-represented domains in *G. elata* (Fisher's exact test, p<0.01, adjusted for multiple tests through false discovery rate)

**Pfam**	**Gel***	**Peq†**	**Dof‡**	**Aco§**	**Osa‖**	**Ath¶**	**Vvi#**	**Adjusted p-value**
PF01204:Trehalase	7	1	3	1	1	1	1	0.02396
PF00547:Urease_gamma	6	1	1	1	1	1	1	0.042241

***** Gel: *G. elata*; **†** Peq: *P. equestris*; **‡** Dof: *D. officinale*; **§** Aco: *A. comosus*; **‖** Osa: *O. sativa*; **¶** Ath: *A. thaliana*; # Vvi: *V. vinifera*.

Supplementary Table 34 | Expression levels of protein coding genes of mitochondrial genomes.

No.	Gene name	Copy No.	Transcripted level in mature tuber			
			Epidermis	Cortex	Parenchymal cell A	Parenchymal cell B
1	atp1	1	2.835±0.377	7.515±0.926	1.596±0.045	1.691±0.244
2	atp4	2	0.445±0.055	10.664±0.775	1.310±0.144	2.131±0.281
3	atp6	1	1.976±0.202	7.255±1.044	3.300±0.512	2.019±0.315
4	atp8	1	2.103±0.310	2.216±0.613	2.655±0.578	0.956±0.197
5	atp9	1	0.625±0.045	2.422±0.285	1.551±0.231	2.336±0.203
6	ccmB	1	0.884±0.101	7.934±0.684	2.786±0.341	2.467±0.285
7	ccmC	1	0.944±0.101	9.510±1.216	1.443±0.087	3.120±1.126
8	ccmFc	1	7.872±1.036	758.139±91.227	21.071±5.635	1.731±0.609
9	ccmFn	1	1.789±0.215	18.611±2.059	1.952±0.087	1.650±0.250
10	cob	1	4.587±0.632	26.148±2.963	5.802±0.639	1.887±0.154
11	cox1	1	0.953±0.080	6.165±0.525	1.135±0.074	2.579±0.238
12	cox2	1	6.235±0.686	20.178±2.379	6.058±0.560	2.094±0.144
13	cox3	1	3.192±0.280	6.405±0.940	3.956±0.749	1.734±0.337
14	matR	1	2.431±0.278	12.848±1.661	1.435±0.045	1.588±0.220
15	mttB	1	1.021±0.230	6.682±0.470	1.579±0.068	1.125±0.472
16	nad3	1	0.870±0.136	14.670±1.441	2.899±0.232	2.104±0.203
17	nad4	1	2.949±0.512	5.792±0.873	2.011±0.307	1.422±0.267
18	nad4L	1	0.631±0.072	4.987±0.789	1.714±0.381	2.171±0.268
19	nad5	1	2.775±0.281	2.634±0.680	2.455±0.471	1.395±0.257
20	nad6	1	0.384±0.068	24.166±4.252	1.575±0.973	1.708±0.170
21	nad7	1	1.734±0.490	3.595±0.697	1.887±0.318	2.847±0.273
22	nad9	1	0.464±0.050	11.119±1.313	1.380±0.099	2.345±0.711
23	rpl2	1	3.510±0.622	21.616±1.937	3.227±0.362	1.781±0.303
24	rpl5	1	3.596±0.410	17.973±1.286	3.356±0.452	1.906±0.345
25	rpl16	1	0.611±0.087	7.332±0.397	1.179±0.138	2.684±0.329
26	rps1	1	0.683±0.082	0.103±0.019	0.923±0.130	1.511±0.213
27	rps2	1	1.813±0.167	21.211±3.770	1.125±0.301	1.669±0.998
28	rps3	1	3.724±0.346	49.055±10.843	2.284±0.333	1.912±0.214
29	rsp4	1	2.995±0.244	35.165±7.189	1.424±0.365	2.231±0.222
30	rps7	1	1.137±0.270	6.925±1.271	1.881±0.268	1.771±0.097
31	rps10	1	1.787±0.107	0.406±0.054	1.827±0.241	1.896±0.283
32	rps11	1	0.388±0.058	0.518±0.032	1.663±0.208	2.760±0.209
33	rps12	1	4.219±2.557	4.928±1.132	2.150±0.691	2.105±0.818
34	rps13	1	3.096±1.485	26.414±9.940	0.391±0.0525	1.973±0.327
35	rps14	1	4.465±0.517	13.195±1.038	4.235±0.677	1.437±0.488
36	rps19	1	2.006±0.304	9.275±1.546	1.223±0.189	1.052±0.222

- : not detected; * : $2^{-\triangle Ct}$.

Supplementary Table 35 | The relative intensity of *p*-HA and S-(*p*-PA)-glutathione (cps) in tuber of *G. elata* and *A. mellea* hypha using UPLC-PDA-ESI-Q-TOF-MS^{E}.

**Samples**	**Source**	***p*-HA* (cps)**	***S*-(*p*-PA)-glutathione (cps)**
tuber		0.3390±0.046	0.7948±0.0206
hypha	tuber	-	0.0021±0.0032
	wood	-	-
	potato dextrose	-	-
	agar medium	-	-

* Detection at 270 nm. - : undetected.

Supplementary Table 36 | Community richness and diversity indices of bacteria associated with different periods of *G. elata* growth, with or without *A. mellea* partner.

Sample name	Observed species	chao1[70]	ACE[71,72]	shannon[73]	simpson[74,75]	Goods coverage[76,77]	PD whole tree[78]
protocorm	41.33±2.08	50.36±3.24	49.58±1.03	3.26±0.12	0.86±0.02	1.00±0.00	5.69±0.31
juvenile tuber	667.33±60.72	782.79±62.60	799.68±44.64	5.56±0.28	0.94±0.01	1.00±0.00	54.50±3.51
immature tuber	665.33±102.92	771.05±144.35	783.31±125.29	6.03±0.56	0.96±0.01	1.00±0.00	51.27±6.05
mature tuber	624.33±28.73	748.22±75.41	732.84±47.77	5.06±0.35	0.90±0.03	1.00±0.00	48.22±1.61

Reads filtering: (1) removing reads containing more than 10% of unknown nucleotides; (2) removing reads containing less than 80% of bases with quality (Q-value) > 20. The filtered reads were then assembled into tags according to overlap between paired-end reads with more than 10-bp overlap and less than 2% mismatch. The high-quality sequences were clustered into operational taxonomic units (OTUs) defined at 97%. Chao1 estimator[70] and ACE estimator[71, 72] mainly revealed the microbial community richness; Shannon index[73] and Simpson index[74, 75] mainly suggested the microbial community diversity; Goods coverage[76, 77] showed the sequencing depth and coverage; PD whole tree[78] uncovered the phylogenetic diversity. Protocorm: *G. elata* lives without *A. mellea*; juvenile, immature, mature tubers: *G. elata* lives with *A. mellea*. Each value is the mean of 3 replicates (±SD).

Supplementary Table 37 | Community richness and diversity indices of fungi associated with different periods of *G. elata* growth with or without *A. mellea* partner.

Sample name	Observed species	chao1[70]	ACE[71,72]	shannon[73]	simpson[74,75]	Goods coverage[76,77]	PD whole tree[78]
protocorm	15.67±2.89	16.94±4.38	17.19±2.98	0.57±0.23	0.19±0.08	1.00±0.00	8.40±1.07
juvenile tuber	69.33±17.90	78.13±17.88	78.92±20.06	0.91±0.76	0.27±0.07	1.00±0.00	29.31±3.97
immature tuber	157.33±36.25	176.38±27.86	176.47±26.85	2.46±1.74	0.56±0.31	1.00±0.00	59.87±8.53
mature tuber	145.33±105.95	157.31±115.75	161.28±118.55	1.59±1.10	0.39±0.29	1.00±0.00	51.45±30.31

Reads filtering: (1) removing reads containing more than 10% of unknown nucleotides; (2) removing reads containing less than 80% of bases with quality (Q-value) > 20. The filtered reads were then assembled into tags according to overlap between paired-end reads with more than 10-bp overlap and less than 2% mismatch. The high-quality sequences were clustered into operational taxonomic units (OTUs) defined at 97%. Chao1 estimator[70] and ACE estimator[71, 72] mainly revealed the microbial community richness; Shannon index[73] and Simpson index[74, 75] mainly suggested the microbial community diversity; Goods coverage[76, 77] showed the sequencing depth and coverage; PD whole tree[78] uncovered the phylogenetic diversity. Protocorm: *G. elata* lives without *A. mellea*; juvenile, immature, mature tubers: *G. elata* lives with *A. mellea*. Each value is the mean of 3 replicates (±SD).

Supplementary Table 38 | Number of recognition signal related genes of the *G. elata* genome compared with those of *P. equestris*, *D. officinale*, *A. comosus* and *A. thaliana.*

**Class**	**Gene name**	**Gel[*]**	**Peq[†]**	**Dof[‡]**	**Aco[§]**	**Ath‖**	**P1[¶]**	**P2[#]**	**P3[☆]**	**P4[**]**	**P5[††]**
strigolactone	**CCD7**	5	3	4	1	1	**0.0331**	**0.0453**	0.0881	0.1583	0.0596
	CCD8	2	1	0	1	1	0.1414	0.5714	0.5728	0.1301	1.0000
	**PDR**	21	20	15	14	15	**0.0065**	**0.0411**	**0.0263**	**0.0104**	0.3746
chitooligosaccharides and lipochitooligosaccharides	LysM	26	34	32	51	53	1.0000	0.1695	0.2032	0.2116	0.0001
	DMI2	22	37	25	28	27	0.4589	0.5647	0.7741	0.4340	1.0000
	CASTOR/POLLUX	1	2	1	2	1	1.0000	1.0000	1.0000	1.0000	1.0000
	**DMI3**	10	3	5	4	8	**0.0051**	0.2346	**0.0285**	**0.0025**	1.0000
	IPD3	1	3	2	1	1	1.0000	1.0000	1.0000	1.0000	0.4930
	**RAM1**	25	24	25	18	18	**0.0116**	**0.0287**	**0.0295**	**0.0259**	0.1719
	RAM2	2	2	2	3	3	0.6750	1.0000	1.0000	0.6233	0.5571

*. Gel: *Gastrodia elata*; †. Peq: *Phalaenopsis equestris*; ‡. Dof: *Dendrobium officinale*; §. Aco: *Ananas comosus*; ‖. Ath: *Arabidopsis thaliana*; ¶. P1: Fisher's exact test of gene number in *G. elata* genome compared to *P. equestris*, *D. officinale*, *A. comosus* and *A. thaliana*; #. P2: Fisher's exact test of gene number in *G. elata* genome compared to *A. thaliana*; ☆. P3: Fisher's exact test of gene number in *G. elata* genome compared to *A. comosus*; **. P4: Fisher's exact test of gene number in *G. elata* genome compared to *P. equestris* and *D. officinale*; ††. P5: Fisher's exact test of gene number in *G. elata*, *P. equestris* and *D. officinale* genome compared to *A. comosus* and *A. thaliana*. The contracted/expanded gene sets were highlighted in bold format ($P<0.05$).

Supplementary Table 39 | Transcriptional level of GHs and transporters genes in different tuber tissues as assessed by real-time PCR analysis (±SE, n=6).

Function	Gene	Epidermis	Cortex	Parenchymal cell A	Parenchymal cell B
AG	Gel0076061	6.219±0.265	9.548±2.053	2.876±0.314	1.469±0.300
	Gel0105077	13.308±1.145	2432.942±268.002	63.340±9.639	30.576±6.719
xynA	Gel0131048	0.755±0.091	150.560±55.887	16.431±3.556	1.039±0.292
Other GHs	Gel0220010	0.942±0.051	5.800±1.002	10.644±1.095	1.146±0.087
	Gel0076070	6.735±0.610	22.529±5.133	71.846±3.729	4.988±1.341
	Gel0113034	2.599±0.128	10.422±2.688	16.559±1.831	1.399±0.232
	Gel0090109	12.736±1.054	6.607±2.647	13.519±1.239	3.357±0.740
	Gel0044207	13.062±4.707	24706.958±12597.856	29.042±11.118	5.365±3.667
	Gel0099111	0.004±0.001	1.755±0.930	0.035±0.018	0.057±0.025
	Gel0097007	0.808±0.110	23.767±6.035	1.829±0.300	2.712±0.277
NRT	Gel0023221	2.903±0.133	48.608±7.621	44.383±5.271	4.505±0.555
	Gel0023223	0.915±0.207	245.009±87.385	3.602±0.644	5.152±0.963
GAT	Gel0126003	1.523±0.094	8.745±1.006	0.000±0.000	1.444±0.213
CAT	Gel0178092	41.423±2.952	412.900±46.475	14.736±3.393	14.482±2.549
ANT	Gel0059241	4.574±0.517	928.809±396.271	2.373±0.861	3.497±0.715
acid	Gel0112041	1.312±0.078	37.492±6.786	1.585±0.129	2.916±0.273
phosphatase	Gel0173004	0.043±0.004	15.969±4.360	0.772±0.122	0.714±0.088
H+-ATPase	Gel0087098	1.937±0.205	15.361±12.940	46.723±8.732	4.531±0.595
KC1	Gel0107001	17.269±14.744	196.524±84.244	1.242±0.329	17.085±11.426
KT1	Gel0099067	0.405±0.022	65.399±1.830	2.965±0.620	0.528±0.072
SWEET	Gel0044071	0.005±0.000	2.519±0.443	0.378±0.042	0.302±0.030
	Gel0115006	0.518±0.025	14.032±2.123	1.270±0.232	1.482±0.153
	Gel0062102	0.307±0.039	2.758±0.474	0.300±0.039	0.711±0.051
	Gel0066042	55.353±49.856	6628.471±6584.495	109.853±69.943	61.540±58.856

Supplementary Table 40 | Analysis glycoside hydrolase genes of the *G. elata* genome compared with those in the genome of *P. equestris, D. officinale, A. comosus* and *A. thaliana.*

Class	Gene name	Gel[*]	Peq[†]	Dof[‡]	Aco[§]	Ath[‖]	P1[¶]	P2[#]	P3[☆]	P4[**]	P5[††]
Glycoside hydrolases	alpha-Glucosidases	3	1	2	1	1	0.0846	0.3117	0.3130	0.1333	0.4930
	**beta-Glucosidase**	10	15	13	11	7	0.2273	**0.0179**	0.3653	0.8512	**0.0102**
	beta-xylosidase	5	6	4	7	6	0.5782	0.7674	1.0000	0.3525	0.4455
	alpha-mannosedases I	3	2	2	3	5	0.4487	1.0000	0.6959	0.1954	0.2969
	alpha-D-mannosedases (EC:3.2.1.24)	2	6	6	2	6	0.7591	0.4848	1.0000	0.7490	0.8310
	alpha-D-mannosidase II (EC:3.2.1.114)	1	2	1	1	0	0.5215	0.4090	1.0000	1.0000	0.6546
	beta-mannosidase	1	2	0	0	1	0.4455	1.0000	0.4124	0.5362	1.0000
	alpha-Galactosidase	1	3	2	4	1	1.0000	1.0000	0.6549	1.0000	0.7611
	beta-galactosidase	7	11	11	9	13	1.0000	0.6556	1.0000	0.8252	0.5701
	**endo-β-1,4-D-xylanase**	11	6	4	6	0	**0.0005**	**5.36E-05**	0.0812	**0.0028**	**0.0773**
	alpha-N-arabinofuranosidase	1	2	2	3	0	1.0000	0.4090	0.6476	1.0000	1.0000

*. Gel: *Gastrodia elata*; †. Peq: *Phalaenopsis equestris*; ‡. Dof: *Dendrobium officinale*; §. Aco: *Ananas comosus*; ‖. Ath: *Arabidopsis thaliana*; ¶. P1: Fisher's exact test of gene number in *G. elata* genome compared to *P. equestris*, *D. officinale*, *A. comosus* and *A. thaliana*; #. P2: Fisher's exact test of gene number in *G. elata* genome compared to *A. thaliana*; ☆. P3: Fisher's exact test of gene number in *G. elata* genome compared to *A. comosus*; **. P4: Fisher's exact test of gene number in *G. elata* genome compared to *P. equestris* and *D. officinale*; ††. P5: Fisher's exact test of gene number in *G. elata, P. equestris* and *D. officinale* genome compared to *A. comosus* and *A. thaliana*. The contracted/expanded gene sets were highlighted in bold format (P<0.05).

Supplementary Table 41 | Analysis amino acid transporter and arginine biosynthesis related genes of the *G. elata* genome compared with those in the genome of *P. equestris*, *D. officinale*, *A. comosus* and *A. thaliana*..

**Class**	**Gene name**	**Gel[*]**	**Peq[†]**	**Dof[‡]**	**Aco[§]**	**Ath[‖]**	**P1[¶]**	**P2[#]**	**P3[☆]**	**P4[**]**	**P5[††]**
amino acid transporters	AAP	7	12	11	8	7	0.6655	0.5892	0.7944	1.0000	0.4488
	ANT	5	3	5	2	4	0.1687	0.5008	0.1330	0.1854	0.6402
	AUXIN (_like)	3	3	4	3	3	0.4735	0.6938	0.6959	0.7039	1.0000
	BAT1	2	5	5	3	1	1.0000	0.5714	1.0000	1.0000	0.3105
	CAT	7	9	1	9	9	0.3196	0.8051	1.0000	0.0806	0.1664
	LHT	2	6	6	3	8	0.5668	0.2150	1.0000	0.7490	0.6842
	PROT	3	4	7	2	0	0.4735	0.0684	0.4092	1.0000	0.0375
	Total	29	42	39	30	32	0.2229	0.2996	0.2348	0.3611	0.3914
arginine biosynthesis	ArgB	5	6	6	3	4	0.3651	0.5008	0.2875	0.5604	0.4044
	ArgD	6	7	7	11	8	0.8146	1.0000	0.8064	0.4252	0.2527
	ArgE	4	5	6	6	8	1.0000	0.7717	1.0000	0.7571	0.34555
	ArgF	2	2	2	2	2	0.6363	1.0000	1.0000	0.6233	1.0000
	ArgG	2	2	1	2	1	0.3018	0.5714	1.0000	0.3165	1.0000
	ArgH	1	1	0	1	1	0.4455	1.0000	1.0000	0.4008	0.6487
	**ArgJ**	3	1	1	1	1	**0.0478**	0.0907	0.1478	0.1052	0.4528
**Ureases**	**Ureases**	9	2	2	1	1	**3.89E-05**	**0.0020**	**0.0022**	**0.0004**	**0.0604**

*. Gel: *Gastrodia elata*; †. Peq: *Phalaenopsis equestris*; ‡. Dof: *Dendrobium officinale*; §. Aco: *Ananas comosus*; ‖. Ath: *Arabidopsis thaliana*; ¶. P1: Fisher's exact test of gene number in *G. elata* genome compared to *P. equestris*, *D. officinale*, *A. comosus* and *A. thaliana*; #. P2: Fisher's exact test of gene number in *G. elata* genome compared to *A. thaliana*; ☆. P3： Fisher's exact test of gene number in *G. elata* genome compared to *A. comosus*; **. P4: Fisher's exact test of gene number in *G. elata* genome compared to *P. equestris* and *D. officinale*; ††. P5: Fisher's exact test of gene number in *G. elata*, *P. equestris* and *D. officinale* genome compared to *A. comosus* and *A. thaliana*. The contracted/expanded gene sets were highlighted in bold format (P<0.05).

Supplementary Table 42 | Voucher of *G. elata* in plastid genome.

**Species**	**Voucher**	**Locality**	**Herbarium**
G. elata	Xiaohua Jin et al. 13195	Linzhi, Tibet	PE
G. elata	Xiaohua Jin 16738	Zhaotong, Yunnan	PE
G. elata	Xiaohua Jin 16738	Zhaotong, Yunnan	PE

Supplementary Table 43 | Detail of genes in mitochondrial genome.

Sequence Name	type	gene
ge7	coding	atp1
ge7	coding	atp4
ge17	coding	atp4
ge5	coding	atp6
ge7	coding	atp8
ge8	coding	atp9
ge6	coding	ccmB
ge12	coding	ccmC
ge5	coding	ccmFc
ge7	coding	ccmFn
ge18	coding	cob
ge10	coding	cox1
ge19	coding	cox2
ge17	coding	cox3
ge19	coding	matR
ge7	coding	mttB
ge7	coding	nad2
ge7	coding	nad3
ge3	coding	nad4
ge17	coding	nad4L
ge15	coding	nad5
ge5	coding	nad6
ge7	coding	nad7
ge19	coding	nad9
ge7	coding	rpl2
ge18	coding	rpl5
ge7	coding	rpl16
ge7	coding	rps1
ge7	coding	rps2
ge7	coding	rps3
ge8	coding	rps7
ge5	coding	rps10
ge3	coding	rps11
ge7	coding	rps12
ge3	coding	rps13
ge18	coding	rps14
ge7	coding	rps19
ge2	rrn	rrn5
ge4	rrn	rrn5
ge4	rrn	rrn18

ge19	trn	trnC-GCA
ge7	trn	trnE-TTC
ge19	trn	trnF
ge17	trn	trnF-AAA
ge4	trn	trnfM
ge6	trn	trnfM
ge7	trn	trnH-GTG
ge1	trn	trnI-AAT
ge17	trn	trnI-cp
ge4	trn	trnK-UUU
ge18	trn	trnM-CAT
ge18	trn	trnM-CAT_b
ge1	trn	trnQ-TTG
ge8	trn	trnQ-TTG
ge4	trn	trnS-GCT
ge18	trn	trnS-GGA
ge7	trn	trnV-CAC
ge18	trn	trnV-CAC
ge7	trn	trnW-CCA
ge19	trn	trnY-AUA
ge3	trn	trnY-GTA
ge7	trn	trnY-GTA
ge3	misc_feature	ccmFn fragment
ge4	misc_feature	atp6 fragment
ge6	misc_feature	atp4 fragment
ge7	misc_feature	rps1 fragment
ge7	misc_feature	atp9 fragment
ge17	misc_feature	nad1 fragment
ge19	misc_feature	nad1 fragment
ge3	misc_feature	nad1_fragment
ge4	misc_feature	nad1_fragment
ge3	misc_feature	nad1_fragment
ge7	misc_feature	nad5_fragment

Supplementary Table 44 | Transcriptional levels of glycoside hydrolases (GHs) genes at different development stages.

Family	Gene ID	A	B	C	D	E
GH1	Gel0022031	18.6421	18.9434	20.8052	22.7772	15.6837
GH1	Gel0063087	121.4216	102.8922	119.7358	46.7981	13.0185
GH1	Gel0022035	19.1303	19.2866	13.4062	14.1162	10.2946
GH1	Gel0010001	11.1708	11.9135	15.3740	15.7980	7.4898
GH1	Gel0022001	19.2935	22.3593	22.8897	24.1054	22.3034
GH1	Gel0112120	82.3164	94.5906	78.4471	74.2689	87.0102
GH1	Gel0235019	1230.6943	2091.0357	769.4116	728.9982	103.3898
GH1	Gel0097007	264.0586	472.4123	8.6207	5.5417	60.2072
GH1	Gel0013068	264.7693	204.8106	153.9987	119.4431	44.5860
GH1	Gel0211001	232.0339	253.0363	360.3161	181.5652	8.0054
GH1	Gel0097005	132.3019	62.6138	11.0730	17.6486	1079.3328
GH1	Gel0215025	18.7221	14.5345	4.0238	6.6430	73.0410
GH10	Gel0131044	0.0000	0.0000	0.0000	0.0000	0.0000
GH10	Gel0131042	19.0394	15.0097	18.1262	14.5567	14.2067
GH10	Gel0014014	1.4455	2.3828	0.0732	0.0371	0.2861
GH10	Gel0131046	0.0000	0.0000	0.0000	0.0000	0.0000
GH10	Gel0156037	5.3814	4.7116	4.8676	3.2063	18.0626
GH10	Gel0131047	0.0000	0.0000	0.0000	0.0000	0.0000
GH10	Gel0129018	18.2522	16.2113	5.5352	6.1010	15.9215
GH10	Gel0131043	0.2230	0.1198	0.0433	0.0468	0.0770
GH10	Gel0131050	0.0000	0.0000	0.0000	0.0000	0.0000
GH10	Gel0131048	0.3372	0.1898	0.3375	0.9250	0.4047
GH10	Gel0036024	12.4219	13.5260	13.2925	11.6660	8.7756
GH100	Gel0245011	35.2592	33.0788	39.0060	41.1108	40.1408
GH100	Gel0045068	0.1299	0.1806	0.1358	0.1372	1.4534
GH100	Gel0017064	51.8777	44.4482	48.3007	65.6890	79.6246
GH100	Gel0017206	59.3937	78.5916	75.5804	76.9221	97.8251
GH100	Gel0060032	1.0244	1.2693	0.7194	0.5167	1.2417
GH100	Gel0109012	7.4710	10.5711	25.3674	9.4533	16.9375
GH100	Gel0060031	8.2811	12.8468	8.8673	10.6800	15.8115
GH116	Gel0059147	46.2824	44.8750	59.4581	66.0087	66.7847
GH116	Gel0059031	80.2903	41.0083	18.4440	15.5868	26.3059
GH13	Gel0023245	120.0644	113.8402	171.8483	239.9419	33.4923
GH14	Gel0016229	18.4960	17.3238	19.8968	18.8359	12.4106
GH14	Gel0017009	4.7772	4.7275	3.5347	3.0484	3.8543
GH14	Gel0059253	20.5915	22.7648	23.3074	25.1742	19.6093
GH14	Gel0059252	2.1142	3.3254	4.2733	3.9674	1.8888
GH14	Gel0059173	131.4419	181.1191	139.3417	301.6482	748.5592
GH14	Gel0148072	32.6136	55.0323	23.6243	24.7733	259.4538
GH16	Gel0102030	0.4589	0.6548	0.1026	0.2716	0.0458

GH16	Gel0050177	28.1590	29.2938	33.3126	14.5720	5.1478
GH16	Gel0231010	7.8224	7.0702	5.4925	57.5748	4.8313
GH16	Gel0139118	97.7877	152.8983	168.2691	106.6294	10.0763
GH16	Gel0087023	67.0316	72.1704	33.9591	161.1296	176.9102
GH16	Gel0042163	30.6442	37.5257	43.5109	41.4533	59.5108
GH16	Gel0004144	14.0260	19.0956	20.7489	15.2422	6.2378
GH16	Gel0125001	211.1583	227.8879	149.3834	127.0958	92.3750
GH16	Gel0147003	1018.6633	1084.2424	6645.8127	5715.4948	2486.7008
GH16	Gel0071094	0.0000	0.0000	0.0176	0.0992	0.9685
GH16	Gel0019030	57.3322	80.2419	427.9532	275.1264	233.7739
GH16	Gel0042079	0.0625	0.0000	0.0204	0.0187	1.4873
GH16	Gel0030052	2.6185	0.3106	0.0271	0.0000	0.0254
GH16	Gel0143067	1296.8296	1539.1762	2382.9617	4099.5597	1435.2473
GH16	Gel0025015	169.1558	130.6792	159.0923	66.4665	12.2238
GH17	Gel0064099	15.1984	14.5532	20.6874	21.3096	6.3066
GH17	Gel0044065	24.6758	26.5537	3.6296	4.7878	2.1639
GH17	Gel0013134	59.8635	48.6306	50.8674	51.5955	54.4154
GH17	Gel0001279	60.9219	64.0707	59.0773	60.7169	51.1597
GH17	Gel0110090	12.1682	22.6441	25.6875	19.5034	21.8559
GH17	Gel0094048	58.9483	65.9260	15.4784	17.5579	145.3522
GH17	Gel0084056	1.5760	1.2556	0.2785	0.1162	0.3291
GH17	Gel0079088	9.9971	13.5629	7.8532	6.6990	2.7347
GH17	Gel0019221	17.0258	15.9913	6.7729	6.7715	2.0145
GH17	Gel00731961	20.6576	26.2388	21.4160	21.4523	27.0549
GH17	Gel0179049	1.2935	1.0777	0.3981	0.3584	1.9606
GH17	Gel0012186	0.2047	0.1273	0.0704	0.0811	0.4884
GH17	Gel0008216	52.1668	51.5486	36.2776	30.4886	98.0404
GH17	Gel0055009	78.1285	70.5842	85.2073	82.8862	42.8157
GH17	Gel0080143	1.0640	1.0014	0.4111	0.6858	0.4698
GH17	Gel0155029	8.9445	8.1326	8.7803	16.4157	19.6006
GH17	Gel01580312	37.9188	34.2928	23.1905	24.1028	113.1060
GH17	Gel0100087	1.5872	2.2716	0.3972	0.9672	6.2535
GH17	Gel0128012	94.6486	140.9350	25.8466	13.2075	1.1021
GH17	Gel0152050	15.5386	14.9276	13.6549	14.5579	19.8546
GH17	Gel0235001	19.5690	19.8948	14.6914	13.4508	59.8183
GH17	Gel0071073	14.6543	20.2032	6.7892	7.0504	12.3920
GH17	Gel0257024	48.5413	39.5001	35.3918	51.5204	147.1867
GH17	Gel0033067	3.5969	3.7694	2.9270	3.3111	4.3434
GH18	Gel0000135	0.5695	0.3508	0.6814	0.0807	0.2559
GH18	Gel0095018	8.1401	10.1117	5.1339	5.4061	3.8368
GH18	Gel0001049	8.3075	10.2874	0.0592	0.0181	0.0000
GH18	Gel0001059	92.1738	127.9166	6.5010	16.1284	67.6593
GH18	Gel0049055	9.9010	8.8867	8.9082	9.1729	13.2811
GH18	Gel0188057	89.9792	173.8383	24.9749	24.8471	2.1785

GH19	Gel0185018	214.0247	270.7996	379.0981	397.0433	1224.4889
GH19	Gel0053092	0.5958	0.2773	0.1114	0.8160	0.2003
GH19	Gel0108114	13.1108	41.6675	0.2561	0.3135	1.6173
GH2	Gel0080054	17.2469	21.2109	13.7861	14.7143	14.6558
GH2	Gel0005186	28.7636	35.4745	18.9764	8.6740	12.3770
GH2	Gel0005172	19.0589	17.7692	32.1071	24.3698	9.1919
GH20	Gel0060117	21.4497	18.4912	8.8116	9.1833	18.8236
GH20	Gel0161010	2.6397	2.7783	0.7193	1.4075	5.3213
GH20	Gel0119031	109.5832	55.6616	9.6040	12.6804	29.0310
GH27	Gel0213012	37.1645	43.8814	25.3445	20.5651	9.7858
GH27	Gel0015122	24.4226	15.1753	22.8553	20.3390	15.9242
GH27	Gel0016112	210.0097	133.3571	488.2929	975.3807	1374.8648
GH28	Gel0068074	14.1040	14.4520	11.5855	8.3620	7.9916
GH28	Gel0077005	0.6122	0.7315	0.0394	0.1168	0.5664
GH28	Gel0019093	25.3152	26.2029	22.6031	24.0615	47.1847
GH28	Gel0103033	2.3041	2.0735	2.5508	4.8071	1.5671
GH28	Gel0187059	5.1619	5.4533	0.7384	1.0241	1.0274
GH28	Gel0103024	1.1006	1.1998	0.9115	0.9459	1.6421
GH28	Gel0170042	50.0973	52.4164	54.8100	51.2108	60.7511
GH28	Gel0233005	37.7973	32.8873	39.4017	35.1590	37.3164
GH28	Gel0021099	0.4516	0.5193	0.1348	0.0566	12.0778
GH28	Gel0112007	0.0826	0.0307	0.0000	0.0000	0.0136
GH28	Gel0000001	0.0430	0.0416	0.2379	0.1904	53.4085
GH28	Gel0233007	29.3422	28.0562	33.6440	27.4500	27.4656
GH28	Gel0040017	12.7959	13.3653	13.3118	13.3322	24.2158
GH28	Gel0090086	1.8207	2.7278	5.5373	5.7327	2.4279
GH28	Gel0066055	0.1332	0.0706	0.1882	0.0688	0.1463
GH28	Gel0099111	4.8647	1.6218	0.2493	0.1672	0.4300
GH28	Gel0047044	13.1865	9.6657	0.6579	0.7948	74.4807
GH28	Gel0130136	5.7364	5.5524	3.7331	4.0441	9.5068
GH28	Gel0073193	0.0000	0.0175	0.0000	0.0000	0.0000
GH28	Gel0073201	6.1906	7.4012	3.2758	2.7575	3.5375
GH28	Gel0015035	29.4416	27.9433	27.8049	25.8458	52.6486
GH28	Gel0001084	82.7928	71.6836	70.6661	26.0490	35.3590
GH29	Gel0048195	12.3400	11.0380	11.8547	10.9431	6.3160
GH29	Gel0148004	24.0718	33.5637	24.3052	10.4857	17.3467
GH3	Gel0019127	11.2478	11.7229	0.4171	0.3795	7.4941
GH3	Gel00101562	21.6513	15.7161	0.7478	0.7480	35.7694
GH3	Gel0050078	67.4715	42.5826	3.3801	5.1989	178.2416
GH3	Gel0114040	85.1378	96.2977	72.2515	36.0481	12.9436
GH3	Gel0041072	0.2114	0.0000	0.0000	0.1286	0.0697
GH3	Gel0227012	120.7955	160.9200	79.4870	77.3286	84.2663
GH3	Gel0023166	57.4266	62.5931	48.5206	42.0194	109.9596
GH3	Gel0050077	0.9606	1.7569	0.4439	0.6333	0.8584

GH3	Gel0093026	87.4550	71.5278	66.8945	52.2495	39.0396
GH3	Gel0079111	1.7807	1.2742	3.4593	2.9823	2.6375
GH3	Gel0050075	24.5338	14.1134	6.6135	6.8169	14.7548
GH31	Gel0076061	16.2615	17.2462	11.4400	10.5696	13.3991
GH31	Gel0188051	15.2216	29.0512	3.6610	1.6272	3.1016
GH31	Gel0152035	74.6393	72.5778	42.5931	23.4473	30.9616
GH31	Gel0105077	0.0000	6.5821	0.1057	0.6189	13.6460
GH31	Gel00121801	28.4466	27.9626	25.9321	25.1099	24.1187
GH31	Gel0152041	0.8023	1.0099	1.1304	1.0310	0.6695
GH31	Gel0279003	71.8104	70.6832	72.1660	61.5656	42.7265
GH32	Gel0111121	10.0259	72.9505	36.8920	2.3451	81.6038
GH32	Gel0054140	0.3341	0.1675	0.0593	0.0470	0.0551
GH32	Gel0294004	40.2119	39.5111	37.8920	39.1051	35.3542
GH32	Gel0054139	57.1650	44.3179	8.2364	5.3598	4.5994
GH33	Gel0115114	71.8419	71.2733	52.3354	47.6110	70.1041
GH35	Gel0090109	36.6522	82.9502	5.7327	2.9414	136.5736
GH35	Gel0247006	26.2792	26.9903	20.0669	24.4382	30.9749
GH35	Gel0042131	37.9886	33.8576	11.1270	5.4291	60.4788
GH35	Gel0247003	128.5734	70.6157	44.1245	56.3382	48.2506
GH35	Gel0006098	8.0097	10.8948	8.8256	8.4921	6.7825
GH35	Gel0145014	88.7279	88.0399	101.3181	70.1658	94.9868
GH35	Gel0016207	0.6195	0.5510	1.7470	1.8578	0.4549
GH35	Gel0006091	5.1894	5.7932	4.3118	4.6126	6.2855
GH35	Gel0006095	8.6559	11.3512	5.5707	4.3151	14.5684
GH35	Gel0051063	16.1663	15.9694	3.4225	1.4058	99.7925
GH35	Gel0081039	2.4077	2.1852	1.7270	1.8915	2.2514
GH36	Gel00301031	26.9605	27.4124	28.0643	28.1105	27.7767
GH37	Gel0069009	64.9418	42.3841	0.1598	0.0354	1.6486
GH37	Gel0007244	30.8668	14.3221	3.7590	4.0167	12.1746
GH37	Gel0114117	3.6915	0.8591	0.4116	0.4015	0.1395
GH37	Gel0022033	11.9584	8.5341	0.0000	0.0000	0.2765
GH37	Gel0069005	3.0202	5.0108	0.0285	0.0000	0.1024
GH37	Gel0163004	25.5084	28.5695	35.0858	28.2643	17.6497
GH37	Gel0069006	8.1820	16.4729	0.2164	0.1964	0.0759
GH38	Gel0054041	292.9617	246.8285	161.7015	96.4313	95.3319
GH38	Gel0028053	28.6439	33.1603	47.7737	44.8382	45.6627
GH38	Gel0196023	45.5038	45.4210	39.0700	26.0021	21.8651
GH43	Gel01060581	62.0655	56.1157	60.4090	56.6998	89.1092
GH47	Gel0113034	1.0203	3.3024	1.4486	1.6697	1.4232
GH47	Gel0220030	12.8117	12.3054	8.3696	7.6650	8.0542
GH47	Gel0035077	102.6848	99.7588	119.4068	97.0183	91.7615
GH47	Gel0178096	25.3503	26.7132	25.7137	22.6189	27.9150
GH5	Gel0076068	0.9026	1.0870	3.3277	1.2828	1.3635
GH5	Gel0076052	10.5626	14.3470	8.4011	4.2194	8.1486

GH5	Gel0290019	11.4963	11.7487	4.7545	1.0295	4.4442
GH5	Gel0076070	69.0299	114.9698	14.5394	6.0846	9.9444
GH5	Gel0094027	0.1211	0.7023	0.0000	0.0000	0.8633
GH5	Gel0066015	16.6962	16.7856	24.3611	22.1312	17.5648
GH5	Gel0011044	37.2615	35.4235	30.7978	22.6853	75.5209
GH5	Gel0164020	0.1313	0.3969	0.1310	0.0592	24.4571
GH5	Gel0140006	50.0435	39.2245	10.6814	8.8330	12.1575
GH51	Gel0124041	22.3264	21.1707	3.2484	2.5927	259.8662
GH63	Gel0021167	36.4199	37.2008	31.6703	33.2283	21.9512
GH72	Gel0010118	106.1487	131.5052	77.0278	69.5659	61.9023
GH77	Gel0122065	56.1413	63.4583	51.1469	64.1420	35.4935
GH77	Gel0037054	93.4400	96.6617	86.3580	82.1349	76.3310
GH79	Gel0220010	21.8357	38.5708	4.8381	4.0990	10.8711
GH79	Gel0014065	34.9977	22.9293	35.4685	25.5536	14.8853
GH79	Gel0033136	28.5257	16.3717	0.1556	0.0531	0.6163
GH79	Gel0142044	75.0677	84.6040	27.0093	25.7272	55.6086
GH79	Gel0037086	32.9073	36.4321	41.1433	52.4123	56.8755
GH81	Gel0124045	55.4665	43.2306	54.2547	93.1745	51.0221
GH9	Gel0047018	62.2519	59.1267	85.7738	70.8131	39.3390
GH9	Gel0250027	0.0000	0.0000	0.0000	0.0000	0.0000
GH9	Gel0089074	52.3943	48.5445	63.0725	45.7223	2.8595
GH9	Gel0044207	2.8531	37.8246	0.2019	0.0344	0.0321
GH9	Gel0000311	181.9657	198.3873	265.7626	213.4733	453.5054
GH9	Gel0020168	1.9781	1.6995	1.9951	1.6129	2.5893
GH9	Gel0006035	2.0676	1.5841	0.5791	0.4031	5.2443
GH9	Gel0080039	0.0375	0.0117	0.0761	0.1079	0.0000
GH9	Gel0004276	0.3380	0.3487	0.1118	0.1320	0.6563
GH9	Gel0099128	21.1234	24.5158	9.3112	8.2235	66.4555
GH9	Gel0085058	0.5173	0.8783	0.1807	0.2269	0.2709
GH9	Gel0096056	15.5181	26.1130	18.1485	16.0037	16.5020
GH9	Gel0064018	0.2055	0.2409	0.0232	0.0236	3.6291
GH95	Gel0130036	6.2609	7.3482	4.6382	4.0490	5.5610

A: protocorms, B: juvenile tubers, C: immature tubers, D: mature tubers, and E: scapes.

Supplementary Table 45 | Transcriptional levels of nitrate transporters (NRTs) and ammonium transporters (AMTs) genes at development stages.

Family	Gene ID	A	B	C	D	E
NRT	Gel0011145	3.5338	3.5776	0.7389	1.2520	4.0338
	Gel0001167	31.7333	42.0323	11.1362	24.2000	163.4463
	Gel0206040	28.1701	64.0199	13.4476	13.7926	45.8801
	Gel0023221	5.5003	17.1671	1.2713	2.3466	0.3954
	Gel0048006	4.4534	6.2663	3.8255	4.4087	5.9321
	Gel0094070	27.8408	24.9952	4.8903	5.8684	7.4682
	Gel0201050	5.0129	7.8276	6.2363	7.3636	9.6832
	Gel0074025	20.4211	18.1004	28.5750	26.8517	17.2437
	Gel0023223	0.0000	0.5482	0.0164	0.0422	0.0455
	Gel0090042	118.2001	135.5406	11.1049	2.8039	8.0658
	Gel0120023	2.4771	4.7285	9.5111	4.8207	0.9294
	Gel0023225	0.1888	0.2855	0.5876	0.3280	0.0415
	Gel0094068	14.6932	3.5090	2.5433	3.5555	1.1782
	Gel0078094	0.0907	0.0396	0.0527	0.0455	1.5113
	Gel0105026	53.5711	38.8975	22.9826	22.5304	80.5285
	Gel0004232	7.0584	9.3766	9.6075	13.0800	23.5525
	Gel0048047	2.2056	4.1269	5.3093	7.5809	14.2229
	Gel0052074	23.4580	21.0579	16.9835	11.9214	57.2553
	Gel0008167	0.3386	6.4474	2.8770	6.2899	1.5119
	Gel0111041	88.9721	47.8857	20.3436	15.9408	5.2185
	Gel0000288	13.2120	21.1284	18.7608	7.6153	1.8307
	Gel0002208	133.4846	99.8421	13.6485	3.3214	5.8340
	Gel0074022	20.2511	13.7470	8.9514	9.8859	12.8728
	Gel0023224	2.3211	1.5593	0.7364	1.7364	0.1101
	Gel0237001	6.2641	7.3922	6.6365	6.5849	4.8091
AMT	Gel0080106	0.0000	0.0801	0.0000	0.1048	0.0000
	Gel0059169	70.0685	64.8693	50.2288	50.3112	51.6942

A: protocorms, B: juvenile tubers, C: immature tubers, D: mature tubers, and E: scapes.

Supplementary Table 46 | Transcriptional levels of Urea-related genes at different development stages.

Family	Gene ID	A	B	C	D	E
Urease	Gel0011205	0.0000	0.0895	0.0647	0.0856	0.0838
	Gel0011215	0.0000	0.0656	0.1976	0.2163	1.3862
	Gel0011216	0.0000	0.0656	0.2002	0.0000	0.2575
	Gel0293003	0.0917	0.1828	0.3188	0.2505	0.9958
	Gel0293007	6.4978	4.6739	5.4896	5.3821	3.2748
	Gel0427003	7.9588	7.2742	8.8398	10.0832	5.1917
	Gel0427004	0.0000	0.2579	0.1128	0.1035	0.4936
	Gel0293005	29.4382	24.8387	26.5497	28.5460	41.3401
	Gel3262001	7.1088	5.9856	4.9565	6.0259	16.3413
arginase	Gel0002294	39.1505	38.8839	34.2281	35.0728	32.3263
URED	Gel0010136	9.4845	7.6402	7.2889	5.2970	10.8044
UREF	Gel0112044	17.7556	26.6339	23.2449	23.4480	18.2200
UREG	Gel0006132	113.0606	117.6603	165.5514	179.5861	95.1218

A: protocorms, B: juvenile tubers, C: immature tubers, D: mature tubers, and E: scapes.

Supplementary Table 47 | Transcriptional level of amino acid transporters at different development stages.

Family	Gene ID	A†	B†	C†	D†	E†
AAP	Gel0074020	8.0702	7.2189	0.8625	1.6297	1.1067
	Gel0186042	18.4771	5.7553	0.6927	1.3662	1.9625
	Gel0074021	40.3727	57.7983	0.1822	0.0436	0.0675
	Gel0124034	119.1694	118.7167	60.0508	65.8774	191.9945
	Gel0077024	15.8186	12.8024	20.4002	36.4662	153.8311
	Gel0048040	114.5902	130.4040	70.2177	101.4785	192.8017
	Gel0001395	89.6843	85.4207	50.9729	185.0564	439.5086
	Gel0015100	64.8052	41.4357	28.8355	36.0554	36.9270
	Gel0038117	14.9095	16.5269	15.1232	15.0612	17.1177
	Gel0079228	48.7226	31.1362	14.3521	14.5829	171.7239
	Gel0162023	1.1408	1.4440	1.5566	2.0097	2.4332
	Gel0139016	15.6120	32.3762	36.1187	41.7290	280.8347
AAT	Gel0079155	22.0393	36.0755	29.2323	34.8632	107.9237
	Gel0019067	0.0000	0.1106	0.0105	0.0465	0.0320
ANT	Gel0255007	6.7946	7.9779	8.4411	9.9882	12.4581
	Gel0050098	47.1081	52.6743	18.8508	18.2746	38.5969
	Gel0193048	6.2785	4.9871	1.9957	1.6988	1.4759
	Gel0023114	24.8395	15.4097	3.4252	3.9345	7.1076
	Gel0065112	34.1289	35.3151	31.6202	30.6674	49.7018
	Gel0124032	24.8135	24.1851	19.4336	19.2034	44.1685
	Gel0059241	0.3017	4.7533	0.0655	0.0601	0.4167
	Gel0123065	13.9700	25.5271	24.4572	26.5315	32.6950
	Gel0003174	77.8121	59.4292	108.1597	130.0629	121.8287
	Gel0154029	16.3975	17.0470	11.9779	13.3857	28.8223
APP	Gel0023266	19.4865	17.5987	12.8261	11.5636	13.4121
	Gel2260001	46.6095	36.8760	10.2262	10.8719	154.3279
	Gel0000221	6.9062	6.5363	8.8189	8.6428	7.9460
auxin	Gel0064158	24.8230	25.8155	31.0967	32.9100	26.3747
CAT	Gel0087093	15.4217	17.4367	14.0674	12.1847	24.2075
	Gel0042213	7.8395	11.0443	10.0607	8.7224	11.1955
	Gel0079093	67.6262	67.5024	37.3568	35.2620	150.6750
	Gel0178092	4.9947	7.6072	1.3835	0.1986	0.0840
	Gel0148067	11.8655	13.2676	14.7370	19.1988	12.0140
GATs	Gel0080196	9.4708	15.4720	9.3200	6.6195	18.8925
	Gel0135017	4.5915	4.4459	2.1382	9.4316	99.9297
	Gel0126003	29.7194	39.4205	55.5112	124.4308	1012.6563
	Gel0017171	7.9460	3.2833	1.0034	0.5573	1.9817
	Gel0092108	3.0420	3.8657	0.8850	0.8169	0.9582
LHT	Gel0090041	22.7788	33.1431	71.9406	47.6840	15.3614

	Gel0187008	3.8243	3.4785	1.2541	1.9296	3.7940
	Gel0016028	24.7458	14.4889	5.6633	2.9896	34.1753
	Gel0198054	137.3954	113.7988	41.3548	30.9962	22.4914
PROT	Gel0148002	1.7623	1.0573	1.0081	1.1372	1.4375
	Gel0001188	38.5464	52.0433	14.1289	3.1225	14.9633
	Gel0008177	9.0531	10.6825	14.4357	19.8017	10.7463
UPS	Gel0036081	29.4235	47.9459	38.9089	22.5617	32.7134

* amino acid permeases (AAPs), lysine-histidine-like transporters (LHTs), proline transporters (ProTs), γ-aminobutyric acid transporters (GATs), ANT1-like aromatic and neutral amino acid transporters (ANT) and auxin transporters, Cationic amino acid transporters (CATs). † A: protocorms, B: juvenile tubers, C: immature tubers, D: mature tubers, and E: scapes.

Supplementary Table 48 | Transcriptional levels of phosphate transporter at different development stages.

**Family**	**Gene ID**	**A**	**B**	**C**	**D**	**E**
Phosphate transporter	Gel0000225	40.3664	45.3742	48.3655	42.6109	56.2413
	Gel0131013	7.6051	9.4270	5.0943	7.6878	16.3683
	Gel01030181	13.7941	15.5051	17.7919	18.5585	12.2658
	Gel0251005	21.3122	22.2698	9.1734	7.6120	27.9903
Acid phosphatase	Gel0050179	5.3167	3.4315	0.1873	0.4309	3.2206
	Gel0095013	33.8066	37.9592	26.0387	48.8087	131.6640
	Gel0112041	37.8030	149.4124	37.1674	17.1507	20.6124
	Gel0186063	26.9040	37.7077	16.3066	12.1912	41.2866
	Gel0173004	2.9403	38.1989	0.1181	0.4069	0.6165
	Gel0187037	1.8364	4.3094	2.7055	2.2802	0.9580
	Gel0050180	8.2910	8.5965	0.5118	1.3221	4.7897
	Gel0000108	61.5290	53.8651	53.2143	56.5478	126.7225
	Gel0112040	0.0943	0.0702	0.0000	0.0141	0.0166
H^+ ATPase	Gel0101049	122.7703	120.5610	14.0046	7.6655	24.6546
	Gel0114014	12.4051	10.3929	7.3629	9.2959	12.7366
	Gel0120031	144.5733	167.8260	129.6922	148.4985	148.7399
	Gel0130056	78.4971	84.0367	35.1238	21.7424	72.4547
	Gel0147018	126.3852	136.7747	33.5424	35.3818	207.1503
	Gel0014098	15.1656	13.8118	12.8515	11.8867	27.8186
	Gel0032061	15.6526	18.1770	16.8639	14.4020	18.7682
	Gel0337001	74.1034	72.5563	35.8564	32.3026	76.3414
	Gel0037058	67.4118	68.8260	44.7221	37.6861	29.1660
	Gel0063060	4.5805	11.4922	3.8579	0.8845	3.8893
	Gel0087098	34.1360	44.8456	0.3411	0.3711	1.2825
	Gel00880152	286.0155	206.3044	58.7925	95.6845	735.3274
	Gel0088031	4.8981	5.1083	5.3130	4.4361	2.7353
H^+/Pi symporter	Gel0104055	0.0351	0.1711	0.0352	0.1571	0.2201
	Gel0104057	12.0662	36.6359	24.7690	32.5536	22.8383
	Gel0107033	14.6394	15.2192	8.1141	5.3045	11.6636
	Gel0035019	6.6988	17.3920	4.0296	2.3485	7.9064
	Gel0053055	11.5610	12.5770	13.9548	13.0424	16.2197

A: protocorms, B: juvenile tubers, C: immature tubers, D: mature tubers, and E: scapes.

Supplementary Table 49 | Transcriptional levels of K^+ transporter at different development stages.

Family	Gene ID	A	B	C	D	E
HAK	Gel0019164	52.4577	30.6630	18.2916	23.5955	24.8764
	Gel0020158	7.3562	5.2675	2.7567	1.4846	1.8843
	Gel0028019	45.8543	71.1080	46.4734	42.1152	22.0536
	Gel0050217	9.1380	13.7378	7.7056	12.5269	22.7507
	Gel0158029	22.4795	23.3319	23.2732	20.8613	22.8802
	Gel0016195	16.6448	16.1192	15.2206	16.2003	7.7992
	Gel0078157	2.5491	3.3398	2.2242	2.6142	5.5538
	Gel0126004	42.7611	44.9680	96.6773	95.1651	93.3516
	Gel0064148	28.0594	23.8716	40.7817	43.8993	57.1988
	Gel0139068	5.2521	10.3142	2.8052	8.7293	171.1987
	Gel0115102	40.8646	37.1380	36.5062	33.4866	31.8871
shaker_like_K	Gel0138004	0.9500	1.6359	1.0439	1.1896	1.4879
	Gel0180007	6.4170	8.3907	9.2280	7.1065	1.9505
	Gel0008127	8.3601	12.0058	7.1199	8.8887	11.9447
	Gel0099067	17.9906	33.6917	11.2419	13.4848	26.7892
	Gel0113038	1.4724	2.4994	2.0880	1.9460	0.8120
	Gel0107001	1.1705	4.3657	0.1246	0.1281	0.1168
TPK	Gel0178036	13.0925	12.9915	15.3097	21.2848	6.8204
	Gel0011170	12.3124	12.9631	11.1101	10.5461	17.4373

high affinity K^+ transporter (HAK), shaker K^+ channels (shaker_like_K), two-pore K^+ channel (TPK); A: protocorms, B: juvenile tubers, C: immature tubers, D: mature tubers, and E: scapes.

Supplementary Table 50 | Transcriptional level of C transporters genes at different development stages.

Family	Gene ID	A	B	C	D	E
SWEET	Gel0032021	0.000	0.000	0.000	0.000	0.130
	Gel0044071	0.289	0.130	22.867	69.704	10.814
	Gel0022066	0.204	0.089	0.118	0.000	1.041
	Gel0095118	172.752	149.006	98.549	125.439	594.554
	Gel0115006	3.564	6.295	14.520	16.898	9.814
	Gel0118021	0.000	0.049	0.079	0.095	0.026
	Gel0073165	38.108	34.234	24.359	26.069	18.119
	Gel0066041	37.937	26.022	0.185	0.048	0.000
	Gel0062102	358.082	274.423	1.776	2.691	5.759
	Gel0001291	135.827	101.122	51.739	21.979	5.757
	Gel0066042	0.447	1.489	0.052	0.000	0.000
TPT	Gel0066023	18.207	15.491	5.857	4.695	3.292
pGlcT	Gel0063002	38.628	51.928	51.539	43.978	34.862
MEX1	Gel0097021	26.858	26.522	23.276	19.822	9.869
TMT	Gel0059184	10.893	9.133	10.727	11.050	9.189
	Gel0235014	64.454	65.031	73.976	58.129	66.777
SUC4/SUT4	Gel02570311	56.880	49.114	47.549	43.690	56.112
ESL1	Gel0040015	73.925	61.958	76.604	76.979	84.178
SUC3	Gel0004085	33.453	39.880	44.805	29.137	49.159
	Gel0187079	182.557	151.007	17.668	17.756	329.276

A: protocorms, B: juvenile tubers, C: immature tubers, D: mature tubers, and E: scapes.

Supplementary Table 51 | Transcriptional levels of strigolactone signal related genes at different development stages.

Family	Gene ID	A	B	C	D	E
CCD7	Gel0108025	1.651	0.946	0.291	0.353	1.205
	Gel2108001	0.137	0.115	0.097	0.083	0.019
	Gel0108026	1.469	4.078	0.824	0.675	7.433
	Gel0201011	4.614	8.976	7.245	4.312	1.086
	Gel0108028	0.659	0.187	0.250	0.220	0.157
CCD8	Gel0039029	17.285	24.819	27.272	28.624	49.572
PDR1	Gel00170781	8.065	5.138	2.466	2.687	4.751
PDR1	Gel0107027	46.733	69.026	3.659	1.491	1.955
PDR1	Gel0113029	22.399	19.905	5.645	3.126	2.067
PDR4	Gel0119024	17.390	15.245	16.090	14.102	17.797
PDR8	Gel0047040	0.913	0.853	0.463	0.624	1.939
DR8	Gel0186037	75.237	83.883	58.192	66.360	68.841
PDR9	Gel0071108	26.839	62.422	138.261	161.908	142.519
PDR9	Gel0011021	31.878	26.900	7.382	8.223	10.228
PDR9	Gel0129093	24.610	29.074	1.548	0.075	0.247
PDR9	Gel00110241	2.836	2.289	0.010	0.005	0.000
PDR9	Gel0023261	0.246	0.190	0.026	0.004	0.652
PDR9	Gel0033085	7.422	7.019	4.491	4.202	6.036
PDR9	Gel0011024	4.233	3.030	0.368	0.275	0.124
PDR9	Gel0071107	0.547	0.744	1.194	1.083	3.695
PDR9	Gel0023263	78.441	71.612	69.348	65.854	27.925
PDR9	Gel2072001	20.158	42.796	101.161	134.174	148.500
PDR9	Gel0099062	7.303	6.407	5.819	2.681	1.923
PDR9	Gel0099058	22.974	29.441	23.601	39.549	18.152
PDR10	Gel0155004	1.449	1.985	0.359	0.322	0.269
PDR10	Gel0013096	81.625	74.605	47.398	38.452	16.468
PDR10	Gel0017165	21.245	18.902	7.479	5.411	11.583

A: protocorms, B: juvenile tubers, C: immature tubers, D: mature tubers, and E: scapes.

Supplementary Table 52 | Transcriptional levels of chitooligosaccharides and lipochitooligosaccharides signal related genes at different development stages.

Family	Gene ID	A	B	C	D	E
LysM	Gel0073007	17.484	17.087	18.913	19.639	85.032
	Gel0054150	34.383	25.593	0.737	0.817	3.162
	Gel0163038	63.476	116.087	15.163	14.081	64.043
	Gel0044116	10.523	10.301	13.308	13.202	11.524
	Gel0008269	22.174	31.062	17.167	18.938	25.775
	Gel0005300	66.679	93.312	85.167	137.849	28.885
	Gel0038123	4.104	8.227	1.099	0.964	0.346
	Gel0026173	24.665	22.473	15.361	26.799	47.019
	Gel00541561	14.924	15.238	12.141	11.538	33.734
	Gel0104016	21.102	30.919	15.200	11.693	27.091
	Gel00162441	53.398	45.233	44.851	40.017	50.587
	Gel0151044	3.212	7.449	0.244	0.000	0.051
	Gel0063130	38.025	65.525	36.700	42.853	42.036
	Gel0112140	27.133	49.543	2.709	2.185	2.769
	Gel0047090	23.051	29.244	21.841	18.739	37.827
	Gel0012263	13.182	14.974	18.694	16.593	13.399
	Gel0004159	19.009	19.437	14.781	22.670	22.862
	Gel0080198	0.842	0.975	0.156	0.016	0.041
	Gel0073008	99.591	93.574	112.058	137.781	185.705
	Gel0207032	46.252	47.313	38.476	38.208	40.155
	Gel02570211	0.000	0.122	0.073	0.229	0.075
	Gel00540561	31.134	34.302	25.708	25.760	30.310
	Gel0142048	2.629	2.885	1.981	1.260	1.122
	Gel0112082	9.048	7.657	9.478	9.627	6.553
	Gel0008213	20.918	25.073	24.898	20.008	17.855
	Gel0160015	33.479	39.291	48.792	41.915	55.302
DMI2	Gel0020140	6.277	14.458	25.994	18.790	3.266
	Gel0001286	4.659	4.265	5.122	5.867	20.809
	Gel0048079	29.624	27.966	16.018	13.469	25.065
	Gel0016001	18.583	17.964	4.307	3.261	12.893
	Gel0079222	6.809	12.687	26.150	32.963	32.871
	Gel0063219	44.082	35.541	37.879	32.579	45.013
	Gel0095100	1.562	1.794	0.775	0.310	0.582
	Gel0079224	1.043	1.289	0.592	0.999	12.817
	Gel0001287	23.361	27.302	21.899	21.273	57.268
	Gel0168032	1.818	2.376	2.630	2.269	2.928
	Gel0177049	27.514	30.487	40.482	35.880	113.656
	Gel0068109	2.958	2.985	4.372	8.927	4.190
	Gel0108086	4.193	5.341	4.238	2.867	2.185
	Gel0179054	9.497	11.640	9.067	6.951	14.493

	Gel0112011	7.239	6.104	6.409	4.778	8.865
	Gel0054029	16.116	9.138	4.565	3.117	5.318
	Gel0016103	7.256	8.293	0.583	0.511	0.309
	Gel0043128	25.281	14.496	3.642	1.312	0.823
	Gel0015171	39.882	30.843	19.969	25.950	19.819
	Gel0078081	6.499	6.525	8.594	6.351	2.882
	Gel0026003	3.208	3.076	0.378	0.329	0.485
	Gel0094008	13.974	16.923	9.741	10.231	11.609
CASTOR /POLLUX	Gel0125012	16.550	14.517	13.733	14.421	15.803
DMI3	Gel0042108	49.412	52.331	45.882	36.871	25.047
	Gel0078044	75.809	69.914	109.525	122.470	73.749
	Gel0047065	56.628	54.480	65.742	48.084	32.896
	Gel0124056	6.039	5.452	7.210	7.824	7.437
	Gel0007013	42.007	35.907	34.530	31.448	44.419
	Gel01080671	27.027	25.319	32.392	36.801	82.187
	Gel0130130	26.104	23.591	45.480	39.134	32.473
	Gel0191010	16.059	17.407	16.683	15.673	16.743
	Gel0004391	39.117	37.511	31.913	35.056	35.348
	Gel0120021	9.419	13.236	18.499	11.436	8.084
IPD3	Gel0167034	19.769	18.468	18.445	17.884	19.983
RAM1	Gel0038146	0.613	0.302	0.385	0.453	0.161
	Gel2142001	25.852	26.620	22.459	16.716	8.983
	Gel0002166	37.491	25.313	2.918	4.316	22.461
	Gel0094105	4.663	5.053	2.547	3.128	7.051
	Gel0036130	5.146	3.453	0.862	1.346	4.251
	Gel0002288	0.174	0.325	0.000	0.012	0.000
	Gel0130102	40.598	47.099	37.332	38.303	38.982
	Gel0103058	53.150	103.273	16.130	7.680	25.862
	Gel0249005	41.892	37.912	46.610	63.511	39.323
	Gel0127001	9.825	13.033	0.988	1.062	10.948
	Gel0275016	20.266	16.045	2.860	3.936	28.979
	Gel0086073	25.196	29.149	4.467	4.893	1.192
	Gel0000138	63.605	64.624	58.244	58.682	66.552
	Gel0008209	11.920	18.977	54.947	53.077	8.491
	Gel0013011	93.461	84.192	103.298	133.663	81.946
	Gel0015158	4.527	1.873	0.295	0.023	0.166
	Gel0008212	0.060	0.063	0.318	0.052	0.016
	Gel0158010	40.937	47.441	22.567	15.751	3.445
	Gel0163023	47.818	33.766	72.480	92.297	37.430
	Gel0060141	10.763	14.997	41.256	31.477	5.367
	Gel0078222	79.379	62.222	84.089	95.818	25.753
	Gel0232026	21.278	31.823	25.154	23.425	11.489
	Gel0001261	24.450	10.516	9.817	6.487	3.056

	Gel00941061	108.158	93.129	101.360	92.987	56.282
	Gel0045063	18.296	24.098	5.665	5.210	14.904
RAM2	Gel0135014	46.082	37.932	17.052	6.586	3.972
	Gel0161023	16.204	27.160	9.941	16.146	7.406

A: protocorms, B: juvenile tubers, C: immature tubers, D: mature tubers, and E: scapes.

Supplementary Table 53 | Transcriptional levels of *NBS* genes at different development stages.

**Class**	**geneID**	**A**	**B**	**C**	**D**	**E**
NBS	Gel0004145	3.5223	5.4078	12.3583	13.0650	4.2746
NBS-LRR	Gel0048116	6.1145	5.9882	4.7276	3.8547	3.4072
CC-NBS	CADD01482.1	0.1130	0.2180	0.5199	0.3851	0.3136
CC-NBS-LRR	Gel0090020	3.2556	2.8812	0.7798	1.2764	4.5357
TIR-NBS	Gel0015018	5.8572	4.0498	4.4169	2.6442	1.9594
TIR-NBS	Gel0129058	23.2069	24.2609	24.5872	20.5293	15.8800

A: protocorms, B: juvenile tubers, C: immature tubers, D: mature tubers, and E: scapes.

Supplementary Table 54 | Transcriptional levels of antioxidant genes at different development stages.

Name	Gene ID	A	B	C	D	E
SOD	Gel0080217	434.9988	638.9149	262.6482	178.0917	382.6214
SOD	Gel00041491	139.3582	143.0746	128.9706	113.6134	13.0565
SOD	Gel0027125	33.8496	41.2912	23.8832	20.6839	11.7835
APX	Gel0081042	148.5878	147.1383	143.2400	117.0071	136.3904
APX	Gel0103050	0.1123	0.0000	0.0000	0.0386	1.9825
APX	Gel0139024	93.1394	107.1044	60.4685	70.0890	59.2587
APX	Gel0080046	864.6447	1116.6435	422.0423	363.3322	1568.4533
APX	Gel0008279	0.0000	0.0000	0.0000	0.0000	0.0000
APX	Gel0206044	442.5621	556.0851	211.7958	143.9011	76.1864
GR	Gel0187003	168.4033	193.8858	115.1901	82.1066	104.4355
GR	Gel0070050	20.4167	25.5154	16.8445	19.1020	13.5884
CAT	Gel0095044	0.0000	0.0400	0.0000	0.0000	0.0000
CAT	Gel3598001	0.0000	0.0000	0.0000	0.0000	0.0000
CAT	Gel0003193	79.6158	66.2026	40.8824	53.5280	30.9658
CAT	Gel0104015	1408.8846	1171.3889	1425.5782	965.7244	1354.8419
NADPH Oxidase	Gel0135060	1.2333	1.6703	1.7304	0.8938	18.1821
NADPH Oxidase	Gel0053034	12.5871	11.2956	7.7972	6.9682	15.7104
NADPH Oxidase	Gel0020136	66.0012	122.1085	122.3649	160.4783	86.5247
AOX	Gel0208043	69.2767	74.1840	76.0932	86.7353	116.2847
AOX	Gel0163046	70.2739	109.7474	125.7786	100.0683	170.5772
PrxR	Gel0013151	77.2521	97.6548	24.2500	18.0639	50.8999
PrxR	Gel0006121	1831.2329	1830.9544	1542.4367	1305.8757	1305.4432
MDAR	Gel0205008	10.9265	11.2873	11.0695	12.3837	13.2935
MDAR	Gel0054095	545.0074	548.2355	635.0901	654.9849	607.8418
MDAR	Gel0137054	18.6595	20.8471	15.4369	14.8712	27.9804
MDAR	Gel0080188	120.6353	160.7504	52.6268	54.8997	70.5763
ferritin	Gel0140018	404.1863	808.5623	520.0041	440.2390	115.6523
frataxin	Gel01090871	47.8457	45.7988	39.2009	32.8334	49.0365

A: protocorms, B: juvenile tubers, C: immature tubers, D: mature tubers, and E: scapes.

Supplementary Table 55 | Transcriptional levels of *MCP* at different development stages.

Name	Gene ID	A	B	C	D	E
MCP1	Gel0060105	36.3999	40.5457	23.3669	25.0749	66.1826
MCP1	Gel0072019	4.7386	5.5012	3.2393	2.9996	13.7422
MCP5	Gel0158011	242.0254	177.6841	255.1632	350.0878	164.3929

A: protocorms, B: juvenile tubers, C: immature tubers, D: mature tubers, and E: scapes.

Supplementary Table 56 | Transcriptional levels of *PAL*, *C4H* and *ADH* at different development stages.

Name	Gene ID	A	B	C	D	E
PAL	Gel1859001	32.9054	46.8055	111.3182	181.4503	105.5057
PAL	Gel0201001	0.0257	0.0096	0.0713	0.2321	0.0245
PAL	Gel0097043	776.4687	908.7599	293.2305	326.5307	2429.2862
PAL	Gel0201003	0.0771	0.1094	0.2015	0.5417	0.1827
C4H	Gel0002215	1208.4708	1225.7523	605.6300	872.1393	1718.1611
ADH	Gel0045013	554.2259	551.4558	254.0274	327.3222	428.9230

A: protocorms, B: juvenile tubers, C: immature tubers, D: mature tubers, and E: scapes.

Supplementary Table 57 | Transcriptional levels of jasmonic acid (JA) synthetic genes at different development stages.

Name	Gene ID	A	B	C	D	E
JAR1	Gel0089049	78.4597	82.1488	40.8137	43.0602	70.7065
JAR1	Gel0100078	60.8822	80.8629	63.4937	87.6596	184.9413
LOX	Gel0019083	38.4945	35.2952	64.8760	49.9625	12.7072
LOX	Gel0007213	3.7174	2.8747	2.6262	3.5455	4.1408
LOX	Gel0068015	48.7461	92.5921	107.5305	180.0932	132.9290
AOS	Gel0071075	6.6588	2.3950	0.1306	0.0792	0.1339
AOS	Gel0005085	55.9400	51.5166	60.5683	55.8037	35.3349
AOS	Gel0039023	3.8253	3.9114	8.0597	7.1843	5.1430
AOC	Gel0088148	104.0377	130.3288	77.2099	81.5114	72.6260
12-OPR	Gel0067016	30.3543	137.1927	34.0299	8.1341	215.5033
12-OPR	Gel0054051	212.7382	180.4389	14.7970	3.7644	31.6219
12-OPR	Gel0054054	0.1901	0.0000	0.0000	0.0000	0.0000
12-OPR	Gel0054052	13.9212	1.1154	0.0000	0.0000	0.0999
12-OPR	Gel0054055	0.0000	0.0000	0.0000	0.0000	0.0000
12-OPR	Gel0068030	10.4430	13.3099	20.6816	21.2761	9.6911
12-OPR	Gel0074035	10.4653	19.6944	0.2396	0.3700	0.8677
12-OPR	Gel0156014	353.9792	359.5412	191.4354	125.8970	132.2424
β-oxidation	Gel0001352	117.5216	119.0672	112.8395	105.3428	87.3625
β-oxidation	Gel0228017	62.9633	67.9690	56.7145	57.9911	64.9684
β-oxidation	Gel0072015	35.7691	47.1769	31.7338	66.8362	195.5623
β-oxidation	Gel0037106	11.5473	11.4958	14.5770	13.0455	6.0658
β-oxidation	Gel0227037	0.9126	1.0206	1.0528	0.8813	1.0856
β-oxidation	Gel0063217	105.4722	96.7980	64.8791	53.6808	101.0063

A: protocorms, B: juvenile tubers, C: immature tubers, D: mature tubers, and E: scapes.

Supplementary Table 58 | Transcriptional levels of ethylene (ET) synthetic genes at different development stages.

Name	Gene ID	A	B	C	D	E
ACCO	Gel0208047	133.9656	250.1875	145.0241	259.2257	4065.0558
ACCO	Gel0064052	0.0000	0.0000	0.0000	0.0000	0.0000
SAMS	Gel0004115	9.2078	8.1820	5.5981	2.6000	3.3651
SAMS	Gel0008063	396.9515	348.7040	465.6595	395.2079	243.0219
SAMS	Gel0020095	616.5557	614.3915	708.0450	934.5706	1600.1694
SAMS	Gel0125031	620.9346	953.9658	823.1443	1114.8071	6070.5063
ACCS	Gel01000692	9.0106	6.6889	6.1142	4.4957	0.0976
ACCS	Gel0102006	0.2402	0.0683	0.0130	0.0138	0.0469
ACCS	Gel0002346	2.5350	15.5364	20.1378	53.6180	28.0352

A: protocorms, B: juvenile tubers, C: immature tubers, D: mature tubers, and E: scapes.

Supplementary Table 59 | Details of candidate genes and primers used in qPCR.

Gene name	Forward Primer (5'→3')	Reverse Primer(5'→3')
β-actin	GGGGATGAAGCACAGTCCAA	GCCGTGGTTGTGAAGGAGTA
Gel0220010	GTGGCAGTCCTCAAGTCTCC	GCACGGTCGTGTTGATTGTC
Gel0087098	GTTCCAGAAGCTGGTCCCTC	GAGAAGCTTGGCTCCGAAGT
Gel0076070	GACCGGCTTCGACAAGGTAA	ATTCCCTTCACTGTGGGACG
Gel0113034	ACCACGGCTTTCACCTGATG	TGACCTGGCCCTTTGTGTTA
Gel0023221	TGACGACTCAAACCCTGACG	ATTTGGTCGGTATGGGGCAG
Gel0090109	TGGTGCCGGAATTACTGGTC	AACCATGGACCGAGGCAAAT
Gel0044207	GAGCACCGCTTATGGGAAGT	CCCACAAGAGGGGCATTGAT
Gel0131048	TCTCACCAGTCCCGATCCAT	CCTTGTACCTGGAGACGACG
Gel0126003	TCGAGTATCGAACCCGCAAG	CTTGGCCATGTAGGAAGGCA
Gel0173004	TCGTGATCTCAACGGGTGAC	TCAGTGTCGCCTTGATAGCC
Gel0059241	TAGCTAAAGGCGGGTGGTTG	CATGTCGTCGGGAGGATCAG
Gel0107001	TCGGGTCCACAACTTTTGCT	TAAGCCAGATGCTCTGCTCG
Gel0099111	ACGGACGACACAGAAGCATT	TGCAAGGGAGATTCCACCAC
Gel0076061	TCAGCACGAACGCAACCTAT	ATTCGAGGAGCCCGAAAAGG
Gel0105077	CATTTCGCGTCCGATGGTTC	ACATCGACATTCCACAGCGT
Gel0023223	CATTCGCTCAACTCAGCAGC	AGACTCTTCGTCCCATCCGA
Gel0099067	CGGACCTCTCTTATGGCGAC	CAACTCAGAAGGGCAGTCGT
Gel0112041	CAACTGCGAACCGCCATAAG	ACCATCGCTGCTCTTCATGT
Gel0178092	GCCTACGGAGGATTCGACAG	AATGTGAGTCGTGTAGCGGG
Gel0044071	TGGCGTTATTCAGCGCAATG	CCCAGAACTTTGAGGCGAGT
Gel0115006	ATCGATTCTGGTGTTCGCGT	TCCAACAGTTAGTCCCGCAC
Gel0062102	CCTTCTCCGTCAGCGTCTTT	CATGCTCCGGCAACTTATGC
Gel0066042	GCCATCGAGCTCTGCTACAT	CCTTGGCCTTTCTCTCAGCA
Gel0108026	CCAGCCGATCTTATCCGGTC	CATTGGAGCAAAGCCACTCG
Gel00170781	TGCGATCTTGGCCAACTCAT	ACAGCAATGGTGTAGCCCTC
Gel0107027	CCGAAATGGTGGGTGTGGTA	GCTGGAAATTAAGAGCGCGG
Gel0113029	TGAACCGACATCCGGTCTTG	TGCTGGGTTGCATCCATCAT
Gel0011021	GCCTGGAACAAATCAGACGC	TGGAAGCACCATTCCCTTCC
Gel0129093	TTCATCGCGAGAACCCTCAG	TCGATGCGGTCTTTGAGCTT
Gel00110241	AGAGCTTGTTGCCAACCCTT	TGTGCTACGTTGGTCACCTC
Gel0011024	AGCTTGTTGCCAACCCTTCT	GTGCTACGTTGGTCACCTCA
Gel0155004	AGGCTCACAATTGCAGTGGA	TACTCCTGGGATGGCCTCAA
Gel0017165	CTCAATGGGGTCAGCGGAAT	CATATGTCTCTCCCACGCCC

Supplementary Table 60 | Details of primers of mitochondrial genes used in qPCR.

Gene name	Forward Primer(5’→3’)	Reverse Primer(5’→3’)
β-actin	GGGGATGAAGCACAGTCCAA	GCCGTGGTTGTGAAGGAGTA
atp1	TTGAATTTGCCAGCGGTGTG	TTCGACACGTCTTCGTTCGT
atp4	ACTCTCGACGGGAGAATCCA	CGGCATAACAAAGCTTGCACT
atp6	TTTCCGGGTCTGAATCCGTC	GCTAGATCCAGGGCCTCTTT
atp8	GAACCAACTGCTTTCGCACC	TCCGACTGGAAAAAGGACGC
atp9	AAGGTGCTAAATCAATGGGTGC	CAGAAACGCCATCATTGGGG
ccmB	CCCTGGAACACCAATCACGA	GAAGAACCCGGTGACCTACC
ccmC	CAAGTCTTCGGGTAGCACCA	CACATAGGCCTTCCCCGAAA
ccmFc	TTACTTCCATGGTCGTGCCT	TCAGAACGGGAACGAAACGA
ccmFn	TCCGGGTCTTTTCGTTGCAT	TGGCGGTAACAGAATCCAGAA
cob	CGGTCCGTTAGCTGGTCTTT	ACTCCGAGACACCGAACAAA
cox1	CCCGGCGATCAAATTCTTGG	GACTTGGTGGCAACAACCAG
cox2	CAATCGCTCCTTGTGATGCG	CCATGAACAATCCTTTGCGGG
cox3	TTTGGCAACCACCGTAGGAG	CCCGAGGATCTAAAACCCCA
matR	CGGCCCTAAGACGGATCAAA	GTACACTGTGTGGGACGGAG
mttB	TTCGACTGGCGTCTCACATC	GAGGCCTCCGTTGATTGTGT
nad1	TTGTGCGCCTTGTGACTGTA	GCATCCCGCGCATATTCAAC
nad2	GGGTTGGCTTGGATTCTGGA	TGGCAATTGAATCATGAGCCG
nad3	TTTGATCCCGCTCGGTCTTC	CGATCCGAAGCACCCCTTTT
nad4	AAGTGGTCTTCTTCCGTGTCC	CATACCAGACCAACCCACTGA
nad4L	AGGTATTCGGGGAATCCTTCTT	AGTCCCTCGGACTCGAAAAG
nad5	ACCACCACGTGCGTATCATT	TCCAGTCACCAACATTGGCA
nad6	TTCCCATCCCAGTCTTTCGC	CCTTTCCGGCATGAACCGTA
nad7	AACGGAGAAGTGGTGGAACG	TCTACGGCTGAAGAATGAGCG
nad9	GGAGTTGATCATCCCTCCCG	CGGCGTAAATCCGGATGATTG
rpl16	TTCAGGTGTCAAAGTGCGGA	ACCCGTACTAGCGCCTTTTC
rpl2	GCGCAGCGGTTTCACTTTTA	AAAGATGGTGGCCGTGGTAG
rpl5	CCTCCAAGCAGGAAAGTCGT	TCCGGGGAGAATTCGCAAAA
rps1	CGAGCCACCAGGTTTGAGAA	CCTGCGATGGCTACCGAAT
rps10	GCGTGACACACGGAAGATTG	AGAAGGCGATAGTCGAAGCA
rps11	ACGCCAGTCGAATCTCGAAG	TCGAAAACCTTCTCTCCAGCTC
rps12	AAGAAAAACGGCGCACAGAC	AGCAAATCCTTGACTCCCCG
rps13	TCAGTTCCCGATGAACAAGTCA	GATGTCTGCTCGTTCTCCCC
rps14	CAAACGTAGATTGCTCGCGG	ATGCCCATCAAAGGACCTCG
rps19	TGGAAGGGCAGTTTTGTTGA	GAAGGTCTTCGTCTCCGTGTA
rps2	CCCATTCGTCAAAAAGGCCG	CACAATCAGGTTGTTGCCCG
rps3	GCTTTCGTCTCGGTAGGTGT	CCCGGCTGATCAACTCAACT
rps4	ACGTGTCGTCTACTTCCAGG	CGATGACTTATCGGCTGCCT

Supplementary Methods

Phylogenetic analysis For the phylogenetic analysis of the analyzed gene families (**Supplementary Table 27, 28, 38, 39, 40, 41**), full-length amino acid sequences for *Gastrodia elata*, *Arabidopsis thaliana*, *Phalaenopsis equestris*[17], *Dendrobium officinale*[79] and *Ananas comosus*[11] were retrieved from their genome database (*A. thaliana*: www.arabidopsis.org/; *ref*. 1-3). All the analyses were conducted by MEGA5[80]. Multiple sequence alignments were created with CLUSTALW for the simple modular architecture research tool (SMART)-defined homeodomain sequences and manual identification[81.] The positions containing gaps were eliminated. The phylogenetic reconstructions for different protein family members were inferred by the Neighbour-Joining method on the p-distance model with 1000 bootstrap replications[82] (**Supplementary Fig. 16-35**).

Supplementary References

1. Schlicker, A., et al. *A new measure for functional similarity of gene products based on Gene Ontology*. BMC Bioinformatics, 2006. **7**: 302.
2. Wicker, T., et al. *A unified classification system for eukaryotic transposable elements*. Nature Review Genetics, 2007, **8**(4):276.
3. Goff, S.A., et al., *A Draft Sequence of the Rice Genome (Oryza sativa L. ssp. japonica).* Science, 2002. **296**(5565): 79-92.
4. Harholt, J. and P. Ulvskov, *Genome sequencing and analysis of the model grass.* Nature, 2015. **463**(7282): 763-8.
5. Bennetzen, J.L., et al., *Reference genome sequence of the model plant Setaria.* Nature Biotechnology, 2012. **30**(6): 555-561.
6. Schnable, P.S., et al., *The B73 maize genome: complexity, diversity, and dynamics.* Science, 2009. **326**(5956): 1112.
7. Mayer, K.F.X., et al., *A physical, genetic and functional sequence assembly of the barley genome.* Nature, 2012. **491**(7426): 711-6.
8. Brenchley, R., et al., *Analysis of the bread wheat genome using whole-genome shotgun sequencing.* Nature, 2012. **491**(7426): 705-710.
9. Jia, J., et al., *Aegilops tauschii draft genome sequence reveals a gene repertoire for wheat adaptation.* Nature, 2013. **496**(7443): 91-5.
10. 70 Paterson, A.H., et al., *The Sorghum bicolor genome and the diversification of grasses.* Nature, 2009. **457**(7229): 551-556.
11. Ming, R., *et al*. The pineapple genome and the evolution of CAM photosynthesis. *Nature Genetics,* 2015. **47**: 1435.
12. Li, L., Stoeckert, C. J., Jr. & Roos, D. S. OrthoMCL: identification of ortholog groups for eukaryotic genomes. *Genome research,* 2003. **13**: 2178-2189.
13. Almssallem, I.S., et al., *Genome sequence of the date palm Phoenix dactylifera L.* Nature Communications, 2013. **4**(9): 2274.
14. D'Hont, A., et al., *The banana (Musa acuminata) genome and the evolution of monocotyledonous plants.* Nature, 2012. **488**(7410): 213-217.
15. Olsen, J.L., et al., *The genome of the seagrass Zostera marina reveals angiosperm adaptation to the sea.* Nature, 2016. **530**(7590): 331.
16. Wang, W., et al., *The Spirodela polyrhiza genome reveals insights into its neotenous reduction fast growth and aquatic lifestyle.* Nature Communications, 2014. **5**: 3311.
17. Cai, J. *et al.* The genome sequence of the orchid *Phalaenopsis equestris*. Nature genetics, 2014. **47**: 65.
18. Jaillon, O., et al., *The grapevine genome sequence suggests ancestral hexaploidization in major angiosperm phyla.* Nature, 2007. **449**(7161): 463.
19. Myburg, A.A., et al., *The genome of Eucalyptus grandis.* Nature, 2014. **510**(7505): 356-362.
20. Ming, R., et al., *The draft genome of the transgenic tropical fruit tree papaya (Carica papaya Linnaeus).* Nature, 2008. **452**(7190): 991.
21. Initiative, A.G., *Analysis of the genome sequence of the flowering plant Arabidopsis thaliana.* Nature, 2000. **408**(6814): 796.

22. Hu, T.T., et al., *The Arabidopsis lyrata genome sequence and the basis of rapid genome size change.* Nature Genetics, 2011. **43**(5): 476-81.
23. Wu, H.J. and Q. Xie, *Insights into salt tolerance from the genome of Thellungiella salsuginea.* Proceedings of the National Academy of Sciences of the United States of America, 2012. **109**(30): 12219-12224.
24. Wang, X., *The genome of the mesopolyploid crop species Brassica rapa.* Nature Genetics, 2011. **43**(10): 1035-1039.
25. Liu, S., et al., *The Brassica oleracea genome reveals the asymmetrical evolution of polyploid genomes.* Nature Communications, 2011. **5**(5): 3930.
26. Chalhoub, B., et al., *Plant genetics. Early allopolyploid evolution in the post-Neolithic Brassica napus oilseed genome.* Science, 2014. **345**(345): 950-953.
27. Slotte, T., et al., *The Capsella rubella genome and the genomic consequences of rapid mating system evolution.* Nature Genetics, 2013. **45**(7): 831-5.
28. Kitashiba, H., et al., *Draft Sequences of the Radish (Raphanus sativus L.) Genome.* DNA Research, 2014. **21**(5): 481-490.
29. Argout, X., et al., *The genome of Theobroma cacao.* Nature Genetics, 2011. **34**(2): 101-109.
30. Wang, K., et al., *The draft genome of a diploid cotton Gossypium raimondii.* Nature Genetics, 2012. **44**(10): 1098.
31. Xu, Q., et al., *The draft genome of sweet orange (Citrus sinensis).* Nature Genetics, 2012. **45**(1): 59.
32. Huang, S., et al., *The genome of the cucumber, Cucumis sativus L.* Nature Genetics, 2009. **41**(12): 1275-1281.
33. Guo, S., et al., *The draft genome of watermelon (Citrullus lanatus) and resequencing of 20 diverse accessions.* Nature Genetics, 2013. **45**(1): 51-58.
34. Garcia-Mas, J., et al., *The genome of melon (Cucumis melo L.).* Proceedings of the National Academy of Sciences, 2012. **109**(29): 11872-11877.
35. Schmutz, J., et al., *Genome sequence of the palaeopolyploid soybean.* Nature, 2010. **463**(7278): 178-83.
36. Young, N.D., et al., *The Medicago genome provides insight into the evolution of rhizobial symbioses.* Nature, 2011. **480**(7378): 520-524.
37. Varshney, R.K., et al., *Draft genome sequence of chickpea (Cicer arietinum) provides a resource for trait improvement.* Nature Biotechnology, 2013. **31**(3): 240-246.
38. Kang, Y.J., et al., *Draft genome sequence of adzuki bean, Vigna angularis.* Scientific Reports, 2015. **5**: 8069.
39. Vega, J.J.D., et al., *Red clover (Trifolium pratense* L.*) draft genome provides a platform for trait improvement.* Scientific Reports, 2015. **5**.
40. Schmutz, J., et al., *A reference genome for common bean and genome-wide analysis of dual domestications.* Nature Genetics, 2014. **46**(7): 707-713.
41. Mochida, K., et al., *Draft genome assembly and annotation of Glycyrrhiza uralensis, a medicinal legume.* Plant Journal, 2016. **89**(2): 181.
42. Chen, X., et al., *Draft genome of the peanut A-genome progenitor (Arachis duranensis) provides insights into geocarpy, oil biosynthesis, and allergens.* Proceedings of the National Academy of Sciences, 2016. **113**(24).

43. Verde, I., et al., *The high-quality draft genome of peach (Prunus persica) identifies unique patterns of genetic diversity, domestication and genome evolution.* Nature Genetics, 2013. **5**: 487-494.
44. Velasco, R., et al., *The genome of the domesticated apple (Malus × domestica Borkh.).* Nature Genetics, 2010. **42**(10): 833.
45. Wu, J., et al., *The genome of the pear (Pyrus bretschneideri Rehd.).* Genome Research, 2012. **23**(2): 396.
46. Shulaev, V., et al., *The genome of woodland strawberry (Fragaria vesca).* Nature Genetics, 2011. **43**(2): 109.
47. Zhang, Q., et al., *The genome of Prunus mume.* Nature Communications, 2012. **3**(4): 1318.
48. He, N., et al., *Draft genome sequence of the mulberry tree Morus notabilis.* Nature Communications, 2013. **4**(9): 2445.
49. Huang, J., et al., *The Jujube Genome Provides Insights into Genome Evolution and the Domestication of Sweetness/Acidity Taste in Fruit Trees.* Plos Genetics, 2016. **12**(12).
50. Chan, A.P., et al., *Draft genome sequence of the oilseed species Ricinus communis.* Nature Biotechnology, 2010. **28**(9): 951-956.
51. Sato, S., et al., *Sequence Analysis of the Genome of an Oil-Bearing Tree, Jatropha curcas L.* DNA Research, 2011. **18**(1): 65.
52. Bredeson, J.V., et al., *Sequencing wild and cultivated cassava and related species reveals extensive interspecific hybridization and genetic diversity.* Nature Biotechnology, 2016. **34**(5): 562.
53. Wang, Z., et al., *The genome of flax (Linum usitatissimum) assembled de novo from short shotgun sequence reads.* Plant Journal, 2012. **72**(3): 461.
54. Tuskan, G.A., et al., *The Genome of Black Cottonwood, Populus trichocarpa (Torr. & Gray).* Science, 2006. **313**(5793): 1596-1604.
55. Martínez‐García, P.J., et al., *The walnut (Juglans regia) genome sequence reveals diversity in genes coding for the biosynthesis of non‐structural polyphenols.* Plant Journal for Cell & Molecular Biology, 2016. **87**(5): 507.
56. Huang, S., et al., *Draft genome of the kiwifruit Actinidia chinensis.* Nature Communications, 2013. **4**(4): 2640.
57. Wang, L., et al., *Genome sequencing of the high oil crop sesame provides insight into oil biosynthesis.* Genome Biology, 2014. **15**(2): R39.
58. Xiao, L., et al., *The resurrection genome of Boea hygrometrica:A blueprint for survival of dehydration.* Science Foundation in China, 2015. **112**(18): 5833.
59. Consortium, T.P.G.S., *Genome sequence and analysis of the tuber crop potato.* Nature, 2011. **475**(7355): 189-195.
60. Consortium, T.T.G., *The tomato genome sequence provides insights into fleshy fruit evolution.* Nature, 2012. **485**(7400): 635.
61. Kim, S., et al., *Genome sequence of the hot pepper provides insights into the evolution of pungency in Capsicum species.* Nature Genetics, 2014. **46**(3): 270-278.
62. Bombarely, A., et al., *Insight into the evolution of the Solanaceae from the parental genomes of Petunia hybrida.* Nature Plants, 2016. **2**(6): 16074.
63. Hoshino, A., et al., *Genome sequence and analysis of the Japanese morning glory*

Ipomoea nil. Nature Communications, 2016. **7**: 13295.
64. Denoeud, F., et al., *The coffee genome provides insight into the convergent evolution of caffeine biosynthesis.* Science, 2014. **345**(6201): 1181-1184.
65. Iorizzo, M., et al., *A high-quality carrot genome assembly provides new insights into carotenoid accumulation and asterid genome evolution.* Nature Genetics, 2016. **48**(6): 657.
66. Jarvis, D.E., et al., *The genome of Chenopodium quinoa.* Nature, 2017. **542**(7641): p. 307.
67. Dohm, J.C., et al., *The genome of the recently domesticated crop plant sugar beet (Beta vulgaris).* Nature, 2014. **505**(7484): 546-549.
68. Wang, Y., et al., *The sacred lotus genome provides insights into the evolution of flowering plants.* Plant Journal for Cell & Molecular Biology, 2013. **76**(4): 557.
69. Project, A.G., *The Amborella genome and the evolution of flowering plants*. Science, 2013. **342**: 1241089.
70. Chao, A., *Non-parametric estimation of the number of classes in a population*. Scandinavian Journal of Statistics, 1984. **11**: 265-270.
71. Chao, A., et al., *Estimating the number of classes via sample coverage*. Journal of the American Statistical Association, 1992. **87**: 210-217.
72. Chao, A., et al., *Estimating the number of shared species in two communities*. Statistica Sinica, 2000.**10**: 227-246.
73. http://www.pisces-conservation.com/sdrhelp/index.html
74. http://folk.uio.no/ohammer/past/diversity.html
75. http://www.pisces-conservation.com/sdrhelp/index.html
76. https://www.mothur.org/wiki/Coverage
77. http://scikit-bio.org/docs/latest/generated/generated/skbio.diversity.alpha.goods_coverage.html#skbio.diversity.alpha.goods_coverage
78. Faith, D. P., *Conservation evaluation and phylogenetic diversity*. Biol. Conserv. 1992. **61**(1), 1-10.
79. Yan, L. *et al*. *The genome of Dendrobium officinale illuminates the biology of the important traditional Chinese orchid herb*. Molecular Plant, 2015. **8**: 922-934.
80. Tamura, K. *et al*. *MEGA5: molecular evolutionary genetics analysis using maximum likelihood, evolutionary distance, and maximum parsimony methods*. Molecular Biology and Evolution, 2011. **28**, 2731-2739.
81. Schultz, J. *et al*. *SMART, a simple modular architecture research tool: identification of signaling domains*. Proceedings of the National Academy of Sciences, 1998. **95**, 5857-5864.
82. Felsenstein, J. *et al*. *Confidence limits on phylogenies: an approach using the bootstrap*. Evolution, 1985. **39**, 783-791.

Supplementary Note

Gene Ontology (GO) enrichment analysis for each distinct group (Supplementary 11) was illustrated. Cluster 1 and Cluster 2 genes were corresponding to the protocorm and juvenile tuber stages, where *G. elata* start to establish symbiotic relationship with fungi. Several GO categories related to chemical response were enriched in these two stages, which might participate in the symbiotic interaction with fungi partners. After the establishment of steady symbiotic relationship, *G. elata* transit to a fast underground growth stage (immature tuber stage), concordantly, Cluster 3 genes are enriched for GO terms related to active energy processing and cellular growth process. The mature tuber stage is the final underground growth stage of *G. elata*, where *G. elata* finished underground growth and prepare for over ground reproductive growth, Cluster 4 genes are enriched for "response to heat", "vegetative to reproductive phase transition of meristem", make sure the rapid growth of *G. elata* from July to September, and transition to reproductive stage. After emerging from underground, *G. elata* finish its reproductive process within two months, Cluster 5 genes are enriched for GO terms related to nutrition transport and cell wall growth, corresponding to the rapid over ground stem growth process during this stage. Together, these data reveal that *G. elata* consisted of highly dynamic, coordinated and clear timescale transitions of gene expression during its life cycle.
